# Evaluating Long-Term Effectiveness and Safety of Glaucoma Surgical Innovations: A Systematic Review

**DOI:** 10.1097/IJG.0000000000002770

**Published:** 2026-10-16

**Authors:** Sanjana Ashok, Chiara De Vitto, Philip McCullough, Laura Luchetti, Anna Mengozzi, Augusto Azuara-Blanco

**Affiliations:** * Centre of Public Health, Institute of Clinical Science, Queen’s University Belfast, Belfast, UK; † Eye Clinic, Neuromuscular and Sense Organs Department, Careggi University Hospital, Florence, Italy

**Keywords:** glaucoma, surgical innovations, IDEAL collaboration, minimally invasive glaucoma surgery

## Abstract

**Précis:**

A systematic review examining the reporting of long-term safety of glaucoma surgical innovations. Twenty-seven studies met the inclusion criteria and demonstrated effectiveness over the short to medium term. Use of registries to optimise future long-term evaluation is recommended.

**Purpose:**

To synthesise evidence on the long-term effectiveness and safety of glaucoma surgical innovations introduced since 2004 and appraise reporting quality against IDEAL Stage 4 recommendations, designed to promote transparent study of the long-term impact of surgical innovations.

**Methods:**

MEDLINE, EMBASE, and CENTRAL were searched for studies published from January 1, 2014, to October 21, 2024. Eligible studies evaluated novel glaucoma surgery with ≥2 years’ follow-up and ≥100 participants. Randomised controlled trials, observational studies, registry analyses, and case series were included. Reporting was assessed using a 16-item adaptation of the IDEAL Stage 4 checklist. Risk of bias was assessed using Cochrane RoB 2, the Newcastle-Ottawa Scale, and the Joanna Briggs Institute case-series checklist.

**Results:**

The search strategy retrieved 1758 matches, with 27 studies meeting the inclusion criteria. Procedures included iStent, Xen-45 Gel stent, Hydrus microstent, Endoscopic photocoagulation, Trabectome and Kahook dual blade. Follow-up ranged from 2 to 6 years. All studies described design and 22 reported prespecified outcomes sufficiently; however, only 4 explained how missing data were handled. Intraocular-pressure and medication outcomes generally favoured the intervention, but heterogeneity in design, comparators, outcomes, and surgical context precluded pooled estimates. Adverse-event reporting was inconsistent. One cohort study was of poor quality; 14 case series had unclear or unreported methodological items; and 4 RCTs had at least one high-risk domain.

**Conclusions:**

Glaucoma surgical innovations appear effective over the medium term, but limited long-term follow-up and inconsistent safety reporting preclude firm conclusions about comparative effectiveness and long-term safety. Standardised reporting and prospective registries are needed.

Glaucoma is a heterogenous group of diseases characterized by progressive degeneration of retinal ganglion cells, loss of neuro-retinal tissues at the optic nerve head and peripheral visual field loss.^1^ It is a leading cause of irreversible blindness worldwide and is often associated with elevated intraocular pressure (IOP). Glaucoma management aims to lower IOP, slow the rate of visual field deterioration and maintain a patient’s quality of life.^2^ Surgical interventions are vital when medical and laser therapies do not maintain control of the disease. Conventional surgical procedures, such as trabeculectomy and drainage implants have been used for decades.

More recently, various surgical innovations, eg, minimally invasive glaucoma surgery (MIGS) and other advanced drainage devices, have emerged with the potential of an improved safety profile and a bridge between initial medical management and conventional surgical treatment. The European Glaucoma Surgery taskforce paper on surgical innovation has divided these into 2 main forms of surgery—minimally invasive glaucoma surgeries (MIGS) and subconjunctival bleb-forming surgeries.^3^ Examples of both include: iStent, Hydrus, Trabectome, CyPass, Xen-45 Gel Stent, and more. The first MIGS device (iStent) was approved for clinical use in 2004 when it was CE marked.^3^

The main objective of this review was to synthesize evidence on the long-term effectiveness and safety of glaucoma surgical innovations and to appraise the quality of their reporting. The IDEAL (Idea, Development, Exploration, Assessment, and Long-term study) framework was developed by McCulloch et al^4^ to support the staged and transparent evaluation of surgical innovations from first-in-human use through comparative assessment and long-term surveillance as surgical innovations naturally passed through the 5 stages considered above.^5^ The framework provides methodological and reporting recommendations; it does not establish that an intervention is safe or effective. By aligning study design with the stage of development of a surgical innovation, the IDEAL framework is intended to promote prospective and transparent evaluation, facilitate recognition of factors that may influence outcomes such as learning effects and procedural modifications, support comparative assessment once an intervention has stabilized, and enable surveillance for uncommon or delayed adverse events after wider adoption.

IDEAL Stage 4 focuses on outcomes after wider adoption, including durability, changes in the use of a procedure or device, and the identification of uncommon or delayed adverse events through registries and other long-term observational data sources.^5,6^ We therefore used the Stage 4 recommendations to examine how comprehensively the available long-term evidence for glaucoma surgical innovations had been reported.

## METHODOLOGY

We conducted a systematic review. The review protocol was registered on PROSPERO International Prospective Register of Systematic Reviews (https://www.crd.york.ac.uk/prospero), under registration number CRD42024584786. IRB review was not required. The research adhered to the tenets of the Declaration of Helsinki.

### Search Strategy and Sources

The following databases were searched: MEDLINE, EMBASE, and CENTRAL. A list of search terms and search query is provided in Supplementary Table 1, Supplemental Digital Content 1, https://links.lww.com/IJG/B162. The final search across all databases was on October 21, 2024. The reference lists of all included studies were also examined as a source of relevant studies. Covidence software (Veritas Health Innovation) was used for screening and full-text evaluation of literature.^7^

### Eligibility Criteria

We included studies: (i) reporting any glaucoma-related surgical procedure/instrument implemented clinically from 2004 onwards (as this marked the approval of the first MIGS) (ii) published within the last 10 years (between 01 January 2014 to 21 October 2024); (iii) reporting long term outcomes with a minimum of 2 years follow up; (iii) with a sample size of at least 100 participants; and (iv) published in the English language. In terms of study design, we included: interrogations of databases and registries, randomized controlled trials, cohort studies, and observational case series.

We excluded any studies evaluating outcomes of traditional glaucoma surgery, which were implemented into clinical use more than 20 years ago, eg, trabeculectomy, glaucoma drainage devices. The following types of articles were excluded: case reports, review articles, letters, commentaries, published protocols, conference abstracts, and animal-based studies. We excluded studies with <100 participants, as this would enhance statistical power and improve the representativeness of the included studies’ conclusions in identifying prespecified outcomes and looking at unexpected/rare side effects. Including this, the articles from more than 10 years ago were excluded, partly due to minimal studies meeting inclusion criteria during informal literature searching and because authors agreed these studies might not report sufficiently on long-term outcome measures, as most novel procedures were introduced in the preceding decade.

### Study Selection

Duplicates were removed using Covidence and were additionally checked during screening. Titles and abstracts were independently screened by 2 reviewers from the review team (S.A., C.D.V., P.M., L.L., or A.M.). Screening disagreements were resolved by the senior author (A.A.B.).

### Data Extraction

The IDEAL Stage 4 long-term monitoring reporting guideline was adapted into a 16-item data-extraction template (please refer to Table 1).^6^ The items covered 6 reporting domains: title and abstract; introduction; methods; results; discussion; and other information. Specific items included identification of study design, review of prior evidence, statement of objectives and prespecified outcomes, description and governance of the data set, eligibility criteria, description of the intervention and comparator (if applicable), statistical methods, handling of missing data, participant and center/operator characteristics, limitations, ethical approval, funding, and regulatory status. Each item was rated “Yes” when the requested information was reported clearly and in sufficient detail, “Partially Yes” when the information was present but incomplete or ambiguous, and “No” when it was not reported. The reason for each judgment was recorded.

**TABLE 1:** Modified IDEAL Stage 4 Checklist for Data Extraction (Found on IDEAL Collaboration’s Website. Each Checklist Item was Rates Y or Yes, PY or Partially Yes and N or No During Data Extraction^5^

Title and abstract	1a. Does the study identify the study design and/or the IDEAL stage in the title or abstract? 1b. Does the abstract provide a structured summary of background, methods, results, and conclusions?
Introduction, eg, background and objectives	2a. Does the study review existing scientific literature, including reference to earlier IDEAL stage reports in previous publications, if applicable? 2b. Are specific objectives stated, which may include recognizing late or uncommon safety outcomes, identifying changes in the use of procedure/device, risk adjustment, quality assurance, and effectiveness?
Methods, ie, design, data source, participants, intervention, outcomes, statistical methods	3. Does the methodology describe the study design (eg, registry, analysis of real-world data, etc.)? 4a. Is there a description of the data set, including who designed and funds it and who curates and manages it, addressing possible conflicts of interest? Was the data set purposely designed for research or collected for other purposes? 4b. Are structured data fields described or referenced and each item defined, included as an appendix or supplement when necessary? 5. Is there a detailed account of inclusion and exclusion criteria for subjects? 6. Is there a description or reference to the intervention/device being monitored, and comparator if applicable? 7. Is there a description of prespecified primary and secondary outcome measures selected and how they will be assessed, including patient reported outcome measures, when appropriate, utilizing those measures that are standardized and validated, when available and applicable? 8a. Were statistical methods used to describe and assess patient characteristics and outcomes, including methods of additional analysis (eg, prespecified subgroup analysis), and methods to minimize confounding, where appropriate? 8b. Was there an explanation for how missing data was addressed?
Results	9a. Is there a description of patient demographic and clinical characteristics, and hospital/center/operator characteristics? 9b. Do results Indicate extent of missing data for each variable of interest, if appropriate? 10. Do results report main prespecified outcome measures, including outcome variations among prespecified subgroups and adjustments for confounders, when applicable?
Discussion	11. Is there discussion of limitations, addressing sources of potential bias, known confounders, missing data, and secular trends, including a discussion of the implications of using data that was not originally collected to answer the specific research question, where appropriate? 12. Is there evidence of conclusions and relevance, including a discussion of the overall interpretation of results, the external validity, and any implications for policy and practice?
Other information	13. Please quote reference or DOI if a protocol was written in advance and made available 14. Was appropriate ethical approval obtained and informed consent process described. If consent for future use of data was not obtained, explanation provided? 15. Have sources of funding and support, role of funders, and other conflicts of interest been reported? 16. Are regulatory approvals being sought or obtained (eg, CE Marking, FDA approval, etc.)?

Data from each included study was extracted by one reviewer and independently checked by a second reviewer. Disagreements were resolved through discussion and, when consensus could not be reached, by adjudication from the senior author.

### Risk of Bias Assessment

Risk of bias was also assessed using the following tools: Cochrane RoB 2.0 for RCTs,^8^ Newcastle-Ottawa Scale (NOS) for observational studies,^9^ and the Joanna Briggs Institute (JBI) tool for case series.^10^ The risk-of-bias tool was selected according to the design described in each publication. Where an article was described only as “retrospective” and did not report a clearly defined comparative cohort, it was assessed using the JBI case-series checklist.

NOS assesses nonrandomised studies on a scale from 0 to 9 based on the selection of cases and controls, comparability of cases and controls, and ascertainment of exposure. Scores ≥7 indicate high quality, while scores ≤4 indicate low quality. Cochrane RoB 2.0 evaluates bias arising from randomization, deviations from intended interventions, missing outcome data, outcome measurement, and selective reporting; the overall judgment was determined by the most severe domain-level rating. The JBI checklist for case series evaluates the clarity of eligibility criteria, case identification and measurement, consecutive and complete inclusion, reporting of clinical and demographic characteristics and outcomes, and appropriateness of statistical analysis.

Risk-of-bias assessments were completed by one reviewer and independently checked by a second reviewer, with disagreements resolved using the same process as data extraction.

### Data Synthesis and Analysis

The data extracted were put into data synthesis tables for each section of the checklist, results available in Supplementary Materials (Supplementary Tables 2–8, Supplemental Digital Content 2, https://links.lww.com/IJG/B163, Supplemental Digital Content 3, https://links.lww.com/IJG/B164, Supplemental Digital Content 4, https://links.lww.com/IJG/B165, Supplemental Digital Content 5, https://links.lww.com/IJG/B166, Supplemental Digital Content 6, https://links.lww.com/IJG/B167, Supplemental Digital Content 7, https://links.lww.com/IJG/B168, Supplemental Digital Content 8, https://links.lww.com/IJG/B169).

## RESULTS

### Search Results

A search on October 21, 2024, identified 1758 publications, with 426 duplicates removed automatically and 3 removed manually. Seven additional abstracts were found through citation searching. After screening of abstracts, 1211 studies were excluded, leaving 119 for full-text review. Of these, 90 were excluded (reasons available in PRISMA diagram, Fig. 1), leaving 27 eligible studies.

**FIGURE 1:**
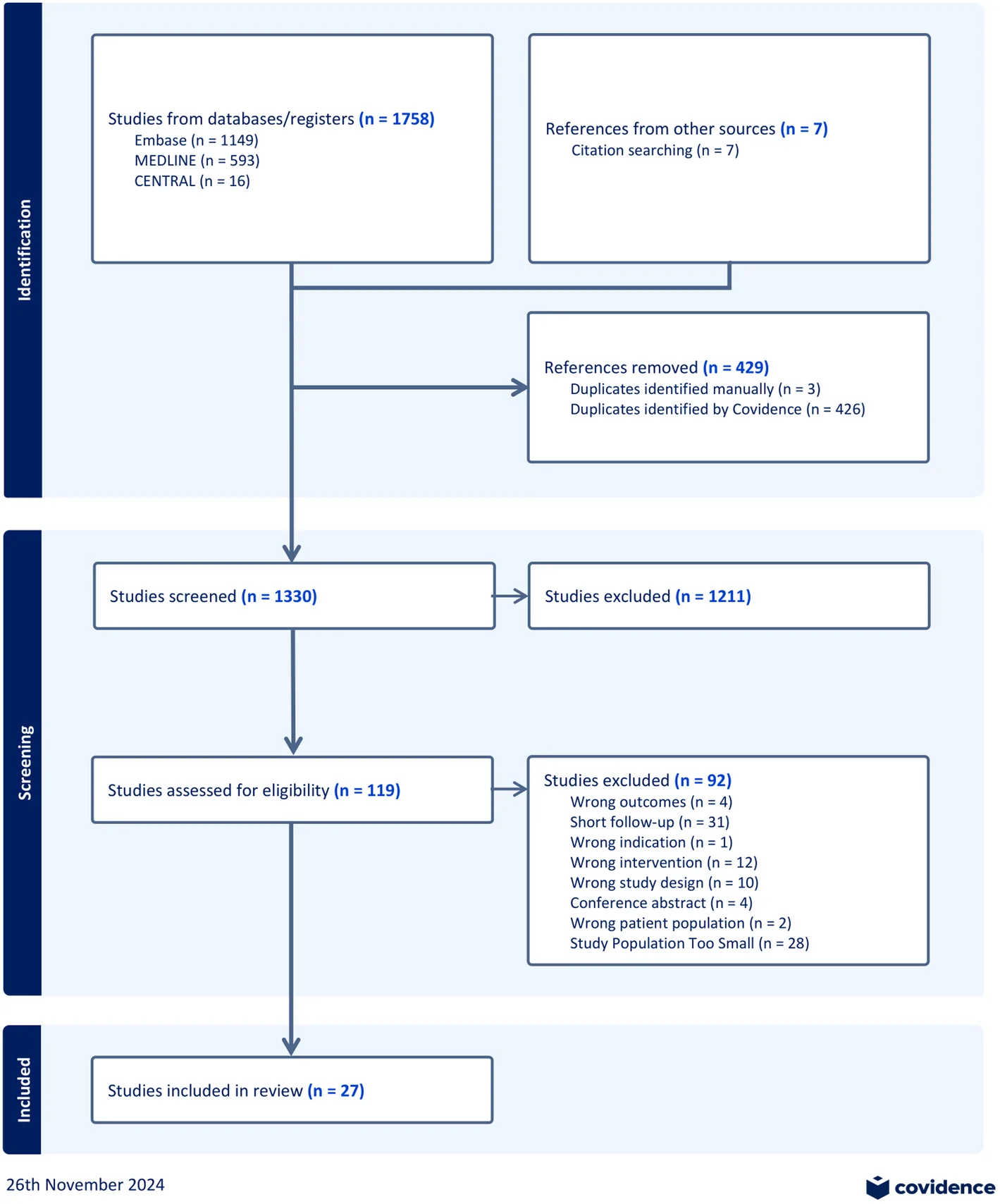
PRISMA diagram demonstrating the screening and extraction process of studies. Reasons for the removal of full-text articles are provided.

### Study Characteristics

Data originated from various countries including: Armenia (1 study),^11^ Australia (3 studies),^12–14^ Canada (1 study),^15^ China (1 study),^16^ Germany (2 studies),^17,18^ Japan (2 studies),^19,20^ Switzerland (1 study),^21^ United Kingdom (2 studies)^22,23^ and United States of America (11 studies).^24–34^ One study was an international collaboration between Austria, Belgium, UK, and Spain.^35^ Another was based in Austria, Belgium, Canada and Germany.^36^ Another was a collaboration between France and Australia.^37^

### Risk of Bias

Detailed study-level risk-of-bias assessments are provided in Supplementary Tables 9–11, Supplemental Digital Content 9, https://links.lww.com/IJG/B170, Supplemental Digital Content 10, https://links.lww.com/IJG/B171, Supplemental Digital Content 11, https://links.lww.com/IJG/B172. Among the 4 comparative observational studies assessed using the Newcastle-Ottawa Scale, 3 were rated as good quality^26,30,36^ and one as poor quality^18^ because of concerns relating to cohort selection and outcome ascertainment.

Of the 17 case series, 3 satisfied all assessed JBI criteria,^15,17,33^ whereas 14 had at least one unclear or unreported item, most commonly relating to eligibility criteria, consecutive or complete inclusion, consistent measurement of the condition, or reporting of the study setting.^12–14,16,19–24,27,34,35,37^

The Cochrane risk of bias tool for RCTs was used for 6 studies (Supplementary Table 11, Supplemental Digital Content 11, https://links.lww.com/IJG/B172).^11,25,28,29,31,32^ Two of these— Montesano et al^25^ and Ahmed et al^31^ extracted results from the same RCT. Four studies had one domain judged at high risk of bias: 2 because of the randomization process^11,32^ and 2 studies from the same RCT data because of outcome measurement.^25,31^

### Surgical Innovations

Studies evaluated either a single innovation or a combination of innovations. Study characteristics and surgical innovations/devices are summarized in Table 2. Procedures were performed as either standalone surgery or in combination with phacoemulsification. Because several comparative and registry studies included more than one procedure, their findings were interpreted according to the relevant surgical context rather than pooled across devices.

**TABLE 2:** Studies Organized by Authors+Year, Country of Research and Anatomic Target for Lowering Intraocular Pressure

References	Country of research	Innovation/device
Trabecular bypass and Schlemm canal scaffolding		
Ahmed et al^32^	USA	iStent inject
Clement et al^12^	Australia	iStent inject + Cataract surgery
Ferguson et al^34^	USA	iStent
Hengerer et al^17^	Germany	iStent inject +/− Cataract surgery
Holmes et al^13^	Australia	iStent inject or Hydrus Microstent
Inatani et al^19^	Japan	iStent + Cataract Surgery
Katz et al^11^	Armenia	iStent
Lee et al^23^	UK	iStent + Cataract surgery or Hydrus Microstent + Cataract Surgery
Salimi et al^15^	Canada	iStent inject + Cataract surgery
Samuelson et al^28^	USA	iStent inject
Samuelson et al^29^	USA	iStent inject
Ahmed et al^31^	USA	Hydrus Microstent + Cataract Surgery
Montesano et al^25^	USA	Hydrus Microstent + Cataract Surgery
Subconjunctival bleb forming procedure		
Arnould et al^37^	France; Australia	XEN-45 Gel stent
Bambo et al^21^	Switzerland	XEN-45 Gel Stent + Cataract Surgery
Gabbay et al^22^	UK	XEN-45 gel stent
Reitsamer et al^35^	Austria, Belgium, UK, Spain	XEN-45 Gel stent
Schlenker et al^36^	Austria; Belgium; Canada, Germany	XEN-45 Gel Stent with mitomycin C (MMC)
Vukmirovic et al^14^	Australia	XEN-45 Gel Stent
Trabecular meshwork ablation/excision		
Dang et al^16^	China	Trabectome
Strzalkowska et al^18^	Germany	Trabectome with Mitomycin C (MMC)
Tojo and Hayashi^20^	Japan	Trabectome
Comparative or combined procedures		
Deitz et al^33^	USA	Endoscopic cyclophotocoagulation (ECP) + Cataract Surgery or iStent + Cataract surgery
Mitchell et al^24^	USA	abMIGS (Canaloplasty, Goniotomy, Trabectome, iStent, Hydrus Microstent) with and without prior laser trabeculoplasty (<2 y preceding MIGS)
Oberfeld et al^26^	USA	PEcK(which combines Phacoemulsification, Endo cyclophotocoagulation, and Kahook dual blade); against its individual components (Phaco/ECP and Phaco/KDB)
Oberfeld, Helwe et al^27^	USA	Hydrus Microstent + Cataract Surgery or Kahook Dual Blade + Cataract Surgery
Yang et al^30^	USA	Endoscopic cyclophotocoagulation (ECP) or XEN 45 Gel stent or Goniotomy/Canaloplasty

These surgical innovations can be grouped into Trabecular bypass or Schlemm canal procedures that enhance conventional aqueous outflow, trabecular excisional or ablative procedures that remove outflow resistance, subconjunctival bleb-forming devices that create an alternative drainage pathway, cyclodestructive procedures that reduce aqueous production, and combined procedures that use more than one of these mechanisms.

Thirteen studies evaluated angle-based trabecular bypass MIGS like iStent, iStent inject or Hydrus Microstent,^11–13,15,17,19,23,25,28,29,31,32,34^ 6 studies evaluated the XEN-45 gel stent,^14,21,22,35–37^ 3 evaluated Trabectome^16,18,20^ and 5 studies evaluated multiple devices or a combination of multimodal procedures.^24,26,27,30,33^

### IDEAL Stage 4 Framework

Figure 2 summarizes reporting against the 16 adapted IDEAL Stage 4 items. All 27 studies^11–37^ provided a structured abstract, and 23 clearly identified their study design in the title or abstract.^12,14,16,17,19–37^ Nineteen adequately reviewed the existing literature^12,13,15,17,20–25,27–33,35,37^ and 25 stated specific objectives.^11,12,14–17,19–37^ All studies described their design and statistical methods.

**FIGURE 2:**
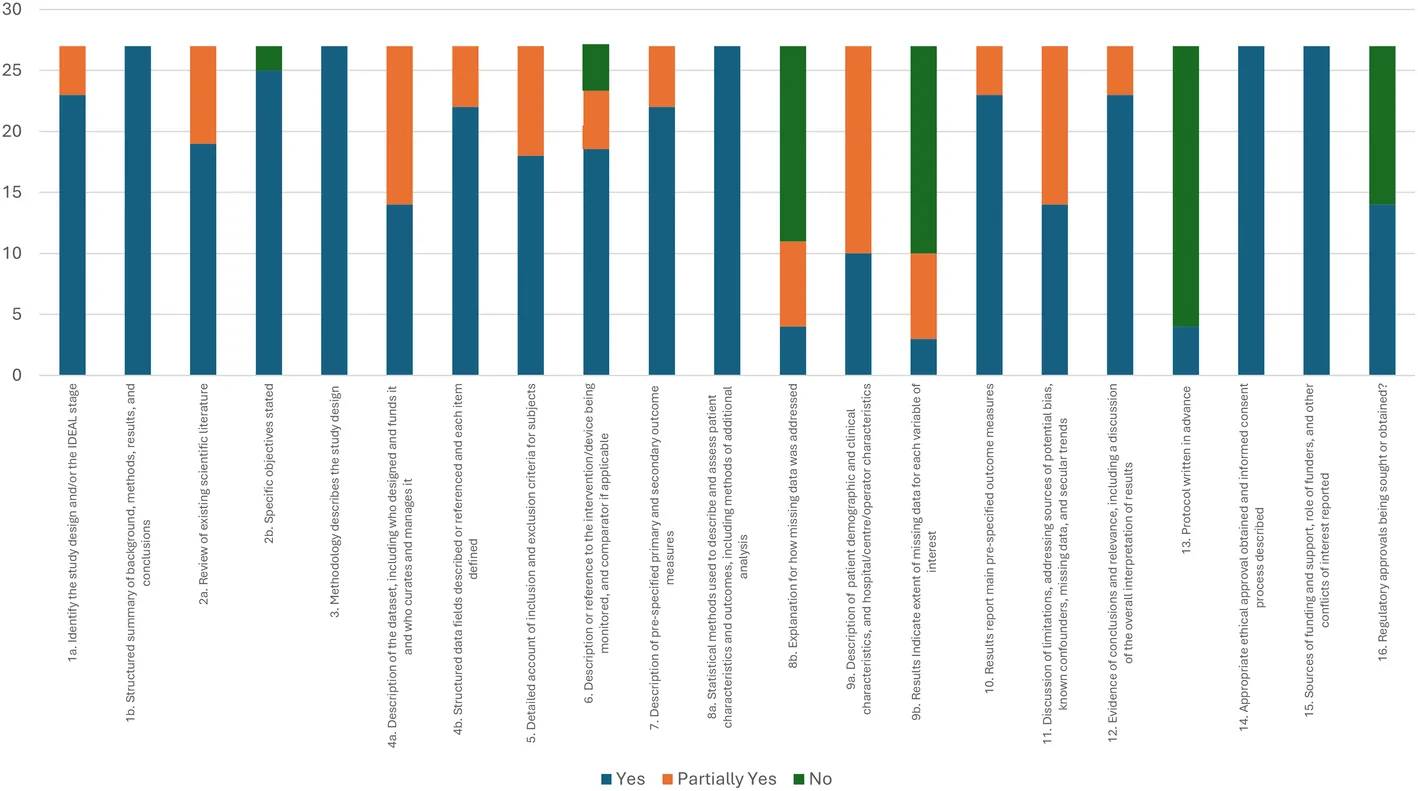
A Stacked Bar chart demonstrating adherence of studies to each IDEAL Stage 4 checklist item. Blue = Yes, Orange = Partially Yes, Green = No.

Fourteen fully described the source, governance, and funding of the data set;^11,12,14,17,20,24,25,28,30–33,36,37^ 22 described the data fields;^12–17,19–33,36^ 18 provided detailed eligibility criteria;^12–17,21–28,31,32,35,36^ 20 adequately described the intervention or comparator;^11,12,14–23,26–28,32,34–36^ and 22 specified primary and secondary outcomes and how they would be assessed.^11,12,14–16,18–22,24–28,30–34,36,37^

Handling and reporting of missing data were the weakest areas. Only 4 studies fully explained the statistical handling of missing data,^21,26,29,30^ 7 did so partially,^19,23–25,32,36,37^ and 16 provided no explanation.^11–18,20,22,27,28,31,33–35^ Ten studies fully described participant, center, and operator characteristics,^12,14,15,17,20,22,23,26,33,36^ while only 3 fully quantified missing data for each relevant variable.^25,30,36^ Twenty-three studies fully reported their prespecified outcomes.^12–17,19–28,30,31,33–37^ Fourteen adequately discussed limitations and potential sources of bias,^13,15,20,22,24–27,29,30,33,35–37^ and 23 discussed the conclusion and relevance of their findings.^11–13,15,17–30,33,35–37^ Six reports were linked to an advance protocol.^11,25,28,29,31,32^ All studies reported ethical approval and funding, and 14 referred to relevant regulatory approval (eg, CE marking, FDA approval).^11,13,15–19,21,28–30,32,34,37^

### Effectiveness Measures

Four studies reported outcomes for 5 years.^17,25,31,32^ Two studies looked at outcomes over 4 years,^21,36^ 1 study over 3.5 years,^11^ 7 studies over 3 years^15,22,24,26,27,30,35^ and 12 studies reported outcomes for 2 years.^12–14,16,18–20,23,28,29,33,37^

#### Trabecular Bypass and Schlemm Canal Devices

Studies of iStent, iStent inject, and the Hydrus Microstent reported follow-up from 2 to 6 years and generally described reductions in intraocular pressure and/or glaucoma medication use.^11–13,15,17,19,23–25,28,29,31,32,34^ Reported longer-term device-related findings included peripheral anterior synechiae or tissue adhesions, stent malposition or obstruction, and changes in endothelial cell density; however, definitions and reporting time points were inconsistent.

#### Subconjunctival Bleb-Forming Procedures

Six studies reported outcomes over 2–4 years.^14,21,22,35–37^ Intraocular-pressure and medication reductions were reported in both standalone and combined phacoemulsification procedures. Safety outcomes included hypotony, bleb needling or revision, device exposure or erosion, device replacement, and the need for additional glaucoma surgery. The frequency and timing of these outcomes were not reported consistently enough to support a pooled estimate.

#### Trabecular Ablation or Excision

Trabectome studies reported 2-year outcomes, while studies of endoscopic cyclophotocoagulation, Kahook Dual Blade, and mixed registry-based procedures reported follow-up of 2–3 years.^16,18,20,24,26,27,30,33^ Reported intraocular-pressure and medication outcomes were generally favorable. Adverse events included transient blood reflux or hyphaema, inflammation, corneal edema, and occasional further glaucoma surgery, but event definitions and denominators varied.

Across all procedure groups, heterogeneity in study design, definitions of success, reporting of additional surgery, and attribution of adverse events prevented comparative effectiveness analysis or reliable estimation of uncommon and delayed complications. Detailed study-level safety outcomes are provided in Supplemental Table 8, Supplemental Digital Content 8, https://links.lww.com/IJG/B169.

## DISCUSSION

This systematic review highlights the diversity in study designs evaluating long-term outcomes on surgical innovations for glaucoma. We observed a mixture of methodologies, including case series, exploratory analyses derived from RCTs, retrospective cohort studies, and registry-based analyses. This variety presents challenges in synthesizing evidence on the effectiveness and safety of these surgical interventions. Overall, although studies reported that the surgical innovations are effective IOP lowering interventions with a good safety profile, there was only a small number of studies—5 with follow-up of 5 or more years, all with different varying methodologies.

The IDEAL framework underscores the importance of utilizing prospective, registry-based data to analyse surgical outcomes comprehensively. Such designs enhance the robustness of findings by reducing bias, controlling for confounders, and improving external validity. Prospective registries allow for standardized data collection and facilitate long-term follow-up, which is essential for understanding the full spectrum of surgical complications and successes. Gedde et al^38^ describe the importance of standardizing methodology to enhance interpretation and comparison across different studies evaluating MIGS, stating that a valuable assessment of clinical endpoints should include the concept of minimal clinically important difference (MCID), which is the smallest change in a treatment outcome considered beneficial to individual patients.

The American Academy of Ophthalmology’s Glaucoma Preferred Practice Committee also recommends the use of cumulative probability of surgical success (defined as IOP <21 mm Hg or decrease in glaucoma medical therapy depending on standalone MIGS or MIGS alongside cataract surgery) at 2 years with Kaplan-Meier survival analysis as the primary endpoint in MIGS studies.^38^ This approach has its limitations, as IOP is a surrogate measure for disease control and 21 mm Hg is an arbitrary threshold value. Another measure of clinical endpoints could be visual fields, however these have not been fully defined within guidelines yet.^39^

The lack of a uniform design and clinical endpoints among the included studies within this review limits the ability to draw definitive conclusions around comparative efficacy and safety. This also affects the generalizability of findings across different patient populations and clinical settings.

A further limitation of this review was the inability to perform a meta-analysis. Although key outcomes such as intraocular pressure, number of glaucoma medications, need for additional glaucoma surgery, and postoperative complications were reported across the included studies, they were defined and measured inconsistently. Studies differed substantially in design, surgical procedure, whether the intervention was performed alone or alongside cataract surgery, duration of follow-up, baseline disease severity, comparator groups, and definitions of surgical success and failure. Adverse events were also reported using variable terminology, time points, and denominators. This heterogeneity precluded meaningful statistical pooling and limited our ability to generate summary effect estimates or directly compare the relative effectiveness and safety of different procedures. Consequently, the findings were synthesized narratively, which is more susceptible to subjective interpretation and provides less precise estimates than a formal meta-analysis. These limitations reduce the certainty and generalizability of the conclusions and highlight the need for greater standardization of outcome definitions and reporting in future studies of glaucoma surgical innovations.

Yuan et al^40^ performed a systematic review and meta-analysis analysing the combined MIGS-phacoemulsification with phacoemulsification alone. Secondary outcomes for their study included measuring complication rates. Our study findings match their conclusion on the heterogeneity of adverse event reporting to be a limitation for drawing conclusions.

A significant limitation noted across the studies pertains to the reporting of missing data. Many studies did not adequately address how missing data was handled, which raises concerns about the validity of the reported outcomes. Improved transparency in data reporting and handling of missing information is crucial for enhancing the overall quality of evidence in this field.

Another point to consider is that while we set a minimum sample size of 100 subjects per study, these studies may still lack the necessary sample size to reliably determine frequency of uncommon complications. The inclusion of registry studies mitigates some of these concerns, as they tend to offer larger and more diverse patient cohorts, enabling a more comprehensive assessment of outcomes. Another limitation is the lack of longer-term data, as 2-year follow-up is a relatively short period of time for patients with a chronic condition with glaucoma and might not be a suitable marker of long-term outcomes.

In conclusion, while the current review sheds light on the varied landscape of surgical innovations in glaucoma, the paucity of studies describing long-term follow-up outcomes, differences in study design and limitations regarding data reporting pose challenges for decision makers. Future research should aim for more standardized methodologies, particularly in prospective registry settings, to enhance the reliability and applicability of results.

## Supplementary Material
