# Supplementary material for: Evaluating Long-Term Effectiveness and Safety of Glaucoma Surgical Innovations: A Systematic Review
Source: J Glaucoma. 2026 Oct 16;35(11):838–45. doi: 10.1097/IJG.0000000000002770 (PMC13722426; doi:10.1097/IJG.0000000000002770)
Supplement: Supplementary file 1 [file ijg-35-838-s001.pdf]

Supplementary Table 1. *Details of Search strategy across EMBASE, MEDLINE and CENTRAL*

Date of Search: 21/10/2024

Search terms included: “Glaucoma”, “MIGS”, “minimally invasive glaucoma surgery” “iStent”, “Hydrus”, “Innfocus”, Preserflo Microshunt”, “Preserflo”, “Endophotocoagulation”, “GATT”, “gonioscopy-assisted transluminal trabeculotomy” “Canaloplasty”, “Trabectome”, “Endoscopic Cyclophotocoagulation”, “XEN Gel Stent”, “Ab interno canaloplasty” “ABiC”, “Kahook”, “iStent Supra”, “CyPass”, “MINIject”, “TRAB360”, “VISCO360”, “OMNI”, “Streamline”, “GMS”, “HFDS”, “STT ab interno”, “SOLX”, “STARflo”, “Aquashunt”, “Excimer laser trabeculostomy”, “IRIS registry”, “Medisoft”, “National audit”, “OpenEyes”, “Electronic Medical Record”, “Registry”, “database”, “outcomes”, “long-term outcomes”, “IDEAL”, “IDEAL Collaboration”

	EMBASE	MEDLINE	CENTRAL
1. -	Glaucoma*	Glaucoma*	Glaucoma*
2.	MIGS	MIGS	MIGS or "Minimally invasive glaucoma surgery" or iStent or Hydrus or Innfocus or "Preserflo Microshunt” or Preserflo or Endophotocoagulation or "gonioscopy-assisted transluminal trabeculotomy" or GATT or Canaloplasty or Trabectome or “Endoscopic Cyclophotocoagulation” or “XEN Gel Stent” or "Ab Interno Canaloplasty" or ABiC or Kahook or iStent Supra or CyPass or MINIject or TRAB360 or VISCO360 or OMNI or Streamline”or GMS or HFDS or "STT ab interno” or SOLX or STARflo” or Aquashunt or “Excimer laser trabeculostomy”
3.	Glaucoma drainage implant* or Glaucoma Surgery* or Minimally invasive glaucoma surgery	Glaucoma drainage implants* or Minimally invasive glaucoma surgery	"IRIS registry” or Medisoft or "National audit” or openeyes or “Electronic Medical Record” or Registry or database or "long term outcomes" or IDEAL or "IDEAL collaboration"
4.	iSTENT	iSTENT	#1 AND #2 AND #3 with Cochrane Library publication date Between Jan 2014 and Aug 2024
5.	Hydrus	Hydrus	Date limit 2014 to 2024
6.	Innfocus	Innfocus	
7.	Preserflo Microshunt	Preserflo microshunt	
8.	Preserflo	Preserflo	
9.	Endophotoccoagulation or laser coagulation*	Endophotocoagulation or Laser coagulation*	
10.	gonioscopy-assisted transluminal trabeculotomy* or GATT	gonioscopy-assisted transluminal trabeculotomy	
11.	Gonioscopy-assisted transluminal trabeculotony	GATT	
12.	Canaloplasty*	Canaloplasty	
13.	Trabectome	Trabectome	
14.	Endoscopic Cyclophotocoagulation	Endoscopic Cyclophotocoagulation	
15.	ABiC	ABiC	
16.	Kahook	Kahook	
17.	iStent Supra	iStent Supra	
18.	CyPass or nonvalved ophthalmic drainage device*	CyPass	
19.	MINIject	MINIject	
20.	TRAB360	TRAB360	

21.	VISCO360	VISCO360	
22.	OMNI	OMNI	
23.	Streamline	Streamline	
24.	GMS	GMS	
25.	HFDS	HFDS	
26.	STT ab interno	STT ab interno	
27.	STARflo	STARflo	
28.	Aquashunt	Aquashunt	
29.	SOLX	SOLX	
30.	Excimer laser trabeculostomy	Excimer laser trabeculostomy	
31.	IRIS registry	IRIS registry	
32.	Medisoft	Medisoft	
33.		National audit	
34.		OpenEyes	
35.	Medical audit* or clinical audit* or electronic medical record*	Electronic health records*	
36.	OpenEyes	Registries* or registry	
37.	Electronic medical record or medical record* or Electronic medical records*	database	
38.	Electronic health record*	Patient reported outcome measures* or outcomes or Treatment outcome* or Patient Outcome assessment*	
39.	Registry or Register*	Long-term outcomes	
40.	Database or data base*	IDEAL	
41.	Outcomes or patient reported outcome measures* or Treatment outcome* or Patient Outcome Assessment*	IDEAL collaboration	
42.	Long-term outcomes	2 or 3 or 4 or 5 or 6 or 7 or 8 or 9 or 10 or 11 or 12 or 13 or 14 or 15 or 16 or 17 or 18 or 19 or 20 or 21 or 22 or 23 or 24 or 25 or 26 or 27 or 28 or 29 or 30	
43.	IDEAL	31 or 32 or 33 or 34 or 35 or 38 or 39 or 40 or 41	
44.	IDEAL collaboration	1 and 42 and 43	
45.	2 or 3 or 4 or 5 or 6 or 7 or 8 or 9 or 10 or 11 or 12 or 13 or 14 or 15 or 16 or 17 or 18 or 19 or 20 or 21 or 22 or 23 or 24 or 25 or 26 or 27 or 28 or 29 or 30	Date limit 2014 to 2024	
46.	31 or 32 or 33 or 34 or 35 or 36 or 37 or 38 or 39 or 40 or 41 or 42		
47.	1 and 43 and 44		
48.	Date limit 2014 to 2024		
