# Supplementary material for: Evaluating Long-Term Effectiveness and Safety of Glaucoma Surgical Innovations: A Systematic Review
Source: J Glaucoma. 2026 Oct 16;35(11):838–45. doi: 10.1097/IJG.0000000000002770 (PMC13722426; doi:10.1097/IJG.0000000000002770)
Supplement: Supplementary file 2 [file ijg-35-838-s002.pdf]

Supplementary Table 2. Data extraction as per IDEAL Stage 4 Framework for Title and Abstract (rated Yes, Partially Yes or No)

	**Title and abstract**	
**Authors + Year**	**1a. Does the study identify the study design and/or the IDEAL stage in the title or abstract? (Y, PY, N)**	**1b. Does the abstract provide a structured summary of background, methods, results, and conclusions? (Y, PY, N)**
Ahmed et al 2022	Y - The abstract describes the study design as a "Prospective, multicenter, controlled randomized clinical trial". There is no mention of IDEAL stage in the title or abstract.	Y - Clearly structured abstract
Ahmed et al 2023	Y - The abstract describes the study as a "5-year follow-up safety study of the prospective, randomized, single-masked, concurrently controlled, multicenter iStent inject pivotal trial".	Y - Clearly structured abstract
Arnould et al 2024	Y - The study is described as a "retrospective analysis of prospectively collected data". This indicates an observational study design, which would typically fall under the 'long-term follow-up' or 'registry' stage of the IDEAL framework.	Y - Clearly structured abstract
Bambo et al, 2024	Y - The study is a retrospective and single-center study. This information is stated in the abstract. There is no mention of "IDEAL stage" in the title or abstract.	Y - Clearly structured abstract
Clement et al 2020	Y - Abstract states that this is a retrospective study. There is no mention of "IDEAL stage" in the title or abstract.	Y - Clearly structured abstract
Dang et al 2018	Y - The abstract mentions that it is a "multicentre, retrospective, observational study" and this is also specified in the title. There is no mention of "IDEAL stage" in the title or abstract.	Y - Clearly structured abstract
Deitz et al 2023	Y - The abstract describes the study as a retrospective chart review. There is no mention of "IDEAL stage" in the title or abstract.	Y - Clearly structured abstract
Ferguson et al 2020	Y - The study design is identified in the abstract as a "Retrospective, consecutive case series". There is no mention of "IDEAL stage" in the title or abstract.	Y - Clearly structured abstract
Gabbay et al 2021	Y - The abstract states that the study is a "retrospective non-comparative audit".There is no mention of "IDEAL stage" in the title or abstract.	Y - Clearly structured abstract
Hengerer et al 2022	Y - The study design is identified as a prospective longitudinal case series in the abstract.The terms "IDEAL" or "stage" do not appear in the title or abstract of this study	Y - Clearly structured abstract
Holmes et al 2022	PY - Abstract describes this as a comparative study. Analysis of data from the Fight Glaucoma bindness registry. However, whether this is retrospective or prospective is not made clear.	Y - Clearly structured abstract
Inatani et al 2022	Y - The abstract does state that it is a "2-year prospective, longitudinal, observational, post-marketing surveillance study". This implies that the study is likely in the later stages of the IDEAL framework because the device is already approved for use and being monitored for its performance in real-world settings.	Y - Clearly structured abstract
Katz et al 2018	PY - Abstract states that it is a "prospective randomized ongoing study" but does not give information to determine if it is a randomised controlled trial.	Y - Clearly structured abstract
Lee et al 2020	Y - The abstract mentions it is a "Retrospective single surgeon comparative case series". However, the title and abstract do not explicitly mention the IDEAL stage of the study	Y - Clearly structured abstract
Mitchell et al 2024	Y - The abstract indicates that it is a "Retrospective cohort study". However, there is no explicit mention of an IDEAL stage in either the title or the abstract.	Y - Clearly structured abstract
Montesano et al 2023	Y - The study is described as a "post hoc analysis of visual field data from a randomized controlled trial." There is no mention of "IDEAL stage" in the title or abstract.	Y - Clearly structured abstract
Oberfeld et al 2023	Y - Study defined as a "Retrospective study" in abstract. There is no mention of "IDEAL stage" in the title or abstract.	Y - Clearly structured abstract
Oberfeld, Helwe et al 2023	Y - The abstract identifies the study design as a "Retrospective study". However, there is no mention of the IDEAL stage in the title or abstract.	Y - Clearly structured abstract
Reitsamer et al 2022	Y - The abstract identifies the study as a "Retrospective chart review". There is no mention of "IDEAL stage" in the title or abstract.	Y - Clearly structured abstract

Salimi et al 2021	PY - The title of the study specifies that it is a "longitudinal case series" but it doesn't refer to an IDEAL stage. It also does not clarify if this is a prospective or retrospective study.	Y - Clearly structured abstract
Samuelson et al 2019	Y - The study is described as a "Prospective, Randomized, Controlled Pivotal Trial" in the title. There is no mention of "IDEAL stage" in the title or abstract.	Y - Clearly structured abstract
Samuelson et al 2021	Y - The study's design is identified in the abstract as a "Randomized controlled trial analysis of secondary outcomes" looking at patient reported outcomes. The title also refers to the study as an "analysis from the iStent inject® pivotal trial"	Y - Clearly structured abstract
Schlenker et al 2017	Y - The abstract does identify the study design. It is described as an "International, multicenter, retrospective interventional cohort study"	Y - Clearly structured abstract
Strzalkowska et al 2021	PY - Does not explicitly define study type but mentions that this is an exact-matched study for comparison. Abstract does not make it clear if this is prospective/retrospective.	Y - Clearly structured abstract
Tojo et al 2020	Y - The study is described as a single-facility, nonrandomized, observational retrospective study. No reference to IDEAL stage.	Y - Clearly structured abstract
Vukmirovic et al 2023	Y - This is a retrospective, observational study. There is no reference to IDEAL stage in the title or abstract.	Y - Clearly structured abstract
Yang et al 2023	Y - The abstract mentions the study design as a "Multicenter, retrospective cohort study"	Y - Clearly structured abstract
