# Supplementary material for: Evaluating Long-Term Effectiveness and Safety of Glaucoma Surgical Innovations: A Systematic Review
Source: J Glaucoma. 2026 Oct 16;35(11):838–45. doi: 10.1097/IJG.0000000000002770 (PMC13722426; doi:10.1097/IJG.0000000000002770)
Supplement: Supplementary file 3 [file ijg-35-838-s003.pdf]

Supplementary Table 3. Data extraction as per IDEAL Stage 4 Framework for Introduction (rated Yes, Partially Yes or No)

-	**Introduction eg. background and objectives**	
**Authors + Year**	**2a. Does the study review existing scientific literature, including reference to earlier IDEAL stage reports in previous publications, if applicable? (Y, PY, N)**	**2b. Are specific objectives stated, which may include recognizing late or uncommon safety outcomes, identifying changes in the use of procedure/device, risk adjustment, quality assurance, and effectiveness? (Y, PY, N)**
Ahmed et al 2022	Y - The study does review existing scientific literature. It references previous studies on the Hydrus Microstent (HMS), noting that prior randomized studies showed significant reductions in IOP and medication use in patients with primary open-angle glaucoma. It also cites studies demonstrating that the HMS device increases outflow facility. There is a reference to previous 3-year findings from the HORIZON trial	Y - The study aims to provide 5-year safety and effectiveness results, including the impact on the corneal endothelial cell layer. The study also aims to assess long-term outcomes, including surgical success rate, mean IOP, need for glaucoma medical therapy, repeat glaucoma surgery, loss of visual acuity, visual field progression, procedure-related adverse events, and corneal endothelial cell counts
Ahmed et al 2023	Y - While it does cite prior clinical and pivotal trials related to the iStent inject, there are no explicit references to the IDEAL framework or reports categorised by IDEAL stages.	Y - The objective of the current study is to report long-term corneal endothelial safety data of iStent inject, including ECL, up to 60 months postoperatively.
Arnould et al 2024	Y - The study reviews existing scientific literature. The introduction cites previous studies on the Xen 45 gel stent and other filtration surgeries. The discussion section also refers to other studies, noting different definitions of success and varied outcomes.	Y - The primary objective was to report 24-month real-world safety and efficacy outcomes of the Xen 45 gel stent implant in a large international cohort, both stand-alone and combined with cataract surgery. The study also examined secondary outcomes, including IOP changes, medication changes, adverse events (including specific types of hypotony and VA loss), and secondary glaucoma procedures.
Bambo et al, 2024	Y - The study acknowledges that while many studies have been published on the XEN45 implant, there is limited data on its long-term clinical outcomes. Cites pivotal studies within introduction.	Y - Yes, the study states specific objectives, with a primary focus on evaluating the long-term effectiveness and safety of the XEN45 implant. The study aims to assess both the standalone use of the XEN45 implant and its use in combination with cataract surgery
Clement et al 2020	Y - The study references prior research, including randomised controlled trials and multi-surgeon trials of trabecular micro-bypass stents	Y - The primary objective of the study is to evaluate the 2-year effectiveness and safety of iStent inject implantation with phacoemulsification. This is clearly stated in the abstract and further detailed in the "Purpose" section.
Dang et al 2018	PY - The introduction provides a general overview of glaucoma, its treatment, and the use of the Trabectome procedure.<br>The study specifically references earlier studies on the safety and efficacy of Trabectome, including results of their 12-month study in Chinese patients as well as global studies and meta-analyses. However, the review was limited with no citations of previous IDEAL stage principled studies.	Y - To evaluate the long-term (2-year) safety and efficacy of the Trabectome in Chinese glaucoma patients. This is focused on the effectiveness of the procedure in reducing IOP. The secondary objectives included assessing reductions in glaucoma medication, the 2-year success percentages, and surgical complications.
Deitz et al 2023	Y - The introduction cites prior studies to establish the context for the research and highlights the widespread use of iStent and ECP. It also cites studies that show the efficacy of iStent and ECP when combined with cataract surgery. There is no specific mention of earlier IDEAL stage reports.	Y - The primary objective is to compare the outcomes of phacoemulsification with ECP (phaco/ECP), phacoemulsification with iStent (phaco/iStent), and phacoemulsification with iStent and ECP (phaco/iStent/ECP) at up to 2 years of follow-up. It seeks to evaluate the safety of these procedures by recording complications and the need for additional surgery. The study aims to assess the effectiveness of these procedures by measuring changes in IOP and the reliance on glaucoma medication.
Ferguson et al 2020	PY - The study reviews existing scientific literature. It references a previous publication from the same group, which reported results up to 24 months. There is no explicit mention of IDEAL stage report demonstrating principles of stage 3 IDEAL framework.	Y - its objective to "investigate the long-term safety and efficacy of the trabecular microbypass stent placed in combination with cataract surgery in patients with POAG". It also aims to provide "real-world long-term data". The study also addresses the long-term safety of the procedure.
Gabbay et al 2021	Y - Yes, the study reviews existing scientific literature. It references prior studies on the XEN45 gel stent, including a two-year retrospective analysis by the same group,	Y - Yes, specific objectives are stated. The aim of the study is to extend the analysis of the XEN45 to 36 months. The study includes evaluating the efficacy and safety of the

	and other studies of similar devices. However, there is no specific mention of earlier IDEAL stage reports.	XEN45 gel stent over a longer period. This helps in recognising late or uncommon safety outcomes, and provides additional information about the use of the device.
Hengerer et al 2022	Y - The study references its own previous 3-year findings, which were published previously. Although the study does not explicitly refer to these previous publications as IDEAL stage reports, it does include prior reports on the same cohort of patients, which is in line with the intent of staged evaluation	Y - Evaluating the 5-year effectiveness and safety of iStent inject with or without cataract surgery in patients with open-angle glaucoma (OAG). Assessing intraocular pressure (IOP), medications, and indicators of disease stability (visual fields, retinal nerve fiber layer thickness, and cup:disc ratio) through 5 years.
Holmes et al 2022	Y - Cites previous clinical trials of the Hydrus Microstent and iStent inject, including their respective FDA pivotal trials. There is no reference to IDEAl stage specifically.	N - A general statement of analysing the real world effectiveness of the two devices is made. However a research question is not derived and specific objectives stated to detail how this will be done.
Inatani et al 2022	PY - The introduction cites previous studies that have evaluated the iStent, and mentions the limitations of the existing data for the Japanese population, thus justifying the need for the current study. The discussion also refers to other studies, some of which are also from Japan, when comparing the results. There is limited discussion of international studies and any earlier IDEAL stage-style reports	Y - To measure changes in IOP and medication use from preoperative values.<br>To evaluate the proportion of eyes that achieved treatment success at 6, 12, and 24 months postoperatively, in eyes with OAG implanted with iStent combined with phacoemulsification.<br>The study also evaluates safety outcomes, including perioperative or postoperative adverse events, stent malfunctions, and secondary glaucoma procedures. This objective allows for the identification of late or uncommon safety outcomes.
Katz et al 2018	PY - While the study comprehensively reviews the existing literature and references prior clinical and laboratory studies, it does not make any specific references to the IDEAL framework or its stages	Y - The primary objective of the study is clearly stated as evaluating the "long-term outcomes after one, two, or three trabecular micro-bypass stents implanted in a standalone procedure in eyes with open-angle glaucoma taking ocular hypotensive medication"
Lee et al 2020	Y - The introduction reviews existing literature regarding the evolution of surgical management of open angle glaucoma. It mentions the progression from Scheie procedures to trabeculectomy and glaucoma drainage devices, and then to microstents. There Is no reference to IDEAL stages but the review of literature mirrors this process.	Y - The study states its objective is to "investigate the early postoperative and long-term effects of two MIGS devices iStent and Hydrus in combination with phacoemulsification in terms of change of intraocular pressure (IOP) and number of glaucoma medications."
Mitchell et al 2024	Y - The study does review existing scientific literature. It references previous studies on: the effectiveness of selective laser trabeculoplasty (LTP) for initial IOP control; increasing use of LTP before surgical options like MIGS; studies examining MIGS outcomes in the setting of prior LTP; the effectiveness of ab-MIGS and phacoemulsification	Y - The main objective is to understand the long-term effectiveness of angle-based MIGS (ab-MIGS) procedures among eyes with and without prior LTP. The study also aims to identify factors associated with the need for subsequent glaucoma surgery after ab-MIGS. It seeks to provide information that has implications for understanding who may benefit most from ab-MIGS and for guiding patient and surgeon treatment expectations.The study also aims to understand the effectiveness of MIGS for those who have undergone prior LTP
Montesano et al 2023	Y - The study reviews existing literature on glaucoma treatments, including the effectiveness of incisional surgery, medical management with eye drops, laser trabeculoplasty, and cataract surgery. It also references studies on minimally invasive glaucoma surgery (MIGS) devices, specifically the Hydrus microstent. The study cites the previous HORIZON trial publications. However, the sources do not indicate that these are earlier IDEAL stage reports.	Y - The study's primary objective is to determine if cataract surgery combined with Hydrus microstent implantation (CS-HMS) reduces the rate of visual field worsening compared with cataract surgery (CS) alone in patients with primary open angle glaucoma (POAG). The study also examines:<br>The effect of intraocular pressure (IOP) on visual field progression; localized progression by analyzing different regions of the visual field; the probability of global visual field sensitivity dropping by predefined cutoffs from baseline; the time to progression of visual field damage; the proportion of fast progressors in each group; the study is focused on effectiveness, rather than safety outcomes.
Oberfeld et al 2023	PY - The introduction of the study notes that there is a growing interest in establishing whether combining multiple microinvasive glaucoma surgeries (MIGS) leads to an additive or synergistic effect. It also mentions that a number of case reports, retrospective studies and systematic reviews have reported on combined	Y - The stated objectives of the study are:<br>To compare the efficacy of the combined MIGS (cMIGS) procedure, Phacoemulsification, Endocyclophotocoagulation, and Kahook dual blade (PEcK), relative to its constituent single MIGS (sMIGS) procedures,

	MIGS (cMIGS). However does not mention any pivotal RCTs for Stage 3 of the IDEAL framework.	Phacoemulsification/Endocyclophotocoagulation (Phaco/ECP) and Phacoemulsification/Kahook dual blade (Phaco/KDB)
Oberfeld, Helwe et al 2023	Y - The introduction reviews existing literature on micro-invasive glaucoma surgery (MIGS), including studies on Hydrus microstent and Kahook Dual Blade procedures. The study also identifies the lack of comparative studies, which is a motivation for the present research. The study does not explicitly referency any previous IDEAL stage style reports which would be helpful to have a better overview of current literature.	Y - The study states the primary objective is to report the relative efficacy of combining phacoemulsification with either a Schlemm's canal microstent (Phaco/Hydrus) or dual blade trabecular excision (Phaco/KDB). This includes evaluating changes in intraocular pressure (IOP) and medication burden. The study aims to evaluate surgical outcomes of Phaco/Hydrus compared to Phaco/KDB, using Kaplan-Meier survival estimates and Generalized Estimating Equations.
Reitsamer et al 2022	Y - The study reviews existing scientific literature. It references previous studies on the effectiveness and safety of the gel stent at 2 years (single- and multicentre studies) and 3 years (single-centre study), but it does not refer to earlier IDEAL stage reports.	Y - The study states its primary objective as assessing the 3-year effectiveness and safety of the XEN gel stent. This includes:<br>Evaluating changes in mean IOP and IOP-lowering medication count from baseline; Recording intraoperative complications, adverse events of special interest (AESIs) and secondary surgical interventions (SSIs); Secondary objectives included assessing the same measures at 1, 2 and 4 years post-implantation.The study also assesses the proportions of eyes achieving specific IOP reduction levels and success rates, both complete and qualified
Salimi et al 2021	Y - The introduction cites previous studies on glaucoma prevalence, treatments, and the emergence of microinvasive glaucoma surgery (MIGS). It references the first FDA-approved MIGS device and the iStent inject. The introduction reviews prior evidence on the effectiveness of iStent and iStent inject. The study also refers to a previous one-year outcome report from the same cohort.	Y - The main objective is to assess the 3-year effectiveness and safety of iStent inject implantation with cataract surgery in eyes with different types and severities of glaucoma. The study aimed to provide valuable long-term real-world information to guide treatment decisions. While the primary focus is on effectiveness and safety outcomes, the study design also inherently allows for the identification of late or uncommon safety outcomes by tracking adverse events over a three-year period
Samuelson et al 2019	Y - The study reviews existing scientific literature by referencing previous studies on the iStent and iStent inject devices. It also refers to the existing literature about the use of MIGS procedures for glaucoma and prior landmark glaucoma trials that demonstrated reduced risk of visual field decline with IOP reduction. While it refers to prior studies, the current study does not explicitly reference earlier IDEAL stage reports. The study does cite publications regarding earlier generations of the iStent device and prior studies of the iStent inject.	Y - The study's objectives are clearly stated. The primary objective was to assess the effectiveness of the iStent inject by measuring a greater than 20% reduction in unmedicated IOP at 24 months. Secondary objectives included assessing changes in unmedicated IOP from baseline and also safety measures. Although not explicitly stated as an objective, the study does address late safety outcomes as it followed participants for two years. The study design does not address risk adjustment or quality assurance specifically, but safety outcomes were monitored throughout the 2-year follow-up
Samuelson et al 2021	Y - The study reviews existing literature on quality of life (QOL) in glaucoma and cataract patients. It discusses how QOL is impacted by these conditions and their treatments. It references studies that have not shown significant QOL differences between glaucoma treatments. It also cites studies on the negative impact of ocular surface disease (OSD) on QOL in glaucoma patients. There is no mention of IDEAL stages in the context of previous publications.	Y - The study's primary objective, as stated in the abstract, is to "assess quality of life (QOL) as measured by patient-reported outcomes (PRO) within the iStent inject ® pivotal trial". The study also states that they are conducting a "post hoc exploratory analysis". The study also aimed to investigate "the comparative QOL changes of cataract surgery plus implantation of the trabecular micro-bypass stent (iStent inject ®) vs cataract surgery alone". While the study focuses on effectiveness by looking at QOL improvements, it does not explicitly aim to identify late or uncommon safety outcomes, or changes in the use of the procedure/device, risk adjustment, or quality assurance
Schlenker et al 2017	PY - The study does review existing scientific literature, referencing previous studies on trabeculectomy outcomes and risk factors. It also references studies on the Xen 45 microstent but does not delve deeper into any which would reference the principles of previous IDEAL stages.	Y - The study does state specific objectives, with a primary focus on comparing the effectiveness of the two surgical procedures. These objectives include: Evaluating the hazard ratio (HR) of surgical failure in patients undergoing trabeculectomy versus microstent surgery; Examining secondary outcome measures such as IOP thresholds, medication use, best-corrected visual acuity, postoperative interventions, and complications; Identifying risk factors for surgical failure, such as ethnicity and diabetes; Assessing the safety profiles of both procedures by recording complications and reoperations

Strzalkowska et al 2021	PY - Introduction cites several publications regarding trabeculectomy (TRAB) and ab interno trabeculectomy (AIT). Mentions one RCT that compared phaco-AIT to phaco-TRAB but stopped due to lack of clinical equipoise. Does not specifically reference any IDEAL stage 3 pivotal RCTs.	N - States that primary aim of study would be to analyse further evidence of AIT vs Trabeculectomy but does not specify which objectives would be achieved
Tojo et al 2020	Y - The study reviews existing scientific literature. It references prior research on trabectome surgical outcomes, noting reported mean postoperative IOP values.It acknowledges conflicting findings regarding the suitability of different preoperative IOP values for trabectome surgery, which serves as the rationale for the study. It mentions previous reports on factors influencing surgical outcomes, such as thin central corneal thickness (CCT), simultaneous cataract surgery, and history of selective laser trabeculoplasty (SLT). The study does not explicitly reference earlier IDEAL stage reports in previous publications. However, the study cites multiple publications on trabectome surgery and outcomes	Y - The primary objective of the study was to compare surgical outcomes of trabectome surgery in patients with different preoperative IOP values. It also aimed to investigate factors affecting surgical outcomes including age, central corneal thickness (CCT), history of selective laser trabeculoplasty (SLT), and simultaneous cataract surgery. The study seeks to determine which preoperative IOP values are appropriate for trabectome surgery. The study does not explicitly state objectives related to identifying late or uncommon safety outcomes, identifying changes in the use of procedure/device, risk adjustment, or quality assurance.
Vukmirovic et al 2023	PY - the study reviews existing scientific literature, including references to trabeculectomy which is a similar procedure, and makes reference to other studies that have examined the outcomes of glaucoma surgeries. However, does not provide enough information about studies in relation to previous IDEAL stages for the Xen-45 Gel stent.	Y - the study states specific objectives, which include: Determining whether known risk factors for trabeculectomy failure similarly influence gelatin stent outcomes; Identifying surgical factors that may optimise success; Examining the effect of key baseline factors on gelatin stent survival with regards to IOP reduction across a medium-term follow up period of 24 months; Analyzing secondary endpoints including number of postoperative glaucoma medications, rate of needling with 5-fluorouracil (5-FU) and postoperative complications.
Yang et al 2023	Y - The study references prior research on the efficacy of MIGS procedures, including studies on ECP and goniotomy or trabeculotomy, noting that many of these are retrospective series without control groups and do not account for the IOP-lowering effect of phacoemulsification. The study does not explicitly reference earlier IDEAL stage reports but references RCTs.	Y - States objectives to evaluate effectives of MIGS by investigating rate of reoperation, IOP reduction, visual acuity (VA) changes, postoperative complications based on diagnosis coding within 90 days and patient factors predictive of surgical success for MIGS procedures.
