# Supplementary material for: Evaluating Long-Term Effectiveness and Safety of Glaucoma Surgical Innovations: A Systematic Review
Source: J Glaucoma. 2026 Oct 16;35(11):838–45. doi: 10.1097/IJG.0000000000002770 (PMC13722426; doi:10.1097/IJG.0000000000002770)
Supplement: Supplementary file 4 [file ijg-35-838-s004.pdf]

Supplementary Table 4. Data extraction as per IDEAL Stage 4 Framework for Methods (rated Yes, Partially Yes or No)

	**Methods ie. Design, data source, participants, intervention, outcomes, statistical methods**							
**Authors + Year**	**3. Does the methodology describe the study design (e.g. registry, analysis of real-world data, etc)? If yes, what is it? (Y, PY, N)**	**4a. Is there a description of the dataset, including who designed and funds it and who curates and manages it, addressing possible conflicts of interest? Was the dataset purposely designed for research or collected as a routine database? (Y, PY, N)**	**4b. Are structured data fields described or referenced and each item defined, included as an appendix or supplement when necessary? (Y, PY, N)**	**5. Is there a detailed account of inclusion and exclusion criteria for subjects? (Y, PY, N)**	**6. Is there a description or reference to the intervention/device being monitored, and comparator if applicable? (Y, PY, N)**	**7. Is there a description of pre-specified primary and secondary outcome measures selected and how they will be assessed, including patient reported outcome measures, when appropriate, utilising those measures that are standardised and validated, when available and applicable? (Y, PY, N)**	**8a. Were statistical methods used to describe and assess patient characteristics and outcomes, including methods of additional analysis (e.g. pre-specified subgroup analysis), and methods to minimize confounding, where appropriate? (Y, PY, N)**	**8b. Was there an explanation for how missing data was addressed? (Y, PY, N)**
Ahmed et al 2022	Y - the methodology clearly describes the study design. It is a prospective, multicentre, single-masked, randomised controlled clinical trial conducted at 38 investigational centres worldwide	Y - The dataset was purposely designed for research. The study was designed by Ahmed, Samuelson, and Singh.The study was funded by Ivantis, Inc. The study was conducted at multiple investigational centres. The data was managed by an independent reading centre masked to the treatment assignment.	Y - The study describes various data fields and outcome measures. These include: Intraocular pressure (IOP), glaucoma medication use, repeat glaucoma surgery, visual acuity, visual field, procedure-related adverse events, and corneal endothelial cell counts. Definitions for some terms are provided within the text, such as how a repeat glaucoma surgery is defined. Additional data, such as baseline participant demographics and characteristics, can be found in the supplemental tables	Y - Inclusion criteria included: age-related cataract, mild to moderate primary open-angle glaucoma, receiving 1 to 4 topical hypotensive medications, ophthalmoscopically detectable glaucomatous optic neuropathy, mild to moderate visual field loss, best-corrected visual acuity of 20/40 or worse, Shaffer grade III or IV angle width, and a medicated IOP of 31 mmHg or less. After a medication washout, a mean diurnal IOP between 22 and 34 mmHg was required. Exclusion criteria included: angle-closure glaucoma, any secondary glaucoma, a visual field mean deviation worse than -12 decibels (dB), exudative age-related macular degeneration, proliferative diabetic retinopathy, significant risk of glaucomatous progression due to medication washout, a narrow anterior chamber angle (Shaffer grade I or II), central corneal thickness of < 480 mm or > 620 mm, prior corneal surgery, cycloablation, or any incisional glaucoma procedure (except prior SLT), and prior argon laser trabeculoplasty	PY - the study describes the intervention and comparator. The intervention is the implantation of a Hydrus Microstent (HMS) in conjunction with cataract surgery (CS). The Hydrus Microstent is an intracanalicular microstent but study does not describe surgical methodology. The comparator is cataract surgery alone (CS)	Y - Primary outcome measures included: Intraocular pressure (IOP), glaucoma medication use, repeat glaucoma surgery. Secondary outcome measures included: visual acuity, visual field, procedure-related adverse events, and corneal endothelial cell counts. These outcomes were assessed using standard methods: IOP was measured using Goldmann applanation tonometry, visual acuity was assessed using BCVA measurement, visual fields were evaluated with perimetry testing, and endothelial cell density was measured using specular microscopy	Y - Yes, statistical methods were used to describe and assess patient characteristics and outcomes. Continuous variables were analysed using means and standard deviations, and between- and within-group differences were tested with 2-sample t-tests. Categorical variables were compared using the Fisher exact test. Hazard ratios were calculated using the Cox proportional hazards model. Kaplan-Meier survival analysis was used to assess cumulative rates of failure and reoperation for glaucoma. Linear regression analysis was used to assess long-term endothelial cell count changes. Pre-specified subgroup analysis was conducted for eyes with different preoperative medication counts. The study used randomisation to minimise confounding, and the study groups were well-matched for preoperative IOP, medication use, washed-out DIOP, and glaucoma severity	N - The study does not explicitly describe how missing data were handled for participants who did not complete the full 5-year follow-up. The study states that five-year follow-up was completed for 83.4% of participants in the HMS group and 71.7% in the CS group
Ahmed et al 2023	Y - the study is a 5 year follow-up safety study of a previously conducted	Y - Yes, there is a description of the dataset, including who	Y- Structured data fields are described and defined within the main text of the source document,	Y - Study describes inclusion and exclusion criteria for both the 2 year trial and 5 year follow up study.	Y - There is a a description of the iStent inject and the	Y - The study's primary outcome measure was the overall safety endpoint,	Y - Use of descriptive statistics, inferential statistics, used methology to reduce	PY - The study details patient enrolment, reasons for non-participation, and methods to

	prospective randomised, single-masked, concurrently controlled, multicentre iStent inject pivotal trial	designed and funds it (Glaukos Corp), and addressing possible conflicts of interest. The dataset was purposely designed for research.	they are not presented as a separate appendix or supplement		comparator is phacoemulsification alone.	defined as the incidence of clinically relevant complications associated with iStent inject placement and stability through the 60-month follow-up. The study's secondary outcome measures were related to corneal endothelial safety.	confounding variables eg. masked assessment of corneal endothelial cells. The study did not explicitly describe any pre-specified subgroup analyses. The use of the linear model with treatment-by-month interaction allowed for assessment of how the treatment effect varied over time	assess selection bias. However, it does not provide a detailed explanation of how missing data within the longitudinal follow-up was specifically handled in the statistical analysis
Arnould et al 2024	Y - The study is a retrospective analysis of prospectively collected data from the Fight Glaucoma Blindness (FGB) registry. The FGB registry is an international, web-based platform that collects real-world data from routine clinical practice.	Y - The FGB registry is part of the Save Sight Registries group in the University of Sydney. Funding was supported by the Royal Australian and New Zealand College of Ophthalmologists. The registry is a web-based platform where specialists enter data from their routine clinical practice. It is part of the Save Sight Registries group. The authors of the study were involved in the planning of the study and the data acquisition, analysis and interpretation of results.The data was collected as a routine database, designed for auditing and research of real-world glaucoma treatments.	PY - The study mentions that the FGB registry requires a baseline visit with a minimum dataset that accurately characterizes patients, along with pertinent clinical measurements such as visual acuity (VA), intraocular pressure (IOP), and previous surgical procedures. It also states that each follow-up visit includes mandatory fields such as VA, IOP, medical treatments, adverse events and additional procedures. However, the specific data fields and their definitions are not detailed in the main text of the article, though they are referenced as having been described previously in a publication about the registry	PY - Inclusion criteria: Eyes with a diagnosis of open-angle glaucoma or ocular hypertension (however the difference between these is not detailed); Patients with a follow-up of at least 6 months. Exclusion criteria: Eyes with angle closure spectrum disease; Eyes with prior glaucoma filtration surgery; Eyes that had IOP measured with a technique other than Goldmann; Patients under 18 years old.	PY - Intervention/Device: The study focuses on the Xen 45 gel stent implant, a bleb-forming device used as an alternative to traditional filtration surgery for glaucoma. Comparator: The study does not include a direct comparator group in terms of a different device or intervention. However, it does compare outcomes between patients who had the Xen 45 gel stent implant as a standalone procedure and those who had it combined with cataract surgery. The surigical technique was not described.	Y - Primary Outcome: Success at 24 months, defined as IOP ≤18 mm Hg, ≥6 mm Hg and ≥20% reduction from baseline with no secondary procedures. The outcomes are assessed using the data collected in the registry at specific follow-up time points (6, 12, 24, 36 and 48 months). The study uses World Glaucoma Association guidelines for defining success	Y - T-tests were used for continuous variables and chi-squared tests for categorical variables to compare baseline characteristics between the Xen and Xen+cataract groups.Modified linear models were used to compare outcomes between the Xen stand-alone and Xen+cataract groups, adjusting for gender, age, preoperative IOP and preoperative number of medications. Linear mixed models were used to account for clustering of data. The study conducted a pre-specified subgroup analysis to compare outcomes between Xen stand-alone and Xen+cataract groups.Kaplan-Meier estimation was used to plot the probability curves of qualified and complete success. Cox proportional hazard models were used to estimate crude and adjusted hazard ratios (HR) of failure, adjusting for confounders.	PY - The study used a "last observation carried forward" (LOCF) analysis. This means that for patients who had a secondary glaucoma procedure, the IOP and medication values immediately prior to the secondary intervention were carried forward to the relevant follow-up time window. Bleb needling was not included as a secondary glaucoma procedure, and therefore did not censor the follow-up
Bambo et al, 2024	Y - The study is a retrospective and single-centre study conducted on consecutive patients who underwent a XEN45	PY - In summary, the study provides some information about the dataset, including its source. The study	Y - While the study provides detailed descriptions of these data fields within the text and tables, it does not include a separate appendix or supplement with	Y - The study clearly outlines the specific conditions and situations that led to a patient being included or excluded from the study.	Y - The study provides a clear description of the XEN45 gel stent as the intervention, and it uses a comparison between XEN45 implant alone and	Y - The study clearly defined its primary outcome measure as the reduction in IOP and the secondary measures as	Y - The study employed a range of statistical methods to describe and assess patient characteristics and outcomes. It also implemented measures	Y - The last-observation-carried-forward (LOCF) method was used to impute missing data. This method involves carrying the last observed

	implant, either alone or in combination with cataract surgery, between November 2016 and October 2021	indicates that the dataset was collected retrospectively specifically for this research. However, the study does not explicitly identify who specifically curated or managed the dataset.	explicit definitions of each item. Instead, the definitions are embedded within the methodology and results sections. For example, surgical success is explicitly defined in the methods section		combined with phacoemulsification as a comparator to determine whether there are differences in the outcomes between the two groups	mean IOP at the last follow up visit, reduction in medication use, the proportion of eyes achieving target IOPs, and the incidence of adverse events. The study used standard ophthalmological assessments and statistical methods and provided specific definitions and methods of assessment for the outcome measures.	such as subgroup analysis and covariate adjustment to account for potential confounders	value forward to replace any missing values in the dataset
Clement et al 2020	Y - The methodology describes the study design as a retrospective study that involved the analysis of real-world data.<br>Specifically, the study is a retrospective outcome assessment of pooled data from nine surgeons across Australia. The researchers reviewed records to identify eyes that had been implanted with the iStent inject in the setting of cataract surgery between January 2016 and February 2018	Y - The dataset was not designed for research but consists of de-identified clinical data collected from routine patient care. The study was not financially supported, however, some authors had financial relationships with the manufacturer of the device being studied, presenting a potential conflict of interest.	Y - Yes, structured data fields are described in the study, though they are not included as an appendix or supplement. The study defines the data points that were collected and analysed, but these are presented within the main body of the article	Y - The inclusion criteria ensured that all participants had both a cataract requiring surgery and a need for additional glaucoma management, and the study sought to include a variety of glaucoma types. The exclusion criteria were designed to avoid confounding factors, such as other ocular diseases or conditions that might affect the results.	Y - The study provides a detailed description of the iStent inject device and its implantation technique. While there isn't a direct control group receiving cataract surgery alone, the study does compare outcomes to the preoperative baseline, using other studies as references for comparison	Y - The study used a combination of pre-specified, standardised clinical measures (IOP, medication use, visual acuity, CDR, and visual fields) as primary and secondary outcomes. These measures were assessed using standard clinical methods and statistical analyses.	Y - The study employed various statistical methods, including descriptive and inferential statistics, to analyse patient data and assess outcomes. It also incorporated pre-specified subgroup analyses to evaluate the effectiveness of iStent inject across different glaucoma subtypes. The use of inclusion and exclusion criteria and the consideration of other comparative trials helped to minimise potential confounding, though there are limitations associated with its retrospective design and lack of a formal control group	N - While the study acknowledges and addresses missing data by reporting the number of eyes with available data for each measurement, it does not describe any specific methods for handling missing dat
Dang et al 2018	Y - the methodology clearly describes the study design. It is a "multicenter, retrospective, observational study". Researchers collected and analyzed data from past cases at multiple hospitals, rather than prospectively enrolling patients in a trial.	PY - The study provides some information regarding the dataset but does not explicitly state who designed and curates the dataset, or whether it was purposely designed for research or collected as a routine database. The data comes from five glaucoma centers. The study received financial support from the National Natural Science Foundation of China. It is not explicitly stated if the data was collected specifically for the study or if it was extracted from	Y- The study describes the demographic data such as age, gender, glaucoma diagnosis, preoperative visual acuity, visual field status, disc cup/disc ratio, lens status, Shaffer grade, and prior surgeries. The intraocular pressure (IOP) was measured using Goldmann applanation tonometry.<br>Visual field was categorized as mild, moderate, or advanced according to the Humphrey visual field test. The number of glaucoma medications used were also recorded	Y - Inclusion criteria: Patients with primary or various secondary open-angle glaucomas and narrow angles were included. Exclusion criteria: Patients with neovascular glaucoma and those with post-operative follow-ups of less than 3 months were excluded	Y - The study focuses on the Trabectome (NeoMedix, Tustin, CA, USA), a minimally invasive glaucoma surgery device that removes the trabecular meshwork (TM) using a high-frequency bipolar electrode. The surgical procedure using the Trabectome is described, including the incision and ablation technique. The study also includes a comparison between patients who received Trabectome alone and those who received Trabectome combined with phacoemulsification and intraocular lens implantation	Y - The primary outcome was the reduction in intraocular pressure (IOP), measured using Goldmann applanation tonometry. Secondary outcomes included the reduction in the number of glaucoma medications, 2-year success percentages, and surgical complications	Y - Quantitative data was presented as the mean ± standard error (SE).The Wilcoxon test was used to compare postoperative IOP and the number of glaucoma medications with baseline values. The Kaplan–Meier test was used to calculate the 2-year success percentage. Logistic regression was used to determine risk factors related to Trabectome failure. The study also includes pre-specified subgroup analysis such as comparing the outcomes of combined surgery versus Trabectome alone and outcomes based on prior surgeries.	N - The study does not explicitly explain how missing data was addressed. The study does report on the number of 'NR' (not recorded) values for various measures including visual acuity, visual field, disc cup/disc ratio, and lens status. However, it is unclear how these missing data points were handled in the statistical analysis.

		a routine database; however, given the retrospective design, it's likely that the data was collected as part of routine clinical care and then compiled for this study.							
Deitz et al 2023	Y - Yes, the methodology describes the study design as a retrospective chart review. This means the researchers examined existing patient data collected from medical records. This is a type of observational study that analyses real-world data.	Y - The dataset consists of patient charts from the University of Colorado Department of Ophthalmology.The study was supported by a grant to the Department of Ophthalmology from Research to Prevent Blindness, Inc., the Frederic C. Hamilton Macular Degeneration Center and the Colorado Clinical & Translational Sciences Institute (CCSTI). The authors state that the funding organisations had no role in the design or conduct of this research. The data was not purposely designed for research, but rather collected as a routine part of patient care. This suggests the data comes from a routine database of patient records.	Y - The study does describe the data fields that were collected. These include: patient demographics, subtype and severity of glaucoma, prior glaucoma surgical treatment, number of IOP-lowering medications, best corrected visual acuity (BCVA), and intraocular pressure (IOP). The definition of glaucoma severity was determined according to the International Classification of Disease Tenth Revision (ICD-10) Glaucoma Stage Definitions. The study also defined an IOP spike as IOP ≥30 and/or a change in IOP of ≥10 from baseline.	PY - Inclusion criteria: Patients included in the analysis were over 18 years old, requiring surgery for visually significant cataract in addition to better IOP control and/or a reduction in topical glaucoma treatment, and who underwent uncomplicated phaco/ECP, phaco/iStent, or phaco/iStent/ECP. Exclusion criteria: Patients with neovascular glaucoma, chronic angle closure glaucoma, missing pre-operative IOP data, any additional surgeries at the time of the procedure, or without at least 6 months of follow-up, were excluded from the analysis	Y - The interventions include:phacoemulsification combined with endoscopic cyclophotocoagulation (phaco/ECP), where ECP involves laser treatment of the ciliary body to reduce aqueous production; phacoemulsification combined with iStent trabecular microbypass implantation (phaco/iStent), where the iStent is a titanium implant that facilitates aqueous outflow; phacoemulsification combined with both iStent and ECP (phaco/iStent/ECP). Surgical technique is described.	Y - Primary outcome measures are IOP and the number of IOP-lowering medications. Secondary outcome measures include best corrected visual acuity (BCVA), and surgical complications. IOP was measured in mmHg. BCVA was assessed using a Snellen chart. Complications and the need for additional surgery were also recorded. The study defines success as an IOP reduction of at least 20% and/or a reduction of at least one glaucoma medication. The World Glaucoma Association’s guidelines were followed in recording BCVA, IOP, glaucoma medication use, and complications	Y - Logistic regressions with general estimating equations were used to compare eye characteristics between the three study groups, accounting for intrasubject correlation. Linear regression with generalised estimating equations was used to model outcomes of IOP and the number of medications, also accounting for correlation of multiple visits and potentially two eyes included. Means are reported with standard errors. P-values less than 0.05 were considered statistically significant.The study performed a pre-specified subgroup analysis by excluding severe glaucoma cases to assess if the difference in success rates between the groups changed	N - The study does not provide an explicit explanation for how missing data was addressed. However, the study notes that patients with missing pre-operative IOP data or without at least 6 months of follow-up were excluded from the analysis.
Ferguson et al 2020	Y - The methodology describes the study design as a "retrospective case series". The study was designed to "mimic typical clinical use of the device and provide real-world long-term data"	PY - The study used data from a retrospective chart review of consecutive patients who underwent the procedure. The data was collected from procedures that had already occurred. The dataset was not designed for research, but was collected as a routine clinical dataset. The study was funded by an investigator-initiated trial grant from Glaukos Corp. We don't know who	PY - Study described structured data fields (IOP, number of glaucoma medications, glaucoma severity, demographic data, visual acuity, need for additional surgery and incidence of IOP spikes. No supplement or appendix available.	PY - Inclusion criteria was all consecutive patients who had a single single trabecular microbypass stent implanted in combination with cataract surgery, All patients included had a preoperative diagnosis of mild to severe primary open-angle glaucoma (POAG). No exclusion criteria was defined.	Y - the intervention being monitored is a single trabecular microbypass stent (iStent) in combination with phacoemulsification (cataract surgery). The study does not use a comparator group, but compares outcomes to baseline data before the intervention. The study mentions that traditional surgical filtering procedures like trabeculectomies and tube shunts are alternatives but are not directly compared.	Y - Primary outcome measures were mean IOP, mean number of glucoma medications, secondary outcomes were safety including OP spikes above baseline, need for additional suergey, visual outcomes and proprotional/magnitudinal reduction in IOP	Y - A parametric paired t-test procedure was used to determine the significance of the mean change in IOP and number of glaucoma medications from baseline to all time points 6 months postoperatively. The study also performed subgroup analyses based on baseline IOP, baseline number of medications, and glaucoma severity	N - Study acknowledges that missing data was present at time points of 1 day, 1 week, 1/3/6 months and yearly up to 6 months but not how it handled missing data at these intervals

		specifically managed and curated the dataset.						
Gabbay et al 2021	Y - The methodology describes the study design as a retrospective (historical prospective) non-comparative audit of medical records. The study was conducted at a single hospital trust.	PY - The data was collected from medical records at the Southend University NHS Foundation Trust. The audit was approved by The Southend University Hospital Audit Committee. The data was collected retrospectively on patients who had undergone XEN45 implantation. The data was not specifically designed for research, but collected as part of routine clinical care. The authors declare no potential conflicts of interest. There is no statement about who specifically curated or managed the dataset.	Y - The study describes the data fields collected: IOP measurements; Number of antihypertensive medications used; Patient demographic data, including age, gender, ethnicity; Glaucoma type; Previous cataract or glaucoma surgery; Type of XEN45 procedure (standalone or combined); Visual acuity (measured by Snellen chart); Complications and adverse events; Need for further surgical procedures or removal of the stent.<br>These data are presented in tables with clear definitions provided	Y - Inclusion: Patients who underwent antimetabolite-augmented XEN45 implantation for the treatment of glaucoma between June 2015 and May 2017 at Southend University NHS Foundation Trust. Exclusion: For patients who underwent the procedure in both eyes, only the first eye was included. Training cases for surgeons were excluded	Y - The XEN45 Gel Stent is described as a 6-mm cross-linked porcine-derived collagen tube with a 45-micron lumen. Also notes procedure used antimetabolite (mitomycin C). The study does not directly use a comparator group. However, there is some comparison to findings from other studies using different interventions such as trabeculectomy	Y - Primary Outcome Measures: IOP and number of antihypertensive medications used. These were measured at various time points, including pre-operative, post-operative and at intervals of 6 months up to 36 months. Secondary Outcome Measures: Success was defined as a 20% decrease in IOP without additional medications or a reduction in medication use without increased IOP. Failure was defined as the need for further surgical procedures or removal of the stent. Needling rates were also assessed. Visual acuity was also measured using a Snellen chart.	Y - One-way ANOVA with a Tukey post hoc test was used to compare mean values of IOP and the number of medications used between preoperative and postoperative timepoints. Multivariable Cox regression analysis was used to investigate the impact of preoperative factors on survival. Kaplan-Meier curves were used to estimate the probability of survival, with comparisons between groups using log rank (Mantel-Cox), Breslow (generalized Wilcoxon), and Tarone-Ware tests. Subgroup analysis was performed based on ethnicity and gender. The study aimed to minimize confounding by using multivariable regression analysis to assess the independent effect of several variables on survival	N - Data only available for 53 out of 205 patients at 36 month follow up. Study does not explicilty address how this missing data was imputated and/if any statistical methods were utilised.
Hengerer et al 2022	Y - The study is described as a prospective, longitudinal, single-surgeon study	Y - The dataset was designed and funded by Fritz H. Hengerer, who performed all surgeries and managed the patients, and by the Glaukos Corporation. They are available by contacting authors. The dataset was purposely designed for research to evaluate the outcomes of iStent inject.	Y - The study describes a number of structured data fields, including: demographics, preoperatiev characteristics like VF MD and intraoperative/postoperative measures.	Y - Inclusion criteria for the study: Diagnosis of glaucoma, including primary open-angle glaucoma, pseudoexfoliative glaucoma (PXG), combined-mechanism glaucoma, pigmentary glaucoma, or secondary (neovascular) glaucoma; History of inadequate response to prior surgical and medical glaucoma therapy; Need for cataract surgery for the Combined subgroup. Exclusion criteria for the study: Corneal opacities preventing gonioscopic visualization; Angle-closure glaucoma; Pregnancy; Congenital glaucoma; Age under 18 years; Active ocular inflammation.	Y - The study does not include a comparator group in the traditional sense (i.e., no control group receiving a different treatment). However, it does include a subgroup analysis comparing the combined and standalone procedures. There is a description of the surgical procedure.	PY - The study does not describe specifically whether the outcome measures were standardized or validated, but they are commonly used measures in glaucoma research.	Y - The study attempted to minimise confounding by including a single surgeon, and by using subgroup analysis to evaluate the independent effect of the stents versus cataract surgery. Inferential statitsics like paired t-tests and chi-square tests were used as required depending on the variable type.	N - The study notes that nearly all eyes (121/125 or 97%) completed 5 years of follow-up, indicating a very low level of missing data. The study does not provide a detailed explanation of how missing data was addressed, but with such a high completion rate, the impact of any missing data is likely to be minimal.
Holmes et al 2022	Y - Study is an observational study that uses real-world data from a clinical registry to compare outcomes of two different surgical procedures, using	PY - The dataset used in this study was from the FGB registry, which was designed as a clinical database for both audit and research, and was not specifically designed	Y - Structured data fields are described in the methodology of the study, although they are not included as an appendix or supplement	Y - Inclusion criteria and exclusion criteria defined appropriately	N - Study defines Hydrus and iStent as ab-interno procedures but does not expand on this within methodology	PY - Outcome measures are stated but how they will be assessed is not made clear within methodology. More information eg. Goldmann applanation for	Y - The study employed a range of statistical methods to account for differences in patient characteristics and potential confounders. These methods included regression models, propensity score	N - Missing data was not addressed

	statistical methods to adjust for differences in baseline characteristics	for research, it was collected as a routine database. The study discloses funding and confirms that the funders had no role in data acquisition or analysis, and states that there are no conflicts of interest. The registry is managed by the Save Sight Registries group at the University of Sydney.				IOP available throughout text.	matching and a 'last observation carried forward' analysis, and were applied to both the unmatched and matched cohorts.	
Inatani et al 2022	Y - The methodology clearly describes the study design. It is a "24-month, prospective, longitudinal, observational post-marketing surveillance (PMS) study".	PY - There is a description of the dataset, though not all details are fully fleshed out.The study was sponsored by Glaukos Corporation. The study was conducted as part of mandatory actions for the approval of iStent in Japan. This implies that the dataset was purposely designed for research and regulatory purposes, rather than collected as part of a routine database.The authors mention that the data was collected from 23 sites in Japan. The actual curation and management of the dataset is not explicitly described, although it can be assumed that the data analysis was conducted by the authors and their team.	Y - The data fields included: intraocular pressure (IOP), number of antiglaucoma medications, visual field mean deviation (VF MD), and central corneal endothelial cell density (ECD). These were collected preoperatively and at defined time points postoperatively.<br>The study also defines "treatment success" and "treatment failure".	PY - The inclusion criterion was "all eyes implanted with one iStent in conjunction with phacoemulsification". There are no specific exclusion criteria explicitly mentioned in the paper.	Y - there is a description of the intervention/device (iStent trabecular micro-bypass stent system). There is no comparator group in this study. The study compares pre- and post-operative data within the same group of patients.	Y - Primary outcome measures: These included changes in intraocular pressure (IOP) and the number of antiglaucoma medications from preoperative values. Secondary outcome measures: This includes the proportion of eyes that achieved treatment success at 6, 12, and 24 months postoperatively. Treatment success is defined in the methods section. The proportion of medication-free eyes and the proportion of eyes with IOP ≤ 15 mmHg and ≤ 18 mmHg at 24 months were also measured.	Y - The study used descriptive statistics (mean, standard deviation, median, interquartile range) to describe patient characteristics. Wilcoxon signed-rank test with Bonferroni correction was used to compare preoperative and postoperative outcomes of IOP and medications. Multilevel mixed models were used to account for repeated measures from the same eye and clustering of eyes by hospital. Kaplan–Meier survival estimates were used to determine the proportion of patients that achieved treatment success postoperatively. Pre-specified subgroup analysis was performed for glaucoma subtypes (POAG, NTG, XFG), preoperative IOP levels, and preoperative medication use. Multilevel mixed models were used to minimize confounding by accounting for the use of different or same tonometer for IOP measurements.	PY - The study explains that the reasons for missing data at POM24 were mainly due to non-clinical factors, such as not being present at the hospital or a change in address. The study used all available data at each time point for the analysis. No statistical models were used.
Katz et al 2018	Y - It is a prospective, randomised, open-label evaluation of the long-term safety and performance of one, two, or three iStent trabecular micro-bypass stents. A detailed breakdown is provided.	Y - The study was designed and sponsored by Glaukos Corporation. Data was collected at the S.V. Malayan Ophthalmological Center in Yerevan, Armenia, where the surgeries and follow-up visits were	PY - The study does describe structured data fields, but does not include them as an appendix or supplement. The structured data fields are referenced and defined within the methodology and results sections of the study.	PY - The study does reference that there are detailed inclusion and exclusion criteria for subjects, but these are not included in the text	Y - There is a description of the intervention/device being monitored, and a comparator is also implicitly present via the different treatment groups	Y - The study has pre-specified primary and secondary outcome measures which are clearly defined and are assessed using standard methods. The primary outcome measures focus on the reduction of IOP using	Y - Use of descriptive statistics, analysis of change over time, ANOVA, Kaplan Meier plots. The study does not explicitly mention any other specific methods used to control or minimize confounding or perform on subgroup analysis.	N - The study addressed missing data mainly by excluding data from subjects who underwent cataract surgery from efficacy analyses. The study also acknowledges subject attrition, and reports the number of subjects included in analysis at the 42-month follow-up. The study did not describe any imputation

		complete. The dataset was purposely designed for research to evaluate the long-term safety and performance of iStent trabecular micro-bypass stents. While it is stated that Glaukos provided data management, the document does not explicitly name the specific individuals or teams that curated and managed the dataset.				different numbers of iStents, and the secondary outcomes look at safety parameters and the need for additional medications, with visual field and visual acuity also being tracked.		techniques to account for missing data.
Lee et al 2020	Y - The methodology clearly states that the study is a "retrospective consecutive case series" conducted in a "subspecialty cataract and glaucoma private practice". It is also described as using "real world data"	PY - It does not explicitly state whether the dataset was purposely designed for research or collected for other purposes. This is a retrospective study, so it's reasonable to infer the data was collected during clinical practice and then used for research.	Y - The study mentions the data collected including, "age, gender, type of OAG, surgical operation undertaken, the pre- and postoperative IOP and number of medications up to two years post-surgery". The methods section describes how IOP was measured. The types of OAG are specified as "primary open-angle glaucoma, pseudoexfoliation glaucoma or pigmentary glaucoma"	Y - The study specifies that patients were included if they were undergoing phacoemulsification for cataract removal, and if they met the criteria for microstent insertion through having mild to moderate OAG (primary open-angle glaucoma, pseudoexfoliation glaucoma or pigmentary glaucoma) and did not require other glaucoma surgical management such as trabeculectomy or glaucoma drainage device	Y - The study clearly describes the interventions, which are phacoemulsification alone, and phacoemulsification combined with either the iStent or Hydrus MIGS devices. The devices are identified by brand name and manufacturer. The surgical technique for phacoemulsification and MIGS device insertion is described in detail	PY - The study states that the main outcome measures were intraocular pressure (IOP) and the number of medications. Outcome measures are objective clinical measures. However, IOP has been measured using both iCare and Goldmann Applanation Tonometry.	Y - The study explicitly mentions the use of linear mixed models to analyze changes in IOP and number of medications over time and between treatment groups. It notes that a "first-order autoregressive repeated covariance type was used which assumes that adjacent measurements from the same individual are more highly correlated than observations farther apart in time". The study also states that "P values of <0.05 were considered statistically significant and a Bonferroni correction was applied to adjust for multiple comparisons". The study also used Chi-Square analysis to examine frequencies.	PY - The study states that "For some patients, the 6 months data was not available, but were reviewed later at 12 and/or 24 months". It also acknowledges "6–15% missing data across the follow-up visits". However, it does not explicitly state how the missing data was addressed in the statistical analysis other than to note the sample size at each time point. The linear mixed models method they used, does allow for some missing data, but it is not discussed explicitly.
Mitchell et al 2024	Y - The methodology clearly describes the study design as a retrospective cohort study using data from the American Academy of Ophthalmology IRIS® Registry (Intelligent Research in Sight). This indicates that the study analyses real-world data	Y - The study mentions that the data was collected for the IRIS Registry from January 1, 2013 to December 31, 2018. It is stated that the data stored within the IRIS Registry is deidentified	Y - The study describes some structured data fields and provides definitions: Angle-based MIGS procedures;<br>Laser trabeculoplasty (LTP);<br>Glaucoma diagnoses; Glaucoma severity; Outcomes; Covariates; Demographic information (age, race/ethnicity) are described;<br>Baseline IOP: It was defined as the mean of all IOP measurements	Y - Inclusion: Eyes undergoing ab-MIGS (iStent, Hydrus, goniotomy, Trabectome, or ab internal canaloplasty) were included. Eyes that had undergone LTP within two years of the index ab-MIGS procedure were also included. Exclusion: Eyes that had undergone prior non-ab-MIGS procedures, prior traditional glaucoma surgery (trabeculectomy or GDD), or non-ab-MIGS or traditional	N - There is no description of the surgical methodology of the ab interno procedures being tested.	Y - The primary outcome measure was failure, defined as subsequent glaucoma reoperation after ab-MIGS. Reoperations included any standalone ab-MIGS procedure, non-angle-based MIGS, traditional glaucoma surgery (trabeculectomy or GDD), or any combination	Y - The study used descriptive statistics, group comparisons. time to event analysis, regression analysis, preopensity score matching, subgroup analysis	PY - The study notes that eyes with missing laterality or reoperation data were excluded from the analyses. However, there is no explicit explanation of how other types of missing data were handled. The authors also state that the size of the cohorts was limited to those with nonmissing outcome data

	collected within a registry setting.		obtained within 6 months prior to the index surgery.	glaucoma surgery concurrently with their ab-MIGS were excluded. Eyes with missing laterality or reoperation data were also excluded.		of these. The study also tracked intraocular pressure (IOP) trends over time		
Montesano et al 2023	Y - The methodology describes the study as a post hoc analysis of visual field data from the HORIZON trial, a prospective, multicenter, single-masked, randomized, controlled clinical trial.	Y - The dataset was collected as part of the HORIZON trial. This trial was a prospective, multicenter, single-masked, randomized controlled clinical trial. The trial was conducted at 38 centers (26 in the United States and 12 international). The study was funded by Ivantis, Inc., an Alcon company. The data consisted of visual field tests from participants with glaucoma and cataracts. The visual field tests were performed at baseline and 6, 12, 24, 36, 48, and 60 months after surgery. The data was collected using Humphrey field analyzers and anonymized printouts of the tests were provided by the individual centers.A bespoke optical character recognition algorithm extracted pointwise sensitivity values, which were then verified by two independent graders.	Y - The study describes the data fields used in its analysis. These are primarily derived from visual field (VF) tests, and include: Pointwise sensitivity values: Extracted from VF test printouts using an optical character recognition algorithm; Rate of progression (RoP): Calculated using a linear mixed model (LMM) to determine the change in VF sensitivity over time; Baseline sensitivity: The estimated sensitivity on the day of surgery; Intraocular pressure (IOP): Measured using Goldmann applanation tonometry. A time-weighted average IOP was calculated using linear interpolation; False positive error rate: Used to assess the reliability of the VF tests; Time from surgery: The time elapsed between the surgery and each VF test; Garway-Heath clusters: Used to analyze localized VF progression. The study also included data on patient demographics, such as age, sex, and race.	Y - Inclusion criteria: Age-related cataract and a diagnosis of mild to moderate primary open-angle glaucoma (POAG) using 1 to 4 topical hypotensive medications; Ophthalmoscopically detectable glaucomatous optic neuropathy; Mild to moderate VF loss according to Hodapp-Anderson-Parrish criteria; Best-corrected visual acuity of 20/40 or worse; Open iridocorneal angle; Medicated IOP of 31 mm Hg or less; Mean diurnal IOP (average of 3 Goldman tonometry measurements) between 22 and 34 mm Hg after washout of all hypotensive medications, with an increase of at least 3 mm Hg compared with the medicated IOP value. Exclusion criteria: Angle-closure or any secondary glaucoma (including pseudoexfoliative and pigment dispersion); Exudative age-related macular degeneration; Proliferative diabetic retinopathy; Significant risk of glaucomatous progression resulting from washout of IOP-lowering medications as judged by the local investigator; Narrow iridocorneal angle (Shaffer grade I or II) or any angle abnormality on gonioscopy; Central corneal thickness < 480 or > 620 mm; Clinically significant corneal dystrophy; Prior corneal surgery, cycloablation, and any incisional glaucoma procedure such as trabeculectomy, tube shunt implantation, deep sclerectomy, or canaloplasty; Patients who underwent prior selective laser trabeculoplasty, but not argon laser trabeculoplasty, were eligible.	PY - The study describes the intervention and comparator. The intervention is the implantation of a Hydrus Microstent (HMS) in conjunction with cataract surgery (CS). The Hydrus Microstent is an intracanalicular microstent but study does not describe surgical methodology. The comparator is cataract surgery alone (CS)	Y - Primary Outcome Measure: The difference in the rate of progression (RoP) of visual field (VF) damage between the CS-HMS and CS groups. This was measured using a linear mixed model (LMM). The study used standardized visual field testing using the Humphrey field analyzer.	Y - The study used a variety of statistical methods eg. Linear Mixed Models (LMM), sensitivity analysis, multivariable models, survival analysis, localised progression analysis, masking and randomisation	PY - The study acknowledged that a proportion of patients were lost to follow-up and that more patients were lost in the CS arm than the CS-HMS arm. Analysis was performed on all VFs from patients with at least 3 postoperative tests, the last performed at least one year after surgery. Eyes that were estimated to reach the event beyond their actual observation period were censored at the time of their last follow-up. A sensitivity analysis was performed including patients with at least two postoperative tests to assess the impact of the data selection.
Oberfeld et al 2023	Y - The study is a retrospective analysis of real-world data collected from patient records. The study used data from the surgical records at	PY - The dataset used in this study was a routine clinical database, not one designed for research. While the study does not provide specific details	Y - The study provides a clear description of the structured data fields used, including definitions for each item, in the text and within result tables	Y - The study provides a detailed account of the inclusion and exclusion criteria for subjects	Y - The study clearly describes the interventions being monitored (PEcK, Phaco/ECP, and Phaco/KDB), which are all surgical procedures. It also provides	Y - The primary outcomes were the changes in intraocular pressure (IOP) and medication burden over a period of up to 60 months postoperatively.	Y - The study employed robust statistical methods, including GEE, Kaplan-Meier survival curves, Log rank tests and Cox regression to assess patient characteristics and outcomes.	Y - The study provides an explanation of how missing data was addressed, primarily through the use of Generalised Estimating Equations (GEE) and by setting specific inclusion criteria

	Massachusetts Eye and Ear from January 2016 to July 2021. Groups were non-randomised.	on who curates and manages the data, it does acknowledge funding sources and potential conflicts of interest, although there may be limitations related to bias in the selection of data for analysis due to the study's retrospective nature			information about the devices used in those procedures and the specific surgical techniques applied	These outcomes were compared between the three procedures (PEcK, Phaco/ECP, and Phaco/KDB) secondary outcomes (visual acuity, procedure time, and postoperative complications), assessed using standardised and validated measures where available.	They also used statistical adjustment in their models to minimise confounding. The statistical methods are appropriate for the study design, which includes repeated measures and time-to-event data.	
Oberfeld, Helwe et al 2023	Y - he methodology clearly describes the study design as a retrospective study. The study involves the analysis of data collected from patient records at a tertiary care centre. The study does not describe a registry or explicitly state that it uses real-world data, although the data comes from real-world clinical practice.	PY - The dataset consists of surgical records from Massachusetts Eye and Ear. owever, there is no explicit statement on who designed the dataset, although it is implied that it was collected as part of routine clinical care, and the study does not explicitly state whether the dataset was designed for research or collected for other purposes.	Y - Datafields included: age, gender, preferred language, race/ethnicity, glaucoma type, best corrected visual acuity, medication burden, IOP, glaucoma stage pre-operatively, prior history of ocular surgery/laser procedure, postoperative complictions; further glaucoma surgery or laser capsultomy within 12 months.	Y - Included if postoperative report confirmed Phace/Hydrus or Phaco/KDB. Excluded if <6 weeks follow up, additional procedure at time of surgery, less than 18 years at time of surgery	Y - The study compares two interventions: Phaco/KDBand Phaco/Hydrus. The study also describes the surgical technique	Y - Primary outcomes - pattern of change in IOP and in number of glaucoma medications >12 months; secondary outcomes - pattern of change in the number of glucoma eyedrops and in the logarithm of the minimum angle of resolution (logMAR) as well as the procedure length and incidence of postoperative complications.	Y - The study uses several statistical methods: Descriptive Statistics, comparative Statistics: Kruskal-Wallis rank sum tests and Pearson's Chi-squared tests to assess differences in baseline characteristics between groups, generalized Estimating Equations (GEE): To assess changes in IOP, medication burden, number of eyedrops, and logMAR over time, accounting for repeated measures.<br>Kaplan-Meier (KM) survival estimates: To assess survival without additional intervention or medication. Log-rank tests were used to evaluate the statistical difference in survival. Methods to minimize confounding: The study adjusted for significant baseline differences (like IOP) in the GEE models. Subgroup analysis: The study conducted univariate KM analyses to assess for differences in survival based on glaucoma stage, non-White race/ethnicity, and sex	N - The study does not explicitly explain how missing data was addressed. It does mention attrition in both cohorts throughout postoperative timepoints. The study does not state whether missing data was imputed or if a complete case analysis was performed
Reitsamer et al 2022	Y - The study design is described as a multicentre, non-interventional, observational, retrospective chart review of medical records. The study uses real-world data collected	PY - The study mentions that the dataset comes from medical records of patients who underwent gel stent implantation at the participating centres. The dataset was not purposely designed for this research, but was	PY - The study does not provide a detailed description of structured data fields or define each item. However, it does mention the types of data collected, such as demographics, medical history, surgical details, IOP measurements, medication use, and complications.	Y - Inclusion: Patients with open-angle glaucoma (OAG) who underwent gel stent implantation alone or combined with cataract surgery between January 1, 2014, and October 1, 2015, with a minimum dataset including age, gender, IOP measurements, and IOP-lowering medications at baseline and during the study, and recorded dates	Y - The intervention is the XEN gel stent. The study describes the device as a 6-mm-long gelatin tube with a 45-μm lumen that connects the anterior chamber to the subconjunctival space. It is implanted ab interno. There isn't a direct comparator in	PY - Primary outcome measures: Mean changes in IOP and the number of topical IOP-lowering medications from medicated baseline, 3 years post-implantation. Secondary outcome measures: Mean changes	Y - The study used SAS software version 9.4 for statistical analyses. Continuous variables were reported using mean, standard deviation (SD), median, minimum, and maximum values. Categorical variables were reported using numbers and	N - The study explicitly states that only observed data were reported, without imputation of missing data. This means that if data for a particular time point or patient was not available, it was not included in the analysis for that specific measure.

	from nine European centres	collected as part of routine patient care. The study report was developed by ICON plc, Dublin Ireland. The study also notes that writing and editorial assistance were provided by Evidence Scientific Solutions and funded by Allergan (an AbbVie company).		of any secondary surgical interventions. Exclusion: Patients not meeting the inclusion criteria, and those who underwent gel stent implantation in combination with another procedure	this study, as it primarily evaluates the outcomes of gel stent implantation alone or combined with phacoemulsification.	in IOP and medication count from baseline at 1, 2, and 4 years post-implantation, if data were available. The study also assessed the proportions of eyes achieving specific IOP reductions (≥20%, ≥30%, ≥40%) and target IOP levels (≤18, ≤16, ≤14 mmHg). Complete and qualified success rates were also evaluated.Although visual field assessments were performed, the study notes that the schedules were not standardised, and the visual field mean deviation was assessed per standard local practice and as available.	percentages.Paired t-tests were used to evaluate differences in IOP or medication use from baseline to postoperative visits. The study performed pre-specified subgroup analyses based on the type of glaucoma (i.e., POAG) and treatment received (implant alone vs. phaco + implant).Sensitivity analyses were conducted including primary and secondary eyes. The study also used Kaplan-Meier curves to estimate the probability of maintaining overall success. The study did not explicitly discuss methods to minimise confounding.	
Salimi et al 2021	Y - the methodology clearly describes the study design.It is a single-surgeon, longitudinal, consecutive case series. The study assessed 36 months' worth of outcomes following cataract extraction with concomitant implantation of iStent inject trabecular micro-bypass stents. The study also acknowledges that it is a real-world study	PY - The study does not explicitly state who designed or funded the dataset. However, it does state that the data was collected at a single ophthalmology centre in Montreal, Canada. The data was extracted from patients' medical records. This implies that the dataset was not primarily designed for research but was collected as part of routine clinical practice and then used for research	Y - The study describes the data fields used. The study includes descriptions of the efficacy measures which consist of changes in mean IOP, medication use, the proportion of eyes with IOPs at different thresholds, eyes on 0 or ≥ 2 medications, eyes with reductions of medications, and the success rate (absence of secondary glaucoma interventions). The safety measures are described including intraoperative and postoperative adverse events, changes in best-corrected visual acuity (BCVA), cup-to-disc ratio (CDR), visual field mean deviation (VF MD) and secondary glaucoma interventions or surgeries. *	Y - Inclusion criteria include: Age above 18 years; Glaucomatous damage evidenced on RNFL imaging, ganglion cell analysis, or VF; Presence of cataracts; Need for reduction of IOP or glaucoma medications; Availability of 36-month follow-up data; Exclusion criteria include; Presence of nasal peripheral anterior synechiae or inability to visualise the nasal angle; Elevated episcleral venous pressure; Acute or chronic intraocular inflammation; The study notes that off-label uses of iStent inject (which at the time included PACG or severe glaucoma cases) were not excluded, due to the real-world nature of the study.	Y - The study focuses on the iStent inject, which consists of two second-generation trabecular micro-bypass stents. The procedure for implanting the iStent inject is described in detail, including the technique for identifying the areas for stent placement	Y - Yes, the study clearly describes pre-specified outcome measures and how they were assessed.Primary outcome measures included changes in mean IOP and medication usage over the 3-year follow-up.Secondary outcome measures included: Proportional analyses of eyes with IOP ≤ 18, ≤ 15, and ≤ 12mmHg; Eyes on 0 or ≥ 2 medications; Eyes with reductions of ≥ 0, ≥ 1, or ≥ 2 medication(s); The rate of success (absence of secondary glaucoma interventions); Safety measures included any intraoperative and postoperative adverse events, changes in best-corrected visual acuity (BCVA), cup-to-disc ratio (CDR), and visual field mean deviation (VF MD); IOP measurements were performed by the same surgeon using a calibrated	Y - The 3-year efficacy and safety outcomes were evaluated using generalized linear models, with Bonferroni adjustment for multiple comparisons. Kaplan-Meier analysis assessed the cumulative 3-year survival. Baseline predictors of outcomes were assessed using univariate and multivariate regression models. The study performed a pre-specified subgroup analysis for eyes with mild-to-moderate POAG. Supplementary analyses were performed on a subpopulation of the cohort including only 1 eye of each patient, selected at random to limit potential selection bias. The multivariate regression models helped to control for potential confounding factors when analysing baseline predictors of outcomes.	N - Postoperative efficacy data of eyes that underwent additional glaucoma interventions were censored in all analyses at subsequent follow-up time points. This means that data from these patients was not included after they had the additional intervention. However, other missing data is not addressed.

						Goldmann applanation tonometer		
Samuelson et al 2019	Y - The methodology section clearly describes the study design as a prospective, randomized, single-masked, controlled, multicenter clinical trial. It was designed to compare the iStent inject combined with cataract surgery with cataract surgery alone.	Y - The study was purposely designed for research to assess the safety and effectiveness of the iStent inject. The study indicates that Glaukos Corporation participated in and provided funding for the design and conduct of the study; the collection, management, and analysis of data; and the preparation of the manuscript.	Y - The study does define the parameters collected, including demographic, preoperative, operative, and postoperative data. Key measurements such as IOP, visual acuity, medications, and adverse events were all part of the structured data collection. Definitions for the various acronyms used in the study are included. However, these are not presented as an appendix or supplement.	Y - Inclusion criteria included: diagnosis of mild to moderate POAG; age-related cataract with BSCVA 20/40 or worse; screening IOP ≤24 mmHg on 1 to 3 medications; baseline unmedicated DIOP ≥21 mmHg and ≤36 mmHg, and at least 3 mmHg higher than medicated screening IOP; screening cup-to-disc (C:D) ratio of 0.8 or less; normal open-angle anatomy; and ability to provide an adequate, interpretable visual field. Exclusion criteria included: traumatic, uveitic, neovascular, or angle-closure glaucoma; history of incisional glaucoma surgery or certain laser treatments; visual field MD worse than -12 dB; ocular disease affecting safety or eligibility for washout; any condition that would preclude safe surgery or follow-up; fellow eye BSCVA worse than 20/80; functionally significant visual field loss; and visual field status at risk by the washout period.	Y - The study provides a clear description of the intervention device, the iStent inject Trabecular Micro-Bypass System (Model G2-M-IS). The device is described as a two-stent system that creates two bypasses through the trabecular meshwork.The comparator in this study is cataract surgery alone. The study compares the outcomes of cataract surgery with the iStent inject to those of cataract surgery without the device.	Y - The study clearly describes the pre-specified primary and secondary outcome measures. The primary effectiveness end point was a ≥20% reduction from baseline in diurnal IOP (DIOP) without ocular hypotensive medication at month 24. The secondary effectiveness end point was the 24-month DIOP reduction from baseline without medication.Safety measures included best spectacle-corrected visual acuity (BSCVA), slit-lamp and fundus examinations, gonioscopy, pachymetry, specular microscopy, visual field testing, adverse events (AEs), and complications	Y - The Fisher exact test was used to compare the two groups for the primary effectiveness endpoint.<br>The two-sample t-test was used to compare the two groups for the secondary effectiveness endpoint and the reduction in ocular hypotensive medications.<br>The study also analysed outcomes in an intent-to-treat cohort, which included all randomised subjects.<br>The study used a randomised design to minimise confounding.There was no pre-specified subgroup analysis described in the study methodology.	N - The study does not explicitly describe how missing data was addressed. However, it does note that some subjects did not undergo medication washout at month 24. The study reports that outcomes were analysed for both an effectiveness cohort and an intent-to-treat cohort. The intent-to-treat cohort included all randomized subjects even if they did not receive the intervention or complete follow up visits.
Samuelson et al 2021	Y - The study design is described as a "randomized controlled trial". It was a post-hoc analysis of data from the pivotal trial of the iStent inject device. The trial design involved randomizing patients in a 3:1 ratio to either the iStent inject group or a control group after uncomplicated cataract surgery. The study utilized data from the safety population, which included all randomised subjects analysed according to the treatment they actually received	PY - The dataset comes from the pivotal trial of cataract surgery plus iStent inject®. The study was sponsored by Glaukos Corp. Glaukos also had employees involved in the study and analysis. The dataset was purposely designed to assess the safety and effectiveness of the iStent inject® device, including the collection of patient-reported outcome (PRO) measures, but the PRO data was for "exploratory analyses" and not a primary endpoint. We could not find who manages and curated it.	Y - The study describes the structured data fields used for the two PRO measures: VFQ-25: Consists of 25 questions, with responses converted to a 0-to-100 score and averaged to create 12 subscale scores and an overall composite score. OSDI: Consists of 12 questions, with responses rated on a scale of 0 to 4 and used to compute a composite score and three subscale scores ranging from 0 to 100. The study defines PRO responders based on a "minimally important difference" (MID), calculated as half the standard deviation (SD) of baseline scores	PY - The study mentions that patients with mild-to-moderate POAG and cataract were included in the study. It notes that a total of 505 eyes were randomized. However, the specific inclusion and exclusion criteria for the pivotal trial are not detailed within this study. They are described as being available elsewhere.	PY- The intervention is the iStent inject®, a "second-generation trabecular micro-bypass stent system". It is described as a "micro-scale" device. The comparator is cataract surgery alone. The study is comparing the outcomes of cataract surgery with iStent inject implantation to cataract surgery alone. The surgical methodology of these has not been described.	PY - The study mentions that the primary effectiveness endpoint of the pivotal trial was a "20% reduction from baseline in DIOP without ocular hypotensive medication at month 24". The study's focus, however, is on secondary outcome measures, specifically patient-reported outcomes (PROs) using the VFQ-25 and OSDI questionnaires. These are "widely-used" and "validated" instruments. The study defines PRO responders based on a "minimally important difference" (MID), calculated as half the standard deviation (SD) of baseline scores. The PRO measures were administered at baseline	Y - For baseline demographic and clinical characteristics, comparisons were made using t-tests for mean age, and χ^2 tests were used for age category, sex, race, number of hypotensive medications, and prostaglandin use. The percentages of VFQ-25 and OSDI PRO responders at various follow-up visits were compared between treatment groups using a logistic generalised linear mixed model for repeated measures. A χ^2 test was used to compare the percentage of patients free of medication at month 24 by PRO response. To identify factors associated with the likelihood of being a composite score responder for either the VFQ-25 or the OSDI, two mixed effects logistic models were explored. The study also performed some subgroup analyses, though they	Y - The majority of patients completed the questionnaires consistently at each time interval, with completion rates ranging from 91.6% to 99.2% across both instruments and all time points. The study states that there was no need to further investigate the impact of missing data, as they were accounted for in the analyses. The mixed models for repeated measures used in the analysis accounted for missing data by using the correlation among within-patient repeated measures.

						and at months 1, 6, 12, and 24. The results of the questionnaires were used to calculate composite and subscale scores, and to define PRO responders	were not pre-specified, according to the authors. The study used randomization to minimise confounding.	
Schlenker et al 2017	Y - The methodology clearly describes the study design. It is described as an "investigator-initiated, international, multicenter, retrospective cohort study". The study analyses real-world data from consecutive patients who underwent either microstent insertion or trabeculectomy at four different academic centres.	Y - The study describes the dataset as being derived from consecutive patients who underwent either microstent or trabeculectomy surgery between January 1, 2011, and July 31, 2015. The data was collected at four academic ophthalmology centres. The study is described as "investigator-initiated" and designed for research.	Y - The study describes the data collected through chart reviews, which include: Demographics (age, eye, gender, ethnicity, site, diabetes status). Ocular characteristics (best-corrected visual acuity [BCVA]; IOP at which the decision for surgery was made; glaucoma medications; glaucoma diagnosis; cup-to-disc ratio; mean deviation; and history of previous cataract surgery, laser peripheral iridotomy, or laser trabeculoplasty)	Y - Inclusion criteria: Patients between 30 and 90 years of age with various types of glaucoma (primary open-angle, pseudoexfoliation, pigment dispersion, normal-tension, angle-recession, combined mechanism, history of angle-closure, or juvenile glaucoma) with above-target IOP on maximum medical therapy were included. Exclusion criteria: Patients with prior incisional filtering glaucoma surgery, neovascular glaucoma, uveitic glaucoma, iridocorneal endothelial syndrome, Axenfeld-Rieger syndrome, fibrous or epithelial downgrowth, previous corneal graft surgery, previous retinal surgery, or less than 1 month of follow-up were excluded	Y - Yes, there is a clear description of both the intervention and the comparator. Intervention: The study monitors the Xen 45 microstent, a 6-mm gelatin microfistula with a 45-mm lumen, implanted ab interno with MMC. The procedure is detailed, including the use of MMC, incisions, and the method of placement. Comparator: The comparator is trabeculectomy with MMC, a standard surgical procedure for glaucoma. The procedure is also described in detail.	Y - Yes, the study describes the pre-specified primary and secondary outcome measures and how they were assessed. Primary outcome: The hazard ratio (HR) of failure, with failure defined as 2 consecutive IOP readings of less than 6 mmHg with vision loss, or more than 17 mmHg without any glaucoma medications, reoperation, or loss of light perception vision. Secondary outcomes: These included IOP thresholds (6-14 mmHg and 6-21 mmHg) with and without medications, median IOP, medication use, best-corrected visual acuity (BCVA) in logMAR units, postoperative interventions, complications, and reoperations	Y - Yes, the study describes the statistical methods used. Categorical characteristics were described using proportions and compared using Fisher exact tests. Continuous outcomes were described as means or medians and compared using t-tests or Wilcoxon tests depending on normality. Kaplan-Meier curves were used to display the association between the intervention and the survival outcomes. Cox proportional hazard models were used to assess the effect of variables on surgical failure. The study included subgroup analyses, a first-eye only analysis to address within-patient correlation, and sensitivity analyses to assess the robustness of the results. Multivariable Cox proportional hazard models were used to adjust for potential confounders. The study also stratified the models by surgical site. Effect modification was explored by adding interaction terms	PY - The study explains how missing data was handled for some analyses. The last observation carried forward (LOCF) method was used for eyes that underwent reoperation, when analysing medication use, IOP and BCVA. The LOCF method involves imputing missing data by using the last available observation. Additionally, the study censored for reoperations in an additional analysis. However, there is no specific discussion about how missing data was handled for other variables besides medication use, IOP and BCVA, and no specific mention about whether any other missing data were imputed.
Strzalkowska et al 2021	Y - The study is a retrospective analysis of patient data from the Trabectome Study Group database, and it employs an exact matching method to compare two surgical procedures. The use of a registry and analysis of real-world data are not explicitly stated in the methodology.	PY - The AIT dataset appears to have been collected as part of a post-market surveillance requirement by the FDA, implying that it was initially designed for regulatory purposes rather than specifically for research. The TRAB data was collected by a single surgeon (FG) at a single site. The study does not specify if this data was collected as part of a routine database or as a	PY - The study mentions the data collected, including medical history, best-corrected visual acuity (BCVA), intraocular pressure (IOP), topical glaucoma medications, events leading to failure, and serious vision-threatening complications. The study does not provide a specific data dictionary with a detailed explanation of each field, its format, or possible values.There is no appendix or supplementary material that defines the data fields.	PY - There is a detailed account of the inclusion and exclusion criteria for subjects in the study.	Y - There is a description of AIT, and a comparator (trabeculectomy) is applicable in this study	Y - The primary outcome measure was success, defined by a composite of three criteria. Assessment methods for all variables are detailed (eg. Goldmann tonometry for IOP)	Y - Uses descriptive statistics, inferential statistics, Kaplan Meier plots. Exact-matching is used to minimise confounding. No subgroup analysis performed.	N - While the study details the statistical methods used, such as the Mann-Whitney U test, chi-squared test, and Welch two-sample t-tests, as well as the Kaplan-Meier plot, there is no specific discussion of how missing data was handled.

		research-oriented dataset. It does not provide complete details about who designed, funds, curates, and manages the databases						
Tojo et al 2020	Y - The study is a single-facility, non-randomized, observational retrospective study. Researchers looked back at data from patients who had already undergone trabectome surgery at one hospital. It is not a registry, nor is it explicitly described as an analysis of real-world data, though the data is from a real-world clinical setting	Y - The dataset was designed for research purposes, specifically to evaluate the outcomes of trabectome surgery in relation to preoperative IOP. The study was conducted at the University of Toyama, and no funding was received for this research.<br>The dataset was curated and managed by the researchers at the University of Toyama. One glaucoma specialist (N.T.) diagnosed all cases of glaucoma and decided whether trabectome surgery should be performed	Y - The study does not include a specific appendix or supplement describing structured data fields, but it does define and present data for the following: Patient demographics (age, gender), Follow-up period, Preoperative and postoperative intraocular pressure (IOP), Mean deviation (MD) in visual field tests, Types of glaucoma (POAG, PACG, PEXG, SG), History of selective laser trabeculoplasty (SLT), Type of surgery (trabectome alone or combined with cataract surgery), Central corneal thickness (CCT), Number of glaucoma medications used, IOP reduction rates	PY - Inclusion: The study analyzed the cases of 204 consecutive patients (246 eyes) who underwent trabectome surgery and were followed for more than 2 years. However, no description regarding glaucoma type or any other confounding factors was given. Exclusion: The study did not include data for patients who underwent additional glaucoma surgery. However, it is noted that the study included patients who underwent additional glaucoma surgery after the study period ended	Y - The study focuses on trabectome surgery using the trabectome device (Neomedix, Tustin, CA, US). The procedure involves a 1.7-mm corneal incision to ablate the trabecular meshwork and inner wall of Schlemm's canal. The study does not have a direct comparator group, but it compares surgical outcomes across three groups of patients with different preoperative IOP values: low (<18 mmHg), mid (18–26 mmHg), and high (>26 mmHg)	Y - The primary outcome was the success rate of trabectome surgery in each of the three preoperative IOP groups, assessed using Kaplan-Meier analysis. The study also investigated factors that can affect surgical outcomes such as age, CCT, mean deviation (MD), history of SLT, and simultaneous cataract surgery. The study used Goldmann applanation tonometry (GAT) for IOP measurements. The study uses the Humphrey Field Analyzer for automated perimetry. These are both standard and validated methods.	Y - Paired t-tests and Fisher's exact tests were used to compare the three patient groups. Wilcoxon signed-rank test was used to compare the same patients' data. Kaplan–Meier method was used to compare group success rates along with the log-rank test. Multivariate analysis was used to examine factors that may affect surgical outcomes. The study used pre-specified subgroup analysis, dividing patients into three groups based on their preoperative IOP values and comparing the surgical outcomes of each group using Kaplan-Meier analysis and various cut-off points for success. The study also used multivariate analysis to adjust for confounders	N - The study does not provide a detailed explanation of how missing data was addressed. However, it is noted that some patients did not have follow-up data at all time points, and the sample size for analysis varied accordingly.
Vukmirovic et al 2023	Y - The methodology describes the study design as a retrospective, non-randomised clinical study conducted at a single centre. The study analysed data collected from patients who underwent gelatin stent surgery over a 24-month period. This indicates an analysis of real-world data.	Y - The data was gathered from a specialised database created using Microsoft Access, used to record clinical information. * The study was conducted at the Lions Eye Institute in Perth, Western Australia, and all surgeries were performed by a single surgeon. * The study was funded by the National Health and Medical Research Council.	Y - Structured data fields are described. These include: Intraocular pressure (IOP) at each visit, Ethnicity, Type of glaucoma, Site of entry of gelatin stent in the iridocorneal angle, 5-FU needling, Complications, Preoperative IOP (defined as the IOP recorded within 40 days prior to surgery) The source does not include an appendix or supplement to define these data fields further.	Y - Inclusion criteria included patients over 18 years with primary open angle or angle closure glaucoma, primary congenital glaucoma, or secondary glaucoma who had gelatin stent surgery. Angle closure glaucoma (ACG) was defined as eyes that had primary angle closure with iris to trabecular meshwork contact and had laser iridotomy or cataract extraction with resultant significant angle opening. Exclusion criteria included eyes that had not undergone gelatin stent surgery, and eyes with totally occluded angles and neovascular glaucoma	Y - The surgical technique is described including the use of a Yu-Morgan speculum, clear corneal incision, the location of stent placement, use of sodium hyaluronate, and the delivery of the stent	Y - Included IOP at each visit, ethnicity, type of glaucoma, site of entry of gelatin stent in the irodocorneal angle (based on gonioscopic examination), 5-FU needling and complications. Preoperative IOP was defined as the IOP recorded within 40 days prior to surgery. If there were two or more visits in this period, an average of the IOP measurements was calculated and taken to be the preoperative IOP.	Y, IOP trends analysed by calculating mean, standard deviation, median and range values. Results were described as means and standard deviations as well as counts and percentages. ANOVA multivariate analysis was used to compare between subgroups.	N - There is no explicit explanation for how missing data was addressed. However, the study mentions that some data was omitted from analysis because it was not recorded.
Yang et al 2023	Y - The study is a multicentre, retrospective cohort study. Each cohort was defined as type of MIGs	Y - American Academy of Ophthalmology's IRIS registry. Dataset is HIPAA compliant. No mention of other funding sources.	Y - Key structured data fields included demographics, glaucoma diagnosis, severity, IOP, VA, MIGS procedures, reoperation and type of procedure, postoperative	PY - Study initially includes iStent and Hydrus within inclusion criteria but then removes them due to difficulty in CPT codes within exclusion criteria.	PY - The study provides a clear description of the specific MIGS procedures being monitored. However,	Y - The primary outcome was the time to reoperation, while secondary outcomes included changes in IOP	Y - Descriptive statistics, Analysis of variance (ANOVA) with Bonferroni correction, and the chi-square test and Fisher exact test were used to compare	Y - The study used multiple imputation to address missing data in the multivariable analysis. The study used linear mixed-effects models to analyze changes in mean

	+/- phacoemulsifcation. It uses data from the American Academy of Ophthalmology's Intelligent Research in Sight (IRIS) Registry.	Collected as a routine database.	complications. However, this information is not provided in a separate appendix or supplement.		each MIGs procedure is not explicitly described.	and VA, and the occurrence of postoperative complications.	groups. Linear mixed-effects models were used to analyse changes in mean intraocular pressure (IOP) and visual acuity (VA) over time, accounting for missing data. Student's t-tests were used to compare percentage change from baseline at 1 year. Kaplan-Meier survival analysis was used to assess reoperation rates, and log-rank tests were used to compare survival curves. Multivariable Cox proportional hazard models were used to identify factors predictive of reoperation, using multiple imputation to manage missing data, and adjusting for confounding variables such as age, sex, race, ethnicity, glaucoma diagnosis, glaucoma severity, and baseline IOP. Subgroup analyses was performed by stratifying data based on whether or not the MIGS procedure was performed concurrently with phacoemulsification,	intraocular pressure (IOP) and mean visual acuity (VA) over time. Patients with poor data quality, top 0.1% of IOP outliers, duplicate IOP measurements and VA measurements in Jaegar notion was excluded.
