# Supplementary material for: Evaluating Long-Term Effectiveness and Safety of Glaucoma Surgical Innovations: A Systematic Review
Source: J Glaucoma. 2026 Oct 16;35(11):838–45. doi: 10.1097/IJG.0000000000002770 (PMC13722426; doi:10.1097/IJG.0000000000002770)
Supplement: Supplementary file 5 [file ijg-35-838-s005.pdf]

Supplemental Table 5. Data extraction as per IDEAL Stage 4 Framework for Results (rated Yes, Partially Yes or No)

	Results		
**Authors + Year**	**9a. Is there a description of patient demographic and clinical characteristics, and hospital/centre/operator characteristics? (Y, PY, N)**	**9b. Do results Indicate extent of missing data for each variable of interest, if appropriate? (Y, PY, N)**	**10. Do results report main pre-specified outcome measures, including outcome variations among pre-specified subgroups and adjustments for confounders, when applicable? (Y, PY, N)**
Ahmed et al 2022	PY - Yes, there is a description of patient demographic and clinical characteristics. The study notes that baseline participant demographic and preoperative characteristics were described previously and are provided in a supplementary table. The study mentions that it was conducted at 38 investigational centers worldwide (26 sites in the United States and 12 international sites). However, there is no further description of the characteristics of these hospitals or centers. The study notes that most surgeons had limited prior experience with the HMS implantation technique. However, there is no further description of the individual operators.	N - The study reports that five-year follow-up was completed for 308 of 369 participants (83.4%) in the HMS group and 134 of 187 participants (71.7%) in the CS group. * The study mentions that all patients who completed the 5-year postoperative examination or who demonstrated secondary glaucoma surgery within the follow-up period were included in the analysis. * The study does not explicitly state the extent of missing data for each specific variable of interest	Y - Yes, the results report the main pre-specified outcome measures. The study reports on IOP, medication use, and the need for repeat glaucoma surgery. It presents data on the proportion of eyes with an IOP of 18 mmHg or less without medications, the likelihood of IOP reduction of 20% or more without medications, the number of glaucoma medications, and the cumulative risk of incisional glaucoma surgery. The study also reports on the rate of endothelial cell loss and other safety outcomes
Ahmed et al 2023	PY - There is no detailed information about the participating hospitals or centres, such as the type of facility, location, or size. The study does mention that it was a multicenter trial, but does not give details of these centres. There is no information about the surgeons/operators involved in the study, such as their experience level or training.	N - The study describes how the study population changed over time and looks at possible selection bias. It does not provide details on the extent of missing data for each specific variable of interest. It does not specify how many patients did not have data for any variable at each time point, or what statistical methods were used to account for any missing data points within the longitudinal follow-up.	PY - the results do report the main pre-specified outcome measures (incidence of clinically relevant complications, mean change of ECD, proportion of patients with >30% endothelial cell loss, etc), including some outcome variations among pre-specified subgroups [PY]. However, the study does not explicitly describe adjustments for confounders
Arnould et al 2024	PY - There is a description of patient demographic and clinical characteristics. There were 12 surgeons contributing to the dataset, with a mean number of surgery cases of 24. The study is a multicentre study, using data from an international registry. Further information is not provided re: training level of surgeons.	PY - The results do not explicitly indicate the extent of missing data for each variable, but the study does use a "last observation carried forward" (LOCF) analysis to address missing data due to secondary procedures. The number of procedures eligible for analysis at each follow up time point is stated. The number of eyes and patients included decreases at each time point, which is explained by the fact that as a real world study surgeons enter consecutive patients into the registry and so there will be a smaller number of patients at each follow-up window as they reach new time points of follow up.	Y - The study reports both complete success (CS) and qualified success (QS) rates at 24 months for the primary outcome of IOP ≤18 mm Hg, ≥6 mm Hg, and ≥20% reduction from baseline. The CS rate was 26% and the QS rate was 48%. The study also reports CS and QS rates at different IOP thresholds (12, 15 and 21 mm Hg). It also reports on IOP changes, medication use, adverse events, and secondary glaucoma procedures. The study includes an analysis of outcomes stratified by Xen stand-alone and Xen+cataract groups. The study used modified linear models and Cox proportional hazard models to adjust for confounders such as gender, age, preoperative IOP, and preoperative number of medications.
Bambo et al, 2024	PY - the study provides comprehensive data on the demographic and clinical characteristics of the patients, including their age, sex, race, glaucoma type, previous surgical history, lens status, preoperative IOP, medication use, and other relevant clinical measures. The study also provides some information about the study setting as being a single centre, and that all surgeries were done by the same surgeons (but did not provide level of surgeons)	N - The study does not specify the exact extent of missing data for each variable, it does note the availability of data for some variables, and it explicitly states that the LOCF method was used to address missing data	Y - the study provides a detailed analysis of the pre-specified outcome measures, compares outcomes between the XEN-alone and XEN+Phaco subgroups, and uses appropriate statistical methods to adjust for confounders, which strengthens the findings and conclusions
Clement et al 2020	Y - The study provides a comprehensive description of patient demographic and clinical characteristics, as well as key information about the multi-centre, multi-surgeon setting in which it was conducted	N - While the study provides the sample size for each variable, which allows for an understanding of how much data was available for analysis, it does not offer an explanation of the reasons for missing data and does not outline any methods for statistically handling the missing data	Y - The study reports the main pre-specified outcomes and provides subgroup analysis across glaucoma types, but does not describe statistical adjustments for confounders
Dang et al 2018	PY - there is a description of patient demographic and clinical characteristics. The study does not explicitly describe the operator characteristics.	PY - The results indicate the extent of missing data for some variables. The study uses 'NR' (Not Recorded) to indicate missing data for visual acuity (30%), visual field (46.7%), disc cup/disc ratio (23.3%), and lens status (17.5%). The impact of this missing data on the results is not explicitly discussed in the study.	Y - The primary outcome, reduction in IOP, is reported at various time points during the 2-year follow-up. The secondary outcome, reduction in glaucoma medications, is also reported. The number of glaucoma medications reduced significantly from 2.20 ± 0.17 at baseline to a range of 1.20-1.40 during the 2-year follow-up. The overall success rate was 80% at 2 years. Patients who received combined surgery (Trabectome with phacoemulsification and intraocular lens implantation) had a 100% success rate, compared to 77% for Trabectome alone. Patients with a history of prior trabeculectomy or tube shunt implantation had a similar success rate compared to those without such a history (79% vs. 82%). The study attempted to adjust for confounders but did not identify any risk factors for failure using univariate logistic regression. Multivariate logistic regression was not performed due to the small sample size.
Deitz et al 2023	Y - The study included 394 eyes of 262 patients. The study reports the age and gender of patients, noting no significant difference between the	N - The study does not explicitly quantify the amount of missing data for each variable. However, it does state that patients with missing pre-	Y - The study reports the mean IOP at baseline and at each follow-up visit for all three groups, with statistically significant reductions compared to pre-

	surgical groups. The mean age was approximately 72-73 years across the groups. The study reports the distribution of glaucoma types, with the majority of patients having primary open-angle glaucoma (POAG), followed by normal tension glaucoma (NTG), and pseudoexfoliation glaucoma (PXG). The distribution of glaucoma type was not significantly different between the groups.The study reports the severity of glaucoma, noting that the proportion of patients with mild, moderate, and severe glaucoma varied significantly between surgical groups. There was a higher proportion of severe glaucoma cases in the phaco/ECP group compared to the phaco/iStent group.The study also notes the mean pre-operative intraocular pressure (IOP) and number of glaucoma medications used pre-operatively	operative IOP data or without at least 6 months of follow-up were excluded from the analysis	operative IOP at most time points. The study also reports the mean number of glaucoma medications used at baseline and at each follow-up visit, with statistically significant reductions in the number of medications used postoperatively in each group. The study also reports the success rate at each time point, defined as IOP reduction of at least 20% and/or reduction in at least one glaucoma medication. The study includes a pre-specified subgroup analysis excluding severe glaucoma cases and reports outcomes in this subgroup.
Ferguson et al 2020	PY - describes demographic characteristics (age, gender) and other clinical characteristics. Describes site as a private practice in South Dakota. Does not provide specific details about surgeon's level of experience or specific training.	N - The study does not provide an explicit breakdown of missing data for each variable. However, the study does state that at 6 years postoperatively, data was available for 120 of the 411 eyes included at baseline. The study acknowledges the loss to follow-up as a limitation, and notes that it is possible some patients lost to follow-up may have undergone additional procedures elsewhere.	Y - The results report the main pre-specified outcome measures (mean IOP and number of glaucoma medications) at various time points.The results also include outcome variations among pre-specified subgroups. A subgroup of eyes with 6-year data available was used to directly compare 6-year outcomes with baseline data. Eyes were stratified by baseline IOP (e.g., ≥18 mmHg, 18-20 mmHg, ≥21 mmHg, and <18 mmHg) to assess the magnitude of IOP reduction. Outcomes were reported separately for eyes with mild, moderate, and severe glaucoma. Eyes were stratified by the number of medications being used at baseline, including those on 2 or more and 3 or more medications.The study does not explicitly mention adjustments for confounders.
Gabbay et al 2021	Y - There is a description of patient demographic and clinical characteristics. The study was conducted at a university-affiliated National Health Service (NHS) regional hospital in the UK. XEN45 surgeries were performed by four surgeons with expertise in glaucoma (two consultants and two fellows), who had all undergone full training.	PY - Study does show how many patients had data available for analysis at each time point, it does not quantify the extent of missing data for each variable of interest.	Y - Mean IOP and number of antihypertensive medications used are reported at each time point, along with statistical significance of changes from baseline. The proportion of patients using one or more antihypertensive medications at each timepoint is also reported.Outcome variations among pre-specified subgroups are reported:Success rates were stratified by target IOP (<21, <18, and <15 mmHg).Subgroup analysis by ethnicity and gender showed that non-Caucasians and females were more likely to fail.Adjustments for confounders are addressed through multivariable Cox regression analysis.
Hengerer et al 2022	Y - The study provides a detailed description of patient demographics and clinical characteristics	N - Nearly all eyes (121/125, or 97%) completed the 5-year follow-up. The study does not specify the small amount of missing data for individual variables, but the high completion rate suggests that the impact of any missing data on the study is likely to be minimal.	Y - The study notes that while cataract extraction can lower IOP, it has a temporary effect. The fact that the IOP reduction was consistent over 5 years in the combined and standalone groups suggests that the IOP reduction is a result of the iStent device
Holmes et al 2022	PY - The study provides detailed information on patient demographics and clinical characteristics, and includes some information on the hospital/centre/operator characteristics. The data was captured from multiple surgeons from various locations via a web based registry	N - The study does not explicitly detail the extent of missing data for each variable of interest, but it does address missing data in a specific context eg. exclusion criteria, last observation carried forward, etc	Y - The primary outcome was a comparison of mean intraocular pressure (IOP) at 24 months between the two groups (Hydrus Microstent and iStent inject).<br>Secondary outcomes included: Differences in medication use, difference in percentage IOP reduction, adverse event rates and the proportion of patients achieving complete success (CS) or qualified success (QS) at different IOP levels (15, 18, and 21 mmHg). Adjustments for confounders done through statistical models (eg. linear mixed models.
Inatani et al 2022	PY - There is a description of patient demographics and clinical characteristics. The study does not provide detailed information on hospital/centre/operator characteristics. While the number of sites is mentioned, details like the type of hospitals or the surgeons' experience levels are not included.	PY - The study notes that 211 eyes had data at POM6, 202 at POM12, and 190 at POM24. The study explains that the majority of the 42 eyes that left the study by POM24 were due to non-clinical factors such as not being present at the hospital or change of address. The results also mention the number of eyes that had both pre-operative and POM24 data for IOP and medications and use this data for analysis. The study also indicates the number of eyes included in analysis of central corneal ECD	Y - The results report the main pre-specified outcome measures. The study reports the reduction in mean IOP from 17.6 mmHg preoperatively to 14.3 mmHg at POM24, along with statistical significance. It reports a reduction in the mean number of medications from 2.2 preoperatively to 0.7 at POM24, with statistical significance. Multilevel mixed models were used to adjust for the confounding effects of using the same or different tonometers for IOP measurements.
Katz et al 2018	PY - A prior publication from this study provided the complete preoperative demographics and ocular parameters of subjects in this study.	N - The study does not provide a detailed breakdown of the number of missing data points for each variable at each time point.	PY - Does report the main pre-specified outcome measures (IOP, medication use, safety parameters), including some outcome variations among pre-specified subgroups, but does not explicitly detail adjustments for confounders
Lee et al 2020	Y - The study includes detailed descriptions of patient demographics such as age, gender, and presence and type of open-angle glaucoma (OAG). It also includes relevant clinical characteristics like preoperative intraocular pressure (IOP) and number of medications used. The study also clearly describes that it was a single-surgeon case series in a subspecialty private practice in Brisbane, Australia	N - The study states the overall percentage of missing data across follow-up visits which ranged from 6-15% for the OAG patients. The study also notes that "For some patients, the 6 months data was not available, but were reviewed later at 12 and/or 24 months". However, the specific amount of missing data for each variable (e.g. IOP, medications) at each time point is not detailed	Y - The study reports the main pre-specified outcome measures which were intraocular pressure (IOP) and the number of glaucoma medications. The results are reported for each of the three study groups (phacoemulsification alone, combined iStent, combined Hydrus) at various time points (preoperative, 1 day, 1 month, 6 months, 12 months, and 24 months). The study also includes analysis that compares the changes in IOP and

			medications between the three groups and at different time points, thereby reporting outcome variations among these pre-specified subgroups
Mitchell et al 2024	PY - IRIS registry is a national database so no need for description of hospital/centre/operator characteristics	PY - Because the IRIS Registry is retrospective, the size of the cohorts was limited to those with nonmissing outcome data.	Y - the study clearly reports the main pre-specified outcome measure, which is reoperation rate, following angle-based minimally invasive glaucoma surgery (ab-MIGS).
Montesano et al 2023	PY - The study describes the following patient demographic eg. age, sex, and race and clinical characteristics. The study was conducted across 38 centers but did not describe specifics of this or the individual operators.	Y - The study reports the extent of missing data. A total of 3701 VFs from 554 patients (99.6%) were available for analysis. Of these, 121 VFs were excluded because of poor reliability. For 5 subjects, no reliable VFs were available post-surgery. The study reports that the mean number of VFs in the follow-up was 5.7 (SD 1.3) with a median of 6 tests. The study reports that the attrition rate was < 10%, with a higher proportion of patients lost in the CS arm (12%) compared to the CS-HMS arm (4%). A sensitivity analysis reduced attrition rates	Y - The study reports the main pre-specified outcome measures. Primary outcome: The rate of progression (RoP) of VF damage was significantly faster in the CS group compared to the CS-HMS group/
Oberfeld et al 2023	Y - The study provides a detailed description of patient demographic and clinical characteristics, as well as hospital/centre and operator characteristics	N - The study does not explicitly state the amount of missing data, but it does present the number of patients at each timepoint in Tables 2 and 3 which demonstrates that missing data increases with time. The handling of missing data was mentioned, but the extent of missing data for each variable was not given.	Y - The study reports its main pre-specified outcome measures, compares the outcomes between the three surgical groups (which can be seen as a type of subgroup analysis) and makes adjustments for potential confounding factors through statistical modelling.
Oberfeld, Helwe et al 2023	PY - Baseline demographics and clinical characteristics detailed. However, no description of hospital/centre/operator characteristics	N - he study does not explicitly indicate the extent of missing data for each variable of interest. The study does mention attrition in both cohorts throughout the postoperative timepoints, and that some patients were excluded due to less than 6 weeks follow up.	Y - The results report on the main pre-specified outcome measures. The study reports the mean IOP reduction and medication burden at various time points, using Generalized Estimating Equations (GEE) models. The results show significant reductions in IOP and medication burden in both groups.
Reitsamer et al 2022	PY - The study provides a description of patient demographic and clinical characteristics. It does not provide detailed information about the hospitals, centres or surgeons involved in the study	PY - The study does indicate the extent of missing data by reporting the number of eyes contributing data at each time point.The study also specifies that 163 eyes were included in the primary outcome measures analysis because they had both IOP and IOP-lowering medication data at baseline.The study states that 33 and 30 eyes were included in the 4 year safety and effectiveness analyses, respectively	Y - The study reports the main pre-specified outcome measures. Mean IOP decreased from 20.7 mmHg at baseline to 13.9 mmHg at 3 years, and mean medication use decreased from 2.5 to 1.1 at 3 years.Secondary outcomes: The study presents results for the proportions of eyes achieving specific IOP reductions and target IOP levels. Subgroup Analyses: Outcomes were also reported fort he POAG subgroup, the implant alone and phaco + implant subgroups. The study did not adjust for confounders, as is typical of observational studies that do not manipulate variables.
Salimi et al 2021	Y - There is a detailed description of patient demographics and clinical characteristics. It was a single-centre study conducted at an academic ophthalmology centre in Montreal, Canada. All procedures were performed by a single surgeon	PY - The study provides the sample size (N) for each variable at each time point (preoperative, 12 months, 24 months and 36 months), indicating how many data points were available for analysis. For example, while the study began with 124 eyes, by 36 months, some follow-up data was only available for 92 eyes when assessing IOP and medication use.	Y - The results report on pre-specified outcome measures and variations among subgroups.
Samuelson et al 2019	PY - Demographic characteristics included age, gender, and race/ethnicity. Preoperative ocular characteristics included the number of ocular hypotensive medications, medicated IOP at screening, unmedicated IOP at baseline, BSCVA, visual field MD, central corneal thickness, Shaffer angle grade, and vertical C:D ratio.The study was conducted at 41 sites. The study does not provide detailed information on hospital/centre/operator characteristics.	N - The study does not explicitly state the extent of missing data for each variable. The results do indicate that a small portion of the sample (12 in the treatment group and 6 in the control group) did not undergo medication washout at month 24. The study also reports outcomes for the 7 treatment eyes that were not implanted with 2 stents.	Y - The results section reports the main pre-specified outcome measures.The primary effectiveness endpoint was met, with 75.8% of the treatment group and 61.9% of the control group achieving a clinically significant (≥20%) reduction in medication-free DIOP from baseline at 24 months. The mean reduction in medication-free DIOP from baseline to 24 months was 7.0 mmHg in the treatment group and 5.4 mmHg in the control group. The study also reported that 63.2% of treatment eyes and 50.0% of control eyes had a month 24 medication-free DIOP of ≤18 mmHg. There were no pre-specified subgroup analyses, and no adjustments were made for confounders as the randomisation process was designed to minimise confounding.
Samuelson et al 2021	PY - The study provides a description of patient demographic and clinical characteristics. States conducted at 41 clinical sites but does not provide detailed information about hospital/centre/operator characteristics.	N - The results do not indicate the extent of missing data for each variable of interest individually. However, the study does report that the completion rates for the questionnaires were high, ranging from 91.6% to 99.2% across both instruments and all time points, and similar between groups.	PY - The study does report on the main outcome measures which were not pre-specified, as the PRO data were gathered for exploratory analyses and not intended for the trial endpoints. The main outcome measures were the Visual Function Questionnaire (VFQ-25) and the Ocular Surface Disease Index (OSDI) scores
Schlenker et al 2017	Y - The study provides a detailed description of patient demographic and clinical characteristics. The study was conducted at four academic ophthalmology centers located in Toronto, Canada; Frankfurt, Germany; Salzburg, Austria; and Leuven, Belgium. The study notes that almost half of the surgeries were performed in Canada. There were some differences in the surgical technique and post-operative management used at different sites. Performed by academis surgeons, all relatively new to procedure of microstenting.	Y - Yes, the study reports the main pre-specified outcome measures, including variations among subgroups and adjustments for confounders.	Y - The study reports the main pre-specified outcome measures, including variations among subgroups and adjustments for confounders.
Strzalkowska et al 2021	PY - Patient demographic information provided alongside diagnosis and prior procedure information. Hospital/Centre/Operator characteristics for	N - The sources do not explicitly indicate the extent of missing data for each variable of interest. Study design does not support this.	PY - Data fields for each timepoint not available in a table format

	both groups provided. AIT data for individual surgeons/centres not available.		
Tojo et al 2020	Y - Basic patient demographic and ophthalmic data was presented. The study was conducted at a single facility, the University of Toyama. All surgeries were performed by one surgeon (N.T.) using the same surgical protocol.	N - The study reported the number of patients included in the analysis at each time point. The results also present a summary of the number of patients at each time point for each IOP group. However, how this is addressed or the impact it has is not detailed.	Y - The study reports the main pre-specified outcome measures and outcome variations, including adjustments for confounders.
Vukmirovic et al 2023	Y - Baseline demographics and clinical characteristics detailed.The study was conducted at a single centre, the Lions Eye Institute in Perth, Western Australia.<br>All surgeries were performed by a single surgeon.	N - The results indicate where data is missing, but not for every variable. For example, the study states that:<br>"Eighty-two patients were omitted as they had no recorded pre medication usage data".<br>"Seventy-nine patients were omitted as they either had no recorded site of entry data or an unknown site of entry"	Y - The results report the main pre-specified outcome measures.<br>Adjustments for confounders were made through the use of:<br>multivariate mixed effects Cox proportional analysis and a mixed effects model was used to deal with possible patient, intereye and surgical dates correlating within a subject
Yang et al 2023	PY - IRIS registry is a national database so data was de-identified to be compliant with HIPAA. Thus, no information of hospital and opeator characteristics is provided.	Y - Note of missing data for various demographic and baseline ocular characteristics were made for: age (12.5%), gender (0.4%), race (15.0%), ethnicity (14.3%), diagnosis (54.7%), and severity (53.1%)	Y - Study reports results of primary outcome (time to reoperation) and secondary outcomes.
