# Supplementary material for: Evaluating Long-Term Effectiveness and Safety of Glaucoma Surgical Innovations: A Systematic Review
Source: J Glaucoma. 2026 Oct 16;35(11):838–45. doi: 10.1097/IJG.0000000000002770 (PMC13722426; doi:10.1097/IJG.0000000000002770)
Supplement: Supplementary file 6 [file ijg-35-838-s006.pdf]

Supplementary Table 6. Data extraction as per IDEAL Stage 4 Framework for Discussion (rated Yes, Partially Yes or No)

-	**Discussion**	
**Authors + Year**	**11. Is there discussion of limitations, addressing sources of potential bias, known confounders, missing data, and secular trends, including a discussion of the implications of using data that was not originally collected to answer the specific research question, where appropriate? (Y, PY, N)**	**12. Is there evidence of conclusions and relevance, including a discussion of the overall interpretation of results, the external validity, and any implications for policy and practice? (Y, PY, N)**
Ahmed et al 2022	PY - The study notes that it was not possible to mask the surgeon to the treatment group during postoperative examinations, which is a potential source of bias. The study acknowledges that most surgeons had limited prior experience with the HMS implantation technique. The study excluded patients with secondary open angle glaucomas, which limits the generalizability of the results. The inclusion criteria limited the study population to those with mild and moderate disease who could tolerate the medication washout. There is no mention of secular trends or a discussion of the implications of using data that was not originally collected to answer the specific research question.	Y - The study concludes that the addition of a Schlemm's canal microstent in conjunction with CS was safe, resulted in lowered IOP and medication use, and reduced the need for postoperative incisional glaucoma filtration surgery compared with CS alone after 5 years. The authors suggest that by reducing the dependency on topical medications and reducing the risk of further glaucoma surgery, HMS treatment has a durable and significant impact on the quality of life and the long-term consequences faced by patients with early-stage glaucoma.
Ahmed et al 2023	PY - The study provides a discussion of some limitations related to variability in measurements, the follow up period, and generalisability of the study. It addresses potential selection bias related to patient enrolment in the extension study. However, it does not explicitly discuss or address known confounders, secular trends, or discuss the use of data that was not originally collected to address the specific research question.	PY - The study provides clear conclusions supported by its findings and thoroughly discusses the relevance of its results to clinical practice. It compares its findings to other relevant studies and considers the clinical significance of ECD and ECL. The study does not explicitly address external validity or implications for policy.
Arnould et al 2024	Y - The study acknowledges the inherent limitations of real-world studies, including the lack of standardisation in treatment approaches and the potential for bias. The study notes that the population was primarily Caucasian with mostly primary open-angle glaucoma, which may limit the generalisability of the findings. Although the study used LOCF to address missing data, it does not explicitly state the extent of missing data for each variable. The study notes that the high rate of visual acuity loss reported may be due to methodological factors related to the registry design which captures data at every visit. The study does not specifically address secular trends.	Y - The study concludes that the Xen 45 gel stent implant offers acceptable efficacy for treating open-angle glaucoma but notes a significant rate of reoperation and needling. Outcomes were less effective when combined with cataract surgery. The authors discuss the limitations of generalising the findings to other ethnicities due to the population being mostly Caucasian. They note that their results are in line with other studies. The study suggests that the Xen 45 gel stent implant is an acceptable surgical approach for open-angle glaucoma. However, clinicians should be aware of the higher rates of reoperation and needling and the reduced effectiveness when combined with cataract surgery.
Bambo et al, 2024	PY - The study acknowledges key limitations related to its retrospective design, including potential for selection bias, known confounders, and missing data, which may impact the validity and generalisability of the results. The methods used to address these limitations are described, including statistical adjustment for confounders. However, the study does not discuss the implications of using data that may not have been initially collected for the specific research questions and there is no analysis of the potential for secular trends	Y - The study provides a good discussion of the interpretation of its results and the implications for clinical practice. The external validity of the study is discussed but not fully explored. However, the study does not address implications for health policy.
Clement et al 2020	PY - The study acknowledges limitations related to its retrospective design, lack of a control group, and variability in medication management. The study also provides information on the extent of missing data but does not explain methods for handling missing data. The discussion does not include any information on secular trends, or how they might affect the results	Y - The study provides a clear interpretation of results, considers the external validity of the findings based on its real-world design, and discusses how the results might inform clinical practice
Dang et al 2018	PY - The study acknowledges that the small sample size might have limited the ability to find risk factors related to Trabectome failure. The study found P=0.06 in terms of age >50, high cup/disk ratio and worse preoperative visual acuity, suggesting a high risk of Trabectome failure. The study notes that future research with larger samples should be done. * The study also points out that it did not specifically study the impact of Trabectome on advanced and refractory glaucoma, and that such studies are necessary. The study does not discuss secular trends or the implications of using data that was not originally collected for this research. The study does not explain how missing data was addressed	PY - The study concludes that Trabectome is an efficient and safe procedure for Chinese glaucoma patients in a 2-year follow-up. The study does not explicitly discuss implications for policy or practice.
Deitz et al 2023	Y - The retrospective design introduces potential bias. The study acknowledges that the nonrandomized nature of the study, without major exclusion criteria and no prior medication washout, represents a real-world scenario, leading to an imbalance in the severity of glaucoma between surgical groups, which is a source of potential bias. The study notes that the decision regarding which procedure a patient should undergo was made jointly by the surgeon and patient, which introduces potential bias. The study acknowledges that the use of the first-generation iStent may limit the generalizability of the results as it has been largely replaced by the second generation iStent inject. The study notes the relatively modest sample size of the phaco/iStent/ECP group.	Y - The study concludes that when iStent was added to phaco/ECP, the success rate was significantly higher at earlier postoperative visits compared to phaco/ECP alone, even when excluding cases of severe glaucoma. The study also mentions the need for prospective randomised controlled trials to determine if one combined MIGS procedure is superior to another
Ferguson et al 2020	PY - Study partially discusses known confounders or secular trends; only briefly mentions the implications of using data not originally collected for a specific research question. The discussion also references the known effect of cataract surgery alone on reducing IOP and suggests the microbypass stent is augmenting the effect.	PY - Study demonstrates evidence of conclusions and relevance, including a discussion of the overall interpretation of results and some discussion of external validity, but offers limited explicit discussion of implications for policy and practice
Gabbay et al 2021	Y - The study provides a comprehensive discussion of its limitations, including the retrospective design, potential biases related to selection and treatment, the influence of confounders, the impact of missing data, and the implications of using historical data. While the study does not explicitly discuss secular trends, it does address the potential challenges related to using data not originally collected for the specific research question.	Y - The overall interpretation is that the XEN45 gel stent is a viable, effective, and safe means of reducing IOP in patients with glaucoma at 36 months. The discussion includes the external validity of the findings, relating the demographic makeup of the cohort to local census data. Implications for practice are discussed, with the recommendation that patients should be advised of the risk of failure, need for bleb revisions, and postoperative topical treatment. The study also suggests that treatment should take account of ethnicity. It is suggested that XEN45 procedures and follow-up should be conducted in specialised glaucoma clinics rather than general ophthalmology settings, in light of the risk of bleb revisions

Hengerer et al 2022	PY - The study acknowledges that its data was originally collected to provide clinical care to patients. The data were analyzed retrospectively and for the purpose of a research study, but this was done in a prospective fashion. The study does not specifically discuss secular trends, but the study design with a long follow-up period should mitigate this. Although the missing data was minimal, the study does not discuss its potential impact or any methods used to address it.The study acknowledges that within the combined subgroup, the IOP-lowering effect of cataract extraction could not be entirely separated from the effect of the stent implantation.	Y - The study provides a detailed account of its methodology, results, and limitations, and it offers conclusions that are relevant to clinical practice. The study's strengths include its prospective design, long follow-up period, and real-world patient population. The study also highlights potential areas for further research.
Holmes et al 2022	Y - The study is transparent about the limitations associated with its design and data. It acknowledges potential biases, confounders, missing data and issues around interpretation of medication use, and addresses these issues through statistical methods and careful interpretation of the findings. It also makes it clear that the data was collected in a real-world setting and was not originally designed to answer this specific research question.	Y - Study emphasizes that both devices are safe and effective in reducing IOP and medication, while also acknowledging the modest level of reduction. The study's external validity is supported by the use of registry data, which increases the generalisability of the findings. The implications for practice are that surgeons can use either device with confidence, and that there may be a slight advantage with iStent inject in medication reduction, but this needs to be confirmed.
Inatani et al 2022	PY - The main limitation acknowledged is the lack of a control group of patients receiving phacoemulsification alone. The authors mention that this makes it difficult to determine whether the iStent is effective in controlling visual field impairment, but that the study is generalisable, as it reflects real world clinical practice. The study mentions the limited follow-up period of 2 years is a limitation, although for a post marketing study, two-year follow-up is substantial. The study does acknowledge some possible bias, due to the lack of a control group, but argues that the real-world clinical practice aspect enhances the generalisability of results. The study acknowledges the potential impact of missing data, although it notes that the reasons for missing data were mainly non-clinical. The study doesn't discuss implications of using data not originally collected for the specific research question as the study was designed for post-marketing surveillance. Secular trends are not specifically addressed.	Y - The study concludes that the iStent combined with phacoemulsification is a safe and effective treatment for reducing IOP and medication burden in Japanese patients with OAG. The authors highlight that these findings are consistent with previous international studies. The external validity of the study is supported by the inclusion of multiple sites, and the pragmatic nature of the study design which has the benefit of reflecting real world clinical practice.
Katz et al 2018	PY - The study includes a discussion of limitations, addressing sources of potential bias, known confounders, and missing data. However, it does not explicitly address secular trends.	Y - The study provides a clear interpretation of its results, concluding that standalone iStent implantation, particularly with multiple stents, is an effective and safe treatment option for patients with mild-to-moderate OAG. While acknowledging limitations that affect external validity, the study emphasises consistency with other clinical and laboratory work. The findings have implications for clinical practice, suggesting that multiple stents may be a more effective and durable treatment approach for some patients, and supporting the use of iStent as a minimally invasive standalone procedure for OAG.
Lee et al 2020	PY - The study discusses limitations related to the learning curve associated with new devices, noting the importance of proper stent placement and management of intraoperative bleeding. The study acknowledges that this is a retrospective case series from a single surgeon. It discusses the potential for bias related to variations in ocular anatomy and the challenge of controlling IOP following phacoemulsification in glaucoma patients. The study does not, however, explicitly discuss the implications of using data that was not originally collected for the specific research question and how this may introduce bias. The study notes missing data but does not discuss how this limitation might affect the results. It also does not address potential secular trends.	Y - The study concludes that combining phacoemulsification with a MIGS device reduces intraocular pressure and the number of medications in open-angle glaucoma patients, with longer-term effects at 2 years follow-up. The discussion interprets the results in light of previous studies. It notes that the results are from a real-world setting, which enhances the external validity. The study discusses the clinical relevance of the findings, particularly the potential to reduce eyedrop usage and ocular surface toxicity, as well as a protective effect against IOP spikes in advanced glaucoma patients. The authors highlight that careful training is important for new surgeons. The study notes the results may inform glaucoma management.
Mitchell et al 2024	Y - The authors acknowledge that using data from the IRIS registry, which was not specifically collected for this research, may have introduced bias. The study notes that limitations in data availability in the registry were addressed by the size of the data set and fully adjusted Cox models. The study addresses the potential for indication bias and the possibility that more severe glaucoma cases were more likely to receive specific treatments. Propensity score matching was used to mitigate this, but the authors note that it may not fully negate this bias. The study notes that the IRIS Registry does not capture glaucoma medication use.	Y - The main conclusion is that prior LTP may be associated with a higher chance of subsequent glaucoma surgery following ab-MIGS, with or without concurrent phacoemulsification. This suggests that patients who have undergone LTP may have less benefit from subsequent ab-MIGS procedures. The findings have implications for patient selection and managing expectations. The study suggests that those with prior LTP should be counselled that they may be at higher risk for requiring reoperation after ab-MIGS.
Montesano et al 2023	Y - The study discusses several limitations: masking, post-hoc analysis, missing data, follow-up time, generalisability, IOP measurements. The study uses a time-weighted average of IOP measurements that were not measured at the same time for all patients, which is a limitation in terms of the effect of IOP on VF progression. The study does discuss the implications of using data that was not originally collected to answer the specific research question, as it is a post-hoc analysis	Y - The study concludes that cataract surgery combined with Hydrus microstent (CS-HMS) significantly preserves visual field (VF) in glaucoma patients compared to cataract surgery alone (CS). The study acknowledges that its population consisted of patients with mild to moderate glaucoma undergoing cataract surgery. Therefore, the results might not generalize to individuals with advanced glaucoma, secondary open-angle glaucoma, or those receiving the Hydrus microstent (HMS) as a standalone procedure. The findings suggest that combining HMS with CS may offer a clinically meaningful benefit in preserving vision and reducing the risk of fast progression in glaucoma patients undergoing cataract surgery.
Oberfeld et al 2023	Y - The study discusses its limitations, addresses sources of potential bias and known confounders, considers missing data through the use of GEE models, and acknowledges the constraints of using retrospectively collected data	Y - The study provides a clear interpretation of its findings, acknowledging both the strengths and limitations of the research. The authors discuss the implications of their results for clinical practice, highlighting the potential benefits of combining multiple MIGS procedures, while recognising the need for more research to further validate their findings.
Oberfeld, Helwe et al 2023	Y - The study acknowledges that as a non-randomized, retrospective study, there may be differences between the Phaco/Hydrus and Phaco/KDB groups that were not measured or adjusted for, introducing potential bias. The study acknowledges attrition in both cohorts throughout the postoperative timepoints. The study notes that the results may not be generalizable to all populations due to the low baseline medication burden and normal range baseline IOP. The study acknowledges that the data was collected as part of routine clinical care, and there is no explicit discussion of the implications of using this data to answer the specific research question	Y - The study provides a discussion of the conclusions and relevance. The study concludes that both Phaco/Hydrus and Phaco/KDB result in significant and sustained reductions in IOP and medication burden for over 12 months. There were no significant differences between the two procedures in terms of IOP, medication use, survival, or procedure time.The authors note that the results may not be generalizable to populations with different baseline characteristics, such as higher medication burdens or higher baseline IOP. The study suggests that Phaco/KDB may be preferable, given similar efficacy and

		procedural time without the use of a permanent implant and associated long-term risks of complications
Reitsamer et al 2022	Y - The study includes a discussion of its limitations.<br>The retrospective nature of the chart review limited the availability of data across sites, due to differences in follow-up regimens.The anonymized nature of the data prevented querying of data once entered and precluded sub-analysis by site. The findings might not reflect current practices due to rapid evolution of surgical techniques and peri-operative practices.The study acknowledges the variable availability of data, and that only observed data was reported, without imputation of missing data.The study acknowledges the potential for bias due to the retrospective design.The study notes that it uses data collected as part of routine clinical care, which may not have been designed to answer the specific research questions.	Y - The study concludes that the gel stent, when implanted using the traditional ab-interno technique, provides consistent effectiveness in reducing IOP and the number of IOP-lowering medications over 3 years, with a predictable and acceptable safety profile. The study notes that its multicentre results are similar to those of prior single and multicentre studies, suggesting a degree of external validity, especially for European populations
Salimi et al 2021	Y - The study acknowledges that the data was not originally collected to answer the specific research question, as it was extracted from routine clinical practice, and this may introduce potential biases. Potential confounders are addressed through the use of regression models. The study mentions that a longer follow-up period, and studies with multiple surgeons and sites are needed	Y - The overall conclusion is that iStent inject trabecular micro-bypass stents, implanted during cataract surgery, are a safe and effective intervention for reducing IOP and medication burden in a variety of glaucoma subtypes and severities. The study suggests that the results can be used by doctors and patients to guide treatment decisions.
Samuelson et al 2019	PY - The study does acknowledge some limitations.It mentions that the surgeons were not masked to the treatment groups, which could introduce bias.<br>The study also notes that the data includes the surgeons' learning curve with the technology.The study does not specifically discuss missing data, secular trends, however, the study does report that some subjects did not complete the medication washout at 24 months, and includes analysis of an intent-to-treat cohort that includes all randomised subjects, even those who did not receive the intervention.	Y - The overall interpretation of results is that the iStent inject second-generation trabecular micro-bypass stents, when implanted in conjunction with cataract surgery, provide a statistically significant and clinically meaningful reduction in IOP compared to cataract surgery alone. This is evidenced by both the primary outcome of ≥20% reduction in DIOP and the secondary outcome of the mean reduction in DIOP at 24 months.<br>The study suggests that the iStent inject is a safe and effective treatment option for individuals with mild to moderate primary open-angle glaucoma (POAG) undergoing cataract surgery.<br>The study also highlights that the iStent inject offers a less compliance-dependent treatment modality that may help reduce the burden of ocular hypotensive medications.
Samuelson et al 2021	Y - The implications of using data not originally collected to answer the specific research question are discussed by acknowledging that this was an exploratory analysis and not pre-specified.	Y - The study concludes that the iStent inject® implantation with cataract surgery may provide greater vision-related QOL improvement compared to cataract surgery alone
Schlenker et al 2017	Y - The study discusses several limitations, addressing potential bias, confounders, and missing data. The study acknowledges it is underpowered for safety due to the low rate of complications and relatively short follow-up. The study mentions that the adjusted analysis cannot account for unmeasured confounders, such as compliance, obesity, smoking status, or other general health markers. The study acknowledges that surgical technique and post-operative management varied by site, which could contribute to the relative success of the procedures.The study acknowledges that its retrospective design and loss to follow-up may result in an underestimation or overestimation of the success rates.	Y - The study concludes that both ab interno gelatin microstent and trabeculectomy with MMC resulted in similar success rates and safety profiles. There were more post-surgical interventions in the trabeculectomy group and likely more needling in the microstent group. There may be slightly more complications in the trabeculectomy group, and slightly more reoperations in the microstent group.
Strzalkowska et al 2021	PY - The study acknowledges its retrospective nature and the potential biases inherent in such a design, addressing this through rigorous matching techniques. It also points out limitations related to data handling, the study population, and surgeon experience. However, the study does not discuss missing data or secular trends.	Y - The study provides a clear interpretation of its results, highlighting the different strengths and weaknesses of AIT and TRAB. It also addresses the external validity of its findings, and makes a case for the implications for policy and practice, suggesting appropriate use cases for each procedure.
Tojo et al 2020	Y - The study acknowledges several limitations, including: cutoff values, heterogeneity of glaucoma types, surgical methods, retrospective nature, lack of clear definitions, follow up period.	Y - Conclusions were that: trabectome surgery significantly reduced IOP.The mid-IOP group (18-26 mmHg) achieved the best surgical outcomes compared to the low and high IOP groups, thin CCT and simultaneous cataract surgery were associated with better surgical outcomes. No discssuon of external validity and implications for policy and practice were made.
Vukmirovic et al 2023	PY - There is no discussion of secular trends and implications of using data that not originally not collected to answer specific research question.	PY - The study concludes that gelatin stent surgery appears to have less IOP reduction effect compared to trabeculectomy at 2 years. The study acknowledges that its results may not be generalisable to populations with different ethnic backgrounds. The study also implies that the gelatin stent device technology has not reached maturity in terms of maximizing the benefits of biocompatibility and tissue flexibility. However, the study does not explicitly provide recommendations for policy or practice.
Yang et al 2023	Y - Addresses restrospective study design, exclusion criteria, missing data, surgeon proficiency, private vs tertiary academic centres and secular trends as all potential sources of bias. The study acknowledges that using data from the IRIS registry, which was not originally collected to answer this specific research question, introduces limitations	Y - The study concludes that MIGS procedures, when performed concurrently with phacoemulsification, have substantially lower reoperation rates compared to stand-alone MIGS procedures. The study's external validity is strengthened by the use of the IRIS Registry, a large, longitudinal dataset of ophthalmic patients representative of the United States population.The findings suggest that when feasible, performing MIGS concurrently with phacoemulsification may lead to better long-term outcomes in terms of reduced reoperation rates, particularly for ECP and goniotomy or canaloplasty.
