# Supplementary material for: Evaluating Long-Term Effectiveness and Safety of Glaucoma Surgical Innovations: A Systematic Review
Source: J Glaucoma. 2026 Oct 16;35(11):838–45. doi: 10.1097/IJG.0000000000002770 (PMC13722426; doi:10.1097/IJG.0000000000002770)
Supplement: Supplementary file 7 [file ijg-35-838-s007.pdf]

Supplementary Table 7. Data extraction as per IDEAL Stage 4 Framework for Other information (rated Yes, Partially Yes or No)

	Other information			
**Authors + Year**	**13. Please quote reference or DOI if a protocol was written in advance and made available (n/a if not available)**	**14. Was appropriate ethical approval obtained and informed consent process described. If consent for future use of data was not obtained, explanation provided?**	**15. Have sources of funding and support, role of funders, and other conflicts of interest been reported?**	**16. Are regulatory approvals being sought or obtained (e.g. CE Marking, FDA approval, etc)? Include the date of approval, if applicable**
Ahmed et al 2022	Y - Clinicaltrials.gov, NCT01539239	Y - the study protocol was approved by the governing Institutional Review Board or Ethics Committee at all participating centers and by national regulatory agencies where applicable. The study do not explicitly state whether consent for future use of data was obtained in this paper but was recieved in previous paper	Y - The study acknowledges that it was supported by Ivantis, Inc. The authors have made financial disclosures related to various companies, including Ivantis, and these are listed	N - The sources do not explicitly state that regulatory approvals are being sought or obtained for the device itself.
Ahmed et al 2023	Y - Clinicaltrials.gov, NCT00323284	Y - The study was approved prospectively by the Western Institutional Review Board. Informed consent through an IRB form collected for all included patients.	Y - The study was funded by Glaukos Corporation who participated in the trial, including the collection, management, and analysis of data, as well as the preparation of the manuscript.Support for manuscript preparation was provided by ApotheCom. Authors have various extensive connections.	Y - The study mentions that iStent inject has obtained FDA approval (does not mention date). No mention of CE marking.
Arnould et al 2024	n/a	Y - The study states that institutional approval was obtained from the Royal Australian and New Zealand College of Ophthalmologists Human Research Ethics Committee and the French Institutional Review Board. The study also confirms that participants gave informed consent to participate.	Y - The study reports that funding was supported by the Royal Australian and New Zealand College of Ophthalmologists. The authors declare that they have no competing interests.	Y - The study mentions that the Xen gel stent was FDA approved through the 510k pathway, which does not require a randomised controlled trial. The date of the FDA approval is not stated.
Bambo et al, 2024	n/a	Y - The study protocol was approved by the Ethic Committee of the Miguel Servet University Hospital (Protocol number: 23/2021).	Y - The study reports that AbbVie provided funding for medical writing and editorial support, but had no influence on the study's content. The authors have declared that they have no conflicts of interest.	Y - The XEN gel stent obtained the CE mark in December 2015.<br>The XEN gel stent was approved by the Food and Drug Administration (FDA) in November 2016.
Clement et al 2020	n/a	Y - The study received ethics committee approval from the Royal Australian and New Zealand College of Ophthalmology Human Research Ethics Committee. All patients signed an informed consent to allow for the retrospective evaluation of their de-identified clinical data	Y - Editorial assistance and publication processing charges were provided by Glaukos Corporation. The authors also provided a comprehensive disclosure of any potential conflicts of interest, including personal fees, consultancy roles, research grants and advisory board memberships	N - The study does not explicitly state whether regulatory approvals are being sought or obtained for the iStent inject device
Dang et al 2018	n/a	Y - The study was approved by the Institutional Review Board of Peking University Third Hospital. Due to the retrospective nature of the study, informed consent was not required.	Y - The study was supported by a grant from the National Natural Science Foundation of China. The authors explicitly state that there are no conflicts of interest.	Y - the study mentions that the Trabectome is a United States Food and Drug Administration (FDA)-approved device. The date of the approval is not provided in the study.
Deitz et al 2023	n/a	Y - The study states that approval for the retrospective review was obtained from the University of Colorado Institutional Review Board (COMIRB# 16-1345). There is no mention of informed consent in this study since it was a retrospective chart review.	Y - The study was supported by a grant to the Department of Ophthalmology from Research to Prevent Blindness, Inc. and the Frederic C. Hamilton Macular Degeneration Center and the Colorado Clinical & Translational Sciences Institute (CCSTI). The authors state that the funding organisations had no role in the design or conduct of this research.	N - The study does not mention regulatory approvals being sought or obtained.
Ferguson et al 2020	n/a	Y - Approved by University of South Dakota Institutional Review Board; no informed consent necessary as all data was anonymised	Y - Investigator-initiated trial grant from Glaukos Corp; he specific role of Glaukos Corp. in the study design, data collection, analysis, or interpretation is not detailed in the text, other than providing funding	Y - The study mentions that the trabecular microbypass stent (iStent) was the first FDA-approved MIGS device, and was introduced in 2012. Does not discuss CE marking.
Gabbay et al 2021	n/a	Y - Study approved by The Southend University Hospital Audit Committee. However, there is no specific mention of informed consent for use of data for research purposes, likely because this was a retrospective audit of clinical data.	Y - The authors declared no potential conflicts of interest. The authors received no financial support for the research.	N - Study does not inform in re: CE marking or FDA approval but implies device has CE mark due to its use within European centres
Hengerer et al 2022	n/a	Y - The study was approved by the institutional review board of the University of Heidelberg. All participants provided informed consent prior to enrolment.	Y - The study acknowledges that an unrestricted research grant and editorial assistance were provided by Glaukos Corporation. The authors disclose financial relationships with Glaukos Corporation	Y - The study states that iStent trabecular micro-bypass was the first US Food and Drug Administration-approved device, in 2012. The study mentions that the newest iStent inject model, the wide-flanged iStent inject W, was released in Germany in 2020.
Holmes et al 2022	n/a	Y - Institutional ethics approval was obtained from the Human Research Ethics Committee of the Royal Australian and New Zealand College of Ophthalmologists. The Fight Glaucoma	Y - An unrestricted educational grant was provided by Glaukos for the manuscript. No other conflicts of interest.	Y - Mentions FDA pivotal trials, no date given for approvals

		Blindness (FGB) database has ongoing Human Research Ethics Committee (HREC) ethics approval		
Inatani et al 2022	n/a	Y - The study was conducted as part of mandatory actions for approval of iStent in Japan and complied with the Japanese ministerial ordinance on Good Post-marketing Study Practice. According to this ordinance, ethical approval of the participating medical institutions and patient consent was not required	Y - The study was sponsored by Glaukos Corporation, which also funded the journal's Rapid Service and Open Access Fees. The role of medical writers who helped with the publication of the manuscript, who were also funded by Glaukos Corporation, is also mentioned.	Y - The study was conducted as part of the mandatory actions for approval of iStent in Japan. The study does not explicitly state whether specific regulatory approvals (CE Marking, FDA approval) were being sought or obtained. The study does not provide a date of approval, but does mention that the iStent had been available in Japan for the treatment of OAG combined with cataract surgery since 2016.
Katz et al 2018	Y - ClinicalTrials.gov ID NCT 01517477	Y - The study explicitly states that the Armenian Ministry of Health provided Ethics Committee approval. The study confirms that all participating subjects provided written informed consent.	Y - The study was sponsored by Glaukos Corporation. They were involved in providing the study devices, financial backing, data management, and editorial support. There are various connections between several authors and companies.	Y - Although the study confirms that the iStent devices have received FDA approval, it does not provide the specific dates of approval or mention whether other regulatory approvals, such as CE Marking, are being sought or have been obtained.
Lee et al 2020	n/a	Y - Institutional ethical approval for the trial was obtained through the University of Queensland (Number 2016-05-440). All study procedures adhered to the Declaration of Helsinki for research involving humans and written informed consent was obtained from the study participants.	Y - The study states that "The Hydrus stent was supplied by Ivantis, Irvine, CA"	N - There is no mention of regulatory approvals being sought or obtained in the provided document.
Mitchell et al 2024	n/a	Y - Institutional review board/ethics committee ruled that approval was not required for this study because the data was deidentified. Informed consent was not required because the data stored within the IRIS Registry is deidentified	Y - Some authors disclosed grants, royalties, payments, patents, leadership roles, and stock options.<br>Other authors declared support or consultant roles. There was no funding for this study, so there was no role of funders to describe	N - The study does not mention any regulatory approvals being sought or obtained
Montesano et al 2023	Y - Clinicaltrials.gov, NCT01539239	Y - The study states that the protocol was approved by local governing institutional review boards, ethics committees, and national regulatory agencies. All participants provided written informed consent before any procedure	Y - The study was financially supported by Ivantis, Inc., an Alcon company.<br>Some authors reported financial disclosures for Alcon, Inc.. The authors state that they meet the current ICMJE criteria for authorship.	N - The study does not explicitly mention whether regulatory approvals (e.g., CE Marking, FDA approval) were sought or obtained, and no dates of approval are provided
Oberfeld et al 2023	n/a	Y - The study protocol and waiver of consent for retrospective chart review were approved by the Massachusetts General Brigham Institutional Review Board. A waiver of consent was granted for the retrospective chart review.	Y - The funding was provided through donations, with no specific roles defined for the funders beyond financial support, and a single conflict of interest was disclosed relating to one author's role as a speaker for a pharmaceutical company	N - Does not include any information on regulatory approvals, such as CE Marking or FDA approval
Oberfeld, Helwe et al 2023	n/a	Y - The study states that the study protocol was approved by the Massachusetts General Brigham Institutional Review Board. However, the study does not describe the informed consent process	Y - No research grants received. Philanthropic support from public received.	N - The study does not mention any regulatory approvals being sought or obtained for the devices or procedures being studied.
Reitsamer et al 2022	n/a	Y - The study states that the protocol was approved by an independent ethics committee at each site, as required by local regulations. The need for written patient consent was waived by all ethics committees due to the retrospective design, anonymized data, and low risk of confidentiality breach, per the European General Data Protection Regulation.	Y - Writing and editorial assistance was provided by Evidence Scientific Solutions and funded by Allergan (an AbbVie company). Project and data management, statistical analysis and development of the study report were provided by ICON plc, Dublin, Ireland. Authors declared some conflicts of interests.	N - The study does not explicitly mention the dates or status of specific regulatory approvals such as CE Marking or FDA approval.
Salimi et al 2021	n/a	Y - The study was approved by the Ethics Committee of the Hôpital Maisonneuve-Rosemont in Montreal, QC, Canada. The study does not explicitly describe the informed consent process. However, it is implied that consent was obtained, given the ethical approval was provided. The study does not specify whether consent for future use of data was obtained.	Y - The study states that the authors have no sources of funding to declare. Some authors are consultants for companies.	Y - The study mentions that the iStent inject was approved for the treatment of mild-to-moderate primary open-angle glaucoma (POAG), without specifying date of approval
Samuelson et al 2019	Y - ClinicalTrials.gov ID NCT00323284	Y - It was conducted with Institutional Review Board (IRB) approval, and study procedures followed the tenets of the Declaration of Helsinki (2008), including written informed consent of all participating subjects	Y - The study was funded by Glaukos Corporation, which also participated in the design and conduct of the study, data collection and analysis, and manuscript preparation.The authors list their financial disclosures including support, grants, consultation fees and employment from Glaukos, Alcon, Allergan, and other pharmaceutical and medical device companies	Y - The iStent inject received CE Mark approval in 2011, and was also approved by the U.S. Food and Drug Administration (FDA). The article does not include the exact date of the FDA approval but states that the study was the pivotal study supporting the recent FDA approval
Samuelson et al 2021	Y - Clinicaltrials.gov, NCT00323284	Y - The study was conducted with Institutional Review Board ap-proval, and study procedures followed the tenets of the Declaration of Helsinki (2008), including written informed consent of all participating subjects.	Y - The study was sponsored by Glaukos Corp.The authors disclose their financial relationships with Glaukos, as well as other companies	Y - The study mentions that the pivotal study of cataract surgery plus iStent inject® led to the FDA's approval of iStent inject® in 2018
Schlenker et al 2017	n/a	Y - The study states that institutional review board approval was obtained from each site. However, the study does not explicitly	Y - The study states that no funding was obtained. The authors also included a financial disclosure section, stating that some authors	N -The study does not mention any regulatory approvals being sought or obtained.

		describe the informed consent process, or whether consent for future use of data was obtained, or whether an explanation was provided.	received honoraria or consultancy fees from Allergan and other companies	
Strzalkowska et al 2021	n/a	Y - The study protocol was approved by the local ethics committee of the University of Würzburg, Germany.<br>The study states that informed consent was waived because of its retrospective nature	Y - The study states that it received open access funding enabled and organised by Projekt DEAL. This indicates that the publication costs of the study were covered by this organisation, but it does not necessarily mean that they provided funds for the study itself.	Y - The sources provide evidence that the trabectome device used in AIT was subject to FDA post-market surveillance requirements, implying that some regulatory approval had been granted at an earlier point. However, the sources do not specify whether any regulatory approvals are currently being sought or provide a specific date of approval.
Tojo et al 2020	n/a	Y - Research protocol was approved by the Institutional Review Board of the University of Toyama.	Y - No funding or conlficts or interests	N - The study does not explicitly mention any regulatory approvals being sought or obtained
Vukmirovic et al 2023	n/a	Y - received approvals from the Human Ethics committee of the University Of Western Australia	Y - DY, YU and WH Morgan are inventors of original crosslinked gelatine microfistula technology. WH Morgan has acted as an advisor for Allergan. WH Morgan has received a grant from Allergan to study medium term results of XEN45.	N - The study does not state whether regulatory approvals are being sought or have been obtained.
Yang et al 2023	n/a	Y - Institutional review board approval was not required for this study because it does not constitute human subjects research. The study used de-identified data from the American Academy of Ophthalmology's IRIS Registry, which is compliant with the Health Insurance Portability and Accountability Act (HIPAA). All research adhered to the tenets of the Declaration of Helsinki.	Y - Study was supported with grants from several institutions. The funding organisations had no role in the design or conduct of this research. Some authors had consultancy and other conflicts of interest.	Y - Mentions Xen Gel stent is FDA approved. Does not provide information in re: other MIGS procedures.
