# Supplementary material for: Evaluating Long-Term Effectiveness and Safety of Glaucoma Surgical Innovations: A Systematic Review
Source: J Glaucoma. 2026 Oct 16;35(11):838–45. doi: 10.1097/IJG.0000000000002770 (PMC13722426; doi:10.1097/IJG.0000000000002770)
Supplement: Supplementary file 8 [file ijg-35-838-s008.pdf]

Supplementary Table 8. Post-operative complications in the study and abstract (highlighted in red)

Authors + Year	Timing of follow up	If specific long term post-operative complications are mentioned in study, what are these?	Abstract
Ahmed et al 2022	5 years	The only long-term findings uniquely related to the device were focal peripheral anterior synechiae (PAS) or tissue adhesions near the trabecular entry point. The percentage of patients with PAS in the CS group was 3.7% and in the HMS group 14.6%. The study reports that there were no late observations of inflammation, corneal edema, or a need for device removal. Other adverse events were monitored, such as loss of BCVA, and visual field mean deviation, but these were not specific to the device itself. The study noted a modest but significant decline in endothelial cell density in both groups from 3 months to 5 years	Purpose: To present the 5-year results of the HORIZON trial comparing cataract surgery (CS) combined with an intracanalicular microstent with CS alone. Design: Prospective, multicenter, controlled randomized clinical trial. Participants: Patients with cataract and primary open-angle glaucoma treated with 1 or more glaucoma medications, washed-out diurnal intraocular pressure (DIOP) of 22 to 34 mmHg, and no prior incisional glaucoma surgery. Methods: Eyes were randomized 2:1 to receive a Hydrus Microstent (HMS; Ivantis, Inc) or no stent after successful CS. Main Outcome Measures: Intraocular pressure (IOP), glaucoma medication use, repeat glaucoma surgery, visual acuity, visual field, procedure-related adverse events, and corneal endothelial cell counts. Results: Three hundred sixty-nine eyes were randomized to HMS treatment, and 187 eyes were randomized to CS only. Study groups were well matched for preoperative IOP, medication use, washed-out DIOP, and glaucoma severity. Five-year follow-up was completed in 80% of patients. At 5 years, the HMS group included a higher proportion of eyes with IOP of 18 mmHg or less without medications than the CS group (49.5% vs. 33.8%; P¼0.003), as well as a greater likelihood of IOP reduction of 20% or more without medications than the CS group (54.2% vs. 32.8%; P < 0.001). The number of glaucoma medications was 0.5 0.9 in the HMS group and 0.9 0.9 in the CS group (P < 0.001), and 66% of eyes in the HMS group were medication free compared with 46% in the CS group (P < 0.001). The cumulative risk of incisional glaucoma surgery was lower in the HMS group (2.4% vs. 6.2%; P ¼ 0.027, log-rank test). No clinical or statistically significant differences were found in the rate of endothelial cell loss from 3 to 60 months between the HMS and CS alone groups (P ¼ 0.261). Conclusions: The addition of a Schlemm's canal microstent in conjunction with CS was safe, resulted in lowered IOP and medication use, and reduced the need for postoperative incisional glaucoma filtration surgery compared with CS after 5 years. Long-term presence of the implant did not affect the corneal endothelium adversely.
Ahmed et al 2023	5 years	The study found no device-related adverse events or complications were reported through 60 months. No additional adverse effects to corneal endothelial health compared to phacoemulsification was found.	Purpose: To report 5-year postoperative safety data of iStent inject , including overall stability, endothelial cell density (ECD), and endothelial cell loss (ECL) in patients with mild-to-moderate primary open-angle glau- coma (POAG). •DESIGN: 5-year follow-up safety study of the prospec- tive, randomized, single-masked, concurrently controlled, multicenter iStent inject pivotal trial. Methods: In this 5-year follow-up safety study of the 2-year iStent inject pivotal randomized controlled trial, patients receiving iStent inject placement and phacoemul- sification or phacoemulsification alone were studied for the incidence of clinically relevant complications associ- ated with iStent inject placement and stability. Corneal endothelial endpoints were mean change in ECD from screening and proportion of patients with > 30% ECL from screening, from analysis of central specular endothe- lial images by a central image analysis reading center at several time points through 60 months postoperatively. Results: Of the 505 original randomized patients, 227 elected to participate (iStent inject and phacoemulsification group, n = 178; phacoemulsification-alone control group, n = 49). No specific device-related adverse events or complications were reported through month 60. No significant differences were observed in mean ECD, mean percentage change in ECD, or proportion of eyes with > 30% ECL between the iStent inject and control groups at any time point; mean percentage decrease in ECD at 60 months was 14.3% ±13.4% in the iStent<br>inject group and 14.8% ±10.3% in the control group (P = .8112). The annualized rate of ECD change from 3 to 60 months was neither clinically nor statistically significant between groups. Conclusions: Implantation of iStent inject during phacoemulsification in patients with mild- to- moderate POAG did not produce any device- related complications or ECD safety concerns com- pared to phacoemulsification alone through 60 months.
Arnould et al 2024	2 years	Hypotony: Numerical hypotony (low IOP) was observed, with late hypotony rates (12-24 months post-surgery) at 1.5%. Symptomatic hypotony (low IOP with visual acuity loss) was observed, with late rates at 1.2%. Visual acuity loss: A loss of ≥10 letters of visual acuity from baseline was observed. Exposure of the glaucoma device: This occurred in 1.8% of cases. Secondary glaucoma procedures: These were required in 18% of eyes, including bleb revision and trabeculectomy. Bleb needling: This was performed in 28.4% of cases	Objective: To evaluate efficacy and safety outcomes of the Xen 45 gel stent implant over 24 months of follow-up.<br>Methods: A retrospective analysis of prospectively collected data from the Fight Glaucoma Blindness observational registry. Complete success (CS) was defined as intraocular pressure (IOP) reduction ≥20% from preoperative and an IOP ≤18 mm Hg and ≥6 mm Hg with no secondary procedure at 2 years and without IOP-lowering medications. Qualified success (QS) was defined similarly, allowing the use of IOP-lowering medications.<br>Results: The Xen 45 gel stent implant was implanted in 646 eyes of 515 patients. Preoperative IOP was 21.4±7.6 (mean±SD) mm Hg on 2.7±1.3 IOP-lowering medication and mean deviation was −10.2±8.4 dB. After 24-month follow-up, IOP was 16.8±7.3 mm Hg (mean reduction of 21.7%) on 1.2±1.4 IOP-lowering medications. CS and QS rates at 24 months were 26% and 48%, respectively. CS and QS were higher in the Xen stand-alone group (33% and 52%, respectively) than in the Xen+cataract group (16% and 42%, respectively). Bleb needling was performed in 28.4% of cases, and 18% underwent a secondary procedure.<br>Conclusions: The Xen 45 gel stent implant offers acceptable long-term efficacy for the treatment of open-angle glaucoma. However, there is a significant rate of reoperation and needling, and outcomes are less effective if combined with cataract surgery.
Bambo et al, 2024	4 years	7.3% of eyes (15 out of 206) underwent a needling procedure due to fibrosis or encapsulation of the bleb that did not respond to massage and topical hypotensive medications.. 17.5% of eyes (36 out of 206) underwent bleb revision. The rate of bleb revisions was significantly greater in the XEN+Phaco group compared to the XEN-alone group (29 vs 7 eyes respectively). 1.9% of eyes (4 out of 206) underwent a device replacement. 12.1% of eyes (25 out of 206) required additional glaucoma surgeries, such as trabeculectomy or glaucoma drainage devices. There was not a significant difference between groups, with 12 eyes in the XEN-alone group and 13 eyes in the XEN+Phaco group. Although mostly a short term	Introduction: The main purpose of the current study was to evaluate the long-term effectiveness and safety of XEN45 implant, either alone or in combination with cataract surgery, in patients with glaucoma. Methods: Retrospective and single center study conducted on consecutive patients who underwent a XEN45 implant, either alone or in combination with cataract surgery, between November 2016 and October 2021. The primary endpoint was the mean intraocular pressure (IOP) lowering from preoperative values. Results: Among the 230 screened patients, 206 eyes (176 patients) were included. Fifty-three (25.7%) eyes had undergone XEN alone and 153 (74.3%) eyes had undergone a combined procedure (XEN+phacoemulsification). The mean preoperative IOP was significantly higher in the XEN-alone (22.2 ± 5.9 mm Hg) than in the XEN+Phaco (19.8 ± 4.5 mm Hg) group (p = 0.0035). In the overall study population, the mean preoperative IOP was significantly lowered from 20.5 ± 5.0 mm Hg to 15.8 ± 4.4 at year-4, p < 0.0001. The mean preoperative (95% confidence interval) IOP was significantly lowered from 22.2 (20.6–23.8) mm Hg and 19.8 (19.1–20.6) mm Hg to 15.6 (12.2–16.9) mm Hg and 15.9 (15.2–16.5) mm Hg at year-4 in the XEN-alone and XEN+Phaco groups, respectively (p < 0.0001 each, respectively). The number of ocular hypotensive medications was significant reduced from 2.6 ± 1.0 drugs to 1.3 ± 1.3 drugs, with no significant differences between XEN-alone and XEN+Phaco groups (p = 0.1671). On the first

		complication, a transient subclinical hypotony was observed in 28 (13.6%) eyes. Post-operative IOP spikes (IOP >30 mm Hg) were observed in 12 (5.8%) eyes. No complication extended beyond month 6.	postoperative day, 62 (30.1%) eyes presented some type of complication. Fifteen (7.3%) eyes underwent a needling procedure. Conclusion: XEN45, either alone or in combination with phacoemulsification, significantly lowered the IOP and reduced the need of ocular hypotensive medication in the long-term.
Clement et al 2020	2 years	The study reports that no adverse events occurred at a rate of 2% or greater over the two years of follow-up. The few adverse events that did occur were mild, transient, and resolved with minimal to no intervention without any long-term problems. The nature of these specific adverse events is not specified in the study.	Purpose: This multicenter study evaluated 2-year effectiveness and safety following implantation of two second-generation trabecular micro-bypass stents (iStent inject®) with phacoemulsification. Materials and Methods: This was a retrospective study of iStent inject implantation with phacoemulsification by nine surgeons across Australia. Eyes had mild to advanced glaucoma (predominantly primary open-angle/POAG, appositional angle-closure/ACG, or normal-tension /NTG) or ocular hypertension (OHT), and cataract. Evaluations included intraocular pressure (IOP); medications; proportions of eyes with 0 or ≥2 medications, reduced/stable medications versus preoperative, and IOP ≤15 mmHg; visual acuity; cup-to-disc ratio (CDR); visual fields (VF); adverse events; and secondary surgery. Results: A total of 340 eyes underwent surgery and had 24-month follow-up data. At 24 months, mean IOP decreased by 16% from 16.4±4.7 mmHg preoperatively to 13.7±3.1 mmHg (p<0.001), and 77% of eyes achieved IOP of ≤15 mmHg versus 49% preoperatively (p<0.001). Mean number of medications decreased by 67% to 0.49±0.95 versus 1.49±1.20 preoperatively (p<0.001), with 74% of eyes medication-free versus 25% preoperatively (p<0.001), and 14% of eyes on ≥2 medications versus 46% preoperatively (p<0.001). Medication burden was reduced or stable in 98% of eyes versus preoperative. Stratified analyses showed significant IOP and medication reductions across glaucoma subtypes (POAG, ACG, NTG, OHT): 13–22% for IOP (p<0.01 for all) and 62–100% for medication (p<0.001 for all). Favorable safety included few adverse events; stable CDR, VF, and visual acuity; and filtering surgery in only 8 eyes (2.4%) over 2 years. Conclusion: This 340-eye multicenter dataset provides robust evidence of the safety and efficacy of iStent inject implantation with phacoemulsification, with significant and sustained IOP and medication reductions through 2 years. Results were similarly favorable across glaucoma subtypes (including POAG, ACG, NTG, OHT) and were attained across various glaucoma severities, clinical sites, and surgeons, highlighting the real-world versatility and utility of this treatment modality.
Dang et al 2018	2 years	The study does not report any long-term vision-threatening complications. The only reported complication was transient blood reflux in all cases, which resolved spontaneously within a few days. No sustained hypotony, aqueous misdirection, endophthalmitis, wound leakage, or choroidal hemorrhage was reported.Ten patients required secondary glaucoma surgery	Background: The aim of the study was to evaluate the long-term safety and efficacy of the Trabectome for Chinese glaucoma patients.Methods: This was a multicenter, retrospective, observational study. Glaucoma patients, except those with neovascular glaucoma, with/without a visually significant cataract were enrolled. The patients received Trabectome or a combined surgery with phacoemulsification and intraocular lens implantation. The primary outcome evaluation was a reduction in intraocular pressure (IOP), and the secondary outcomes were a reduction in glaucoma medication, the 2-year success percentages, and complications. Success was defined as an IOP <21 mmHg and at least a 20% IOP reduction from baseline after 3 months for any two consecutive visits, without additional glaucoma surgery. The data were processed using the R Stats Package version 3.0.0. The Wilcoxon test was used to compare the postoperative IOP and the number of glaucoma medications with baselines. The Kaplan–Meier test was used to calculate the 2-year success percentage. The risk factors related to Trabectome failure were determined by logistic regression. Results: A total of 120 glaucoma patients were enrolled. The Trabectome efficiently reduced the IOP from a baseline of 22.8 ± 1.34 mmHg to 17.6 ± 0.96 mmHg, and the use of glaucoma medications from 2.2 ± 0.17 mmHg to 1.4 ± 0.21 in a 2-year follow-up (both, P < 0.01). The overall success percentage was 80%. No risk factor related to Trabectome failure was identified. No vision-threatening complication was observed. Ten patients, who required secondary glaucoma surgery, all reached the target IOP.<br>Conclusions:In a 2-year follow-up, Trabectome was an efficient and safe procedure for Chinese glaucoma patients.
Deitz et al 2023	2 years	To evaluate the safety of the procedure, the need for additional surgery, and postoperative complications were noted. These complications included: postoperative endophthalmitis, postoperative rebound inflammation requiring treatment, and intraoperative anterior vitrectomy. Complications occurred in four patients who underwent phaco/ECP, two who underwent phaco/iStent/ECP, and one who underwent phaco/iStent.	Introduction: To compare outcomes of phacoemulsification combined with endoscopic cyclophotocoagulation (phaco/ECP), first generation iStent implantation (phaco/iStent), or both (phaco/iStent/ECP) in patients with open-angle glaucoma.<br>Methods: A retrospective chart review was performed on patients at the University of Colorado Department of Ophthalmology. Outcomes included intraocular pressure (IOP), medication use, best corrected visual acuity (BCVA), and surgical complications were analyzed. Success was defined as IOP reduction of ≥ 20% and/or reduction by at least one glaucoma medication.<br>Results: A total of 394 eyes were included in the study. There were 170 eyes (43.1%) in the phaco/ECP group, 175 eyes (44.4%) in the phaco/iStent group, and 49 eyes (12.4%) in the phaco/iStent/ECP group. The mean pre-operative IOP was 15.9 mmHg for phaco/ECP, 15.8 mmHg for phaco/iStent, and 15.2 mmHg for phaco/iStent/ECP. At 24 months, the mean IOP was 13.7 mmHg (p < 0.0001), 14.2 mmHg (p = 0.0001), and 13.0 mmHg (p = 0.0007), respectively. The mean pre-operative number of glaucoma medications was 2.0 for phaco/ECP, 1.4 for phaco/iStent, and 2.2 for phaco/iStent/ECP and at 24 months post-surgery decreased to, 1.8 (p = 0.011), 0.9 (p < 0.0001), and 1.7 (p = 0.01), respectively. The success rate at 24 months was 54.4% for phaco/ECP, 75.3% for phaco/iStent, and 55.6% for phaco/iStent/ECP.<br>Conclusion: Phacoemulsification when combined with ECP, iStent, or both, lowered IOP and glaucoma medication reliance at 24 months. The success rate for phaco/iStent was significantly higher than phaco/ECP. When iStent was added to phaco/ECP, the success rate was higher at earlier postoperative visits compared to the phaco/ECP alone.
Ferguson et al 2020	6 years	The study explicitly states that "no patients had sight-threatening complications including no hypotony, maculopathy, retinal detachment, or endophthalmitis". Furthermore, there were "no reports of stent obstruction, hypotony, or inflammatory sequelae such as peripheral anterior synechiae". This suggests that the iStent device did not cause specific long-term complications in the eyes of the study participants. The study notes that the "long-term safety profile observed through 6 years was not markedly dissimilar than what was demonstrated through the first 24 months"	Purpose: To investigate the long-term safety and efficacy of an iStent trabecular microbypass stent in combination with cataract surgery in eyes with primary open-angle glaucoma (POAG). Setting: Private practice; Sioux Falls, South Dakota. Design: Retrospective, consecutive case series. Methods: This case series included eyes implanted with a single trabecular microbypass stent in combination with phacoemulsification in patients with mild to severe POAG. Data were collected preoperatively and at day 1, week 1, month 1, and up to 6 years postoperatively. Primary outcome measures included mean intraocular pressure (IOP) and number of glaucoma medications. Safety was noted by assessing the incidence of IOP spikes and need for additional surgery. Results: The study comprised 411 eyes. Mean IOP was reduced to 14.9±4.2 mmHg compared to 18.8±5.6 mmHg at baseline at 6 years postoperative. The mean number of medications was reduced to 1.2±1.0 from 1.4±1.1 at baseline. In eyes with severe stage of disease, there was a mean IOP reduction >6 mmHg at 6 years postoperative. Eyes with baseline IOP ≥18 mmHg achieved a more robust reduction in IOP. Fifteen eyes underwent a secondary glaucoma procedure. There were no intra- or postoperative complications. Conclusion: Trabecular microbypass stent implantation in combination with cataract surgery provides a sustained IOP reduction in eyes with mild-to-severe POAG. The degree of IOP reduction was more significant in eyes with higher baseline IOP and severe stage of disease.

Gabbay et al 2021	3 years	Specific long-term post-operative complications mentioned in the study include:<br>Need for needling (36.6% of patients); Failure requiring further surgical glaucoma procedures or stent removal (25% at 36 months); Conjunctival erosion or a positive Seidel sign leading to implant removal or revision (10 patients); Hypotony <6 mmHg (10% of patients at 1 day or 1 week postoperatively); IOP >30 mmHg (3% at the initial post-operative examination, 8% during the remainder of follow-up). Other adverse events: hyphema (3%), choroidal detachment (4%), corneal edema (3%), and shallow anterior chamber (9%), all of which resolved spontaneously.<br>Macular edema and vein occlusion (0.5% at 6 month follow-up).	Purpose: To report efficacy and safety measures of the XEN45 gel stent at 36 months in the UK National Health Service setting. Methods: Retrospective non-comparative audit of the records of patients who underwent XEN45 implantation between June 2015 and May 2017 was performed. Main outcome measures were intraocular pressure and number of antihypertensive medications used. Failure was defined as need for further surgery or stent removal. Success was defined as a 20% decrease in intraocular pressure without the need for additional glaucoma medications or a reduction in antihypertensive medications without an increase in baseline intraocular pressure. Needling rates and preoperative factors effect were assessed.<br>Results: The cohort included 205 patients (205 eyes) with primary open angle glaucoma (84.4%), angle closure glaucoma (7.8%), or refractory glaucoma (7.8%), 62.9% had a stand-alone procedure and 37.1%, a combined phaco-XEN45 procedure. Mean intraocular pressure was 22.6 ± 7.0 mmHg at baseline compared to 14.7 ± 3.8 mmHg at 24 months and 14.0 ± 2.9 mmHg at 36 months (p < 0.001 for both). Mean number of medications used was 2.6 ± 1.1 at baseline compared to 0.5 ± 0.9 and 0.6 ± 1.0, at 24- and 36-months, respectively (p < 0.001 for both). The failure rate at 36 months was 25%. Needling was required in 36.6%. Evaluation of background factors yielded an increased failure rate in non-Caucasians compared to Caucasians (74% vs 21%, p < 0.001). Conclusion: XEN45 implantation is effective and safe at 36 months' follow-up. Patients should be advised regarding the risk of failure and possible need for bleb revisions. Careful patient selection may be required.
Hengerer et al 2022	5 years	One case of mild intraoperative hyphema occurred in a standalone procedure, which resolved without intervention or sequelae by Week 1. The study reports that no new adverse events occurred during the additional two years of follow-up. There were no stent-related complications throughout the 5-year follow-up. Specifically, there were no signs of chronic inflammation such as peripheral anterior synechiae (PAS), and no cases of hypotony, endophthalmitis, corneal compromise, stent obstruction, myopic shift, or choroidal detachment	Introduction: This study evaluated the 5-year effectiveness and safety of iStent inject® trabecular micro-bypass with or without cataract surgery (Combined or Standalone, respectively) in patients with open-angle glaucoma (OAG). Methods:<br>This prospective longitudinal case series included consecutive iStent inject cases from a single surgeon at a large German academic hospital. Intraocular pressure (IOP), medications, safety, and indicators of disease stability through 5 years were assessed in the Overall cohort and in subgroup analyses stratified by usage (Combined or Standalone). Results: Preoperative mean IOP in the Overall cohort (n = 125) was 23.5 ± 6.2 mmHg on 2.68 ± 1.02 mean medications, reducing to 14.1 ± 1.8 mmHg on 0.77 ± 0.82 medications at 5 years (40% and 71% reductions, respectively; both p < 0.001). All but 1 eye (> 99%) were on medication(s) preoperatively, but 46% were medication-free at 5 years (p < 0.001). In Combined eyes (n = 81), mean IOP decreased by 39% (22.6 mmHg to 13.8 mmHg, p < 0.001) and medications by 69% (2.52 to 0.78, p < 0.001). In Standalone eyes, mean IOP reduced by 42% (25.3 mmHg to 14.6 mmHg, p < 0.001) and medications by 75% (2.98 to 0.74, p < 0.001). At final follow-up, 83% of eyes had achieved ≥ 20% IOP reduction, and all but 1 eye (> 99%) had the same or lower IOP versus preoperative; all eyes (100%) maintained or reduced their medication burden versus preoperative. Favorable safety included 0 intraoperative complications and 0 filtration surgeries through 5 years. Long-term indicators of disease stability (visual fields, retinal nerve-fiber layer thickness, and cup:disc ratio) were unchanged over the course of 5-year follow-up. Conclusions: iStent inject produced significant and durable 5-year reductions in IOP (nearly 10-mmHg reduction) and medications (nearly 2-medication reduction), with stable disease parameters over time. Combined and Standalone subgroups had similar outcomes.
Holmes et al 2022	2 years	The study reported low numbers of device malposition in both groups, with no statistically significant difference. There were no cases of endophthalmitis or clinically significant anterior uveitis in either group.	Background: To compare real-world 24-month outcomes of phacoemulsification combined with either iStent inject or Hydrus Microstent. Methods: Analysis of data from the Fight Glaucoma Blindness (FGB) international registry. Anonymized data from 344 eyes with mild-to-moderate open-angle glaucoma, normal-tension glaucoma or ocular hypertension that underwent phacoemulsification combined with either iStent inject (224) or Hydrus Microstent (120) were included. Data were adjusted for baseline characteristics using linear regression and propensity score matching. The primary endpoint was a comparison of mean intraocular pressure (IOP) at 24 months. Results: At 24 months, there was no significant difference in IOP reduction between the two groups, consistent across all analyses. The matched cohort showed iStent inject achieved 3.1 mmHg reduction and Hydrus a 2.3 mmHg reduction (p = 0.530) and a mean medication reduction of 1.0 for iStent inject versus 0.5 for Hydrus (p = 0.081). 5.4% of eyes in the iStent inject group and 7.5% of eyes in the Hydrus group required subsequent procedures to improve IOP control within 24 months. Complications were rare with no significant differences between the groups.<br>Conclusions: Twenty-four-month outcomes showed sustained IOP reduction with a good safety profile for both groups. There was no significant difference in IOP outcomes between the groups. There may be a small additional reduction in glaucoma medication usage following cataract surgery with iStent inject compared to Hydrus.
Inatani et al 2022	2 years	Patients who had adverse events has been described in the test. 3 had peripheral anterior synechiae formation, 3 had uveitis, 1 had macular oedema, 1 had capsule rupture, 1 had IOL dislocation, 1 had secondary cataract, 1 had vitreous loss, 13 had stent malfunctions (8 had stent occlusions, 5 had stent malpositions), 6 had secondary glaucoma surgery (1 Ahmed valve implantation, 1 iStent removal and Express implantation, 1 iStent removal and Tanito Microhook, 1 iStent removal and trabeculectomy, 2 trabeculectomy)	Introduction: We report 2-year outcomes after implantation of iStent trabecular micro-bypass stent with phacoemulsification, in Japanese patients with mild-moderate open-angle glaucoma (OAG). Methods: This was a 24-month, prospective, longitudinal, observational, post-marketing study conducted between July 2017 and September 2020. Patients consisted of adults with OAG on antiglaucoma medications who had cataract surgery combined with one iStent implantation. Outcome measures included intraocular pressure (IOP), antiglaucoma medications, treatment success rates (defined as eyes having lower IOP with same or reduced number of medications from baseline, or same IOP with reduced number of medications from baseline, and not requiring secondary glaucoma surgeries postoperatively), and safety. Outcomes were analyzed in the overall cohort and in glaucoma subtypes: primary OAG, normal-tension glaucoma, and exfoliative glaucoma. Results: Overall, 232 eyes were enrolled. At 24 months, mean ± standard deviation IOP decreased from 17.6 ± 4.0 mmHg preoperatively to 14.3 ± 3.0 mmHg (p < 0.05), and mean number of medications reduced from 2.2 ± 1.2 preoperatively to 0.7 ± 1.2 (p < 0.05). Similar trends were observed across glaucoma subtypes. In the overall cohort, 96.7%, 95.3%, and 93.7% of patients achieved treatment success at 6, 12, and 24 months, respectively. There were 67.6% medication-free eyes at 24 months compared to 3.2% medication-free eyes preoperatively (p < 0.0001). Safety profile was favorable over the 2-year period. Conclusions: Following iStent implantation with phacoemulsification, clinically relevant and statistically significant reductions in IOP and number of medications were observed in Japanese eyes with OAG over 2 years, with favorable safety profile. These reductions were observed across all glaucoma subtypes.
Katz et al 2018	3.5 years	The study reports that no device-related or sight-threatening adverse events occurred and that no additional glaucoma surgeries were required	Purpose: Evaluate long-term outcomes after one, two, or three trabecular micro-bypass stents implanted in a standalone procedure in eyes with open-angle glaucoma taking ocular hypotensive medication. Patients and methods: Prospective randomized ongoing study of 119 subjects (109 with 42-month follow-up) with open-angle glaucoma, preoperative intraocular pressure (IOP) 18–30 mmHg on one to three glaucoma medications, and unmedicated (post-washout) IOP 22–38 mmHg. Subjects were randomized to receive one (n=38), two (n=41), or three (n=40) iStent trabecular micro-bypass stents in a standalone procedure. Postoperatively, IOP was measured with medication and annually following washout. Data included IOP, medications, gonioscopy, pachymetry, visual field, visual acuity, adverse events, and slit-lamp and fundus examinations. Results: Preoperative mean medicated IOP was 19.8±1.3 mmHg on 1.71 medications in one-stent eyes, 20.1±1.6

			mmHg on 1.76 medications in two-stent eyes, and 20.4±1.8 mmHg on 1.53 medications in three-stent eyes. Post-washout IOP prior to stent implantation was 25.0±1.2, 25.0±1.7, and 25.1±1.9 mmHg in the three groups, respectively. Postoperatively, Month 42 medicated IOP was 15.0±2.8, 15.7±1.0 and 14.8±1.3 mmHg in the three groups, and post-washout IOP (Months 36–37) was 17.4±0.9, 15.8±1.1 and 14.2±1.5 mmHg, respectively. IOP reduction ☐20% without medication was achieved in 89%, 90%, and 92% of one-, two-, and three-stent eyes, respectively, at Month 12; and in 61%, 91%, and 91% of eyes, respectively, at Month 42. The need for additional medication remained consistent at Months 12 and 42 in multi-stent eyes (four two-stent eyes and three three-stent eyes at both time points), whereas it increased in single-stent eyes (four eyes at Month 12 versus 18 eyes at Month 42). Safety parameters were favorable in all groups. Conclusion: The standalone implantation of either single or multiple iStent® device(s) produced safe, clinically meaningful IOP and medication reductions through 42 months postoperatively, with incrementally greater and more sustained reductions in multi-stent eyes.
Lee et al 2020	2 years	In five eyes, the iStent (3) and Hydrus stents (2) were unable to be placed due to inadequate view from bleeding (cases excluded from the data). One patient who received a Hydrus stent developed a hyphema of more than 1.0 mm in the early postoperative period and resolved spontaneously without sequelae (Figures 3a, b). In nine eyes, Hydrus repositioning was performed intraoperatively, with the initial temporal approach unsuccessful and subsequent superior approach insertion. In one eye, the microstent was partially dislocated outside Schlemm's canal postoperatively (Fig. 3c). One iStent patient required trabeculectomy as the IOP was not adequately controlled. There were no adverse visual outcomes associated with combining phacoemulsification with MIGS insertion such as increased incidence of cystoid macula oedema, corneal decompensation or significant refractive error.	Importance: Microinvasive glaucoma surgery (MIGS) combined with phacoemulsification is increasingly utilised in glaucoma management. Objective: To describe the postoperative outcomes of phacoemulsification alone compared to combined phacoemulsification and iStent (Glaukos, San Clemente, CA) or Hydrus (Ivantis, Irvine, CA) for open angle glaucoma. Methods: Retrospective single surgeon comparative case series in a private practice setting. A total of 297 eyes in 190 patients (M:F = 85:105) were included consecutively from March 2011 to June 2017 with the data analysed using linear mixed models. Main outcome measures were intraocular pressure (IOP) and number of medications. Results: By 6 months, all groups showed a reduction in IOP, with the combined iStent group by 4.2 mm Hg and the combined Hydrus group by 4.5 mm Hg, and this trend was sustained with follow-up to two years. All groups also showed a reduction in number of medications, with the phacoemulsification alone cases by 0.3, the combined iStent group by 0.7 and the combined Hydrus group by 1.1 medications. Both the combined surgeries had sustained reduction of number of medications with follow-up to 2 years. Postoperative complications from MIGS device insertion were uncommon and all resolved by one month. Conclusions and Relevance: The combination of phacoemulsification and a MIGS device in open angle glaucoma patients reduced the intraocular pressure and the number of medications by the first postoperative month and had longer-term effects at 2 years follow-up. If the device is inserted without intraoperative complication, the rate of significant short-term risks are low.
Mitchell et al 2024	3 years	While the study does not provide an extensive list of specific post-operative complications, it does report on the incidence of reoperation as a significant long-term outcome. The most common types of reoperation were trabeculectomy and tube shunt surgery.	Objective: Angle-based minimally invasive glaucoma surgery (ab-MIGS) has grown substantially, although long-term efficacy is poorly understood. We analyze ab-MIGS effectiveness with and without preceding laser trabeculoplasty (LTP). Design: Retrospective cohort study. Subjects: Eyes undergoing ab-MIGS (Canaloplasty, Goniotomy, Trabectome, and iStent) with and without prior laser trabeculoplasty (< 2 years preceding MIGS) were identified in the IRIS® Registry (Intelligent Research in Sight) 2013 to 2018. Methods: Propensity score matching (PSM) was undertaken to define the following 4 cohorts: (1) standalone ab-MIGS, no prior LTP vs. (2) standalone ab-MIGS, with prior LTP; and (3) ab-MIGS + phacoemulsification, no prior LTP vs. (4) ab-MIGS + phacoemulsification, with prior LTP. Main Outcome Measures: Failure was defined as subsequent glaucoma reoperation after ab-MIGS (either MIGS or traditional glaucoma surgery). Time-to-event outcome and incidence rates were calculated using survival analysis, and adjusted hazard ratios (aHRs) were generated using multivariate Cox proportional hazards models. Medication data were not available for analysis.<br>Results: A total of 164 965 unique MIGS procedures were performed, from 2013 to 2018. After PSM, we identified 954 eyes undergoing standalone ab-MIGS and 7522 undergoing ab-MIGS + phacoemulsification. For eyes undergoing standalone ab-MIGS, those with prior LTP (n = 477) were more likely to undergo reoperation vs. those without LTP (n = 477) at 6 and 12 months. In multivariable models, those with prior LTP were more likely to undergo reoperation over the 36-month period vs. those without prior LTP (aHR, 1.53; CI, 1.15–2.04; P = 0.004). For eyes undergoing ab-MIGS + phacoemulsification, those with prior LTP (n = 3761) were more likely to undergo reoperation vs. those without LTP (n = 3761) at 12, 24, and 36 months. In multivariable models, those with prior LTP were more likely to undergo reoperation over the 36-month period vs. those without prior LTP (aHR, 1.53 CI, 1.15–2.04; P = 0.004). Conclusions: Prior LTP may be associated with a higher chance of subsequent glaucoma surgery following ab-MIGS, either with or without concurrent phacoemulsification. These findings have important implications for understanding who may benefit most from ab-MIGS, and for guiding patient and surgeon treatment expectations.
Montesano et al 2023	5 years	N - Study states that "it is difficult to predict very long term effects without prolonged follow-up periods".	Purpose: To compare visual field (VF) progression between glaucoma patients receiving cataract surgery alone (CS) or with a Hydrus microstent. Design: Post hoc analysis of VF data from the HORI- ZON multicenter randomized controlled trial. Methods: A total of 556 patients with glaucoma and cataract were randomized 2:1 to either CS-HMS (369) or CS (187) and followed up for 5 years. VF was performed at 6 months and then every year after surgery. We analyzed data for all participants with at least 3 reli- able VFs (false positives < 15%). Average between-group difference in rate of progression (RoP) was tested using a Bayesian mixed model and a 2-sided Bayesian P value < .05 (main outcome). A multivariable model measured the effect of intraocular pressure (IOP). A survival analysis compared the probability of global VF sensitivity drop- ping by predefined cutoffs (2.5, 3.5, 4.5, and 5.5 dB) from baseline. Results: Data from 352 eyes in the CS-HMS arm and 165 in the CS arm were analyzed (2966 VFs). The mean RoP was –0.26 dB/y (95% credible interval –0.36, –0.16) for CS-HMS and –0.49 dB/y (95% credible in- terval –0.63, –0.34) for CS. This difference was significant (P = .0138). The difference in IOP only explained 17% of the effect (P < .0001). Five-year survival analysis showed an increased probability of VF worsening by 5.5 dB (P = .0170), indicating a greater proportion of fast progressors in the CS arm. Conclusions: CS-HMS has a significant effect on VF preservation in glaucoma patients compared with CS alone, reducing the proportion of fast progressors.
Oberfeld et al 2023	3 years	One PEcK case was complicated by rebound iritis. There were a few cases (3) where hyphema or microhyphema persisted up to 6 months. There were 10 cases of postoperative cystoid macular edema (CME) in patients who underwent either PEcK or Phaco/ECP. there were a few cases (3) where corneal edema persisted for a longer period (up to 18 months). The study did not identify any specific long-term complications that persisted beyond the follow-	Purpose: Combining two or more MIGS (cMIGS) promises to be more efficacious than single MIGS (sMIGS). This study compared the efficacy of PEcK, which combines Phacoemulsification (Phaco), Endocyclophotocoagulation (ECP), and Kahook dual blade (KDB), relative to both of its constituent sMIGS, Phaco/ECP (Endo Optiks, NJ) and Phaco/KDB (New World Medical, CA) for the f irst time. Patients and methods: Data was collected retrospectively from 1833 visits of 271 patients who underwent PEcK, Phaco/ECP, or Phaco/KDB from 2016–2021 at Massachusetts Eye and Ear. Primary outcomes included Generalized Estimating Equations (GEE) of intraocular pressure (IOP) and medication burden, as well as survival models. Results: Mean preoperative IOP was 17.6 ± 5.0 (SD) mmHg on 3.0 ± 1.4 medications in the PEcK group (n = 128), 17.9 ± 5.1 mmHg on 2.2 ± 1.5 medications in the Phaco/ECP group (n = 78), and 16.1 ± 4.3 mmHg on 0.4 ± 1.0 medications in the Phaco/KDB group (n = 65). For more than 36 months, all procedures resulted in significant patterns of IOP and medication reduction (all p < 0.001), before and after

		up period, or any novel or unexpected complications related to the combination of procedures in PEcK	statistical adjustment. The reduction pattern in IOP was significantly different when comparing all groups over time and favored PEcK (p = 0.04), but the reduction pattern in medications was not significantly different (p = 0.11). Procedures did not differ in procedural time (p = 0.18) or in survival to maintain ≥20% IOP reduction (p = 0.43) without additional medication or procedure. There was a trend toward significant difference in maintaining IOP ≤ goal IOP that favored PEcK over Phaco/ECP after adjustment (p = 0.09). Conclusion: PEcK may confer greater IOP reduction without added procedural time compared to Phaco/ECP and Phaco/KDB in predominantly mild or moderate glaucoma. Further research on cMIGS may benefit from adopting this comparative analysis to constituent MIGS.
Oberfeld, Helwe et al 2023	3 years	Post-operative corneal oedema or inflammation was common, resolving within 6 weeks for most patients. However, there were 3 cases where this persisted for up to 18 months. This complication, which is bleeding in the anterior chamber of the eye, occurred in one patient after Phaco/Hydrus and in five patients after Phaco/KDB. These cases of hyphema resolved prior to 6 weeks post-operation. One Phaco/KDB patient experienced acute posterior vitreous detachment 8 weeks post-operatively. This was in the context of persistent inflammation and extended use of prednisolone 1% eyedrops. Two patients experienced post-operative cystoid macular oedema following Phaco/Hydrus. Additional surgical intervention was performed for 2% of total eyes within 6 months post-operatively. This included trabeculectomy for one Phaco/Hydrus patient and glaucoma drainage implant for two Phaco/KDB patients. One Phaco/KDB patient underwent laser trabeculoplasty within 8 months.	Purpose. — To report the relative efficacy of combining phacoemulsification with a Schlemm's canal microstent (Phaco/Hydrus) or dual blade trabecular excision (Phaco/KDB). Design. — Retrospective study. Methods. — One hundred and thirty-one eyes of 131 patients who underwent Phaco/Hydrus or Phaco/KDB procedures from January 2016 to July 2021 at a tertiary care center were included and assessed for up to 36 months postoperatively. Primary outcomes were intraocular pressure (IOP) and number of glaucoma medications, evaluated by generalized estimating equations (GEE). Two Kaplan—Meier estimates (KM) assessed survival without additional intervention or pressure lowering medication while maintaining: (1) IOP ≤ 21 mmHg and ≥ 20% IOP reduction or (2) IOP ≤ preoperatively designated goal.Results. — Mean preoperative IOP was 17.70 ± 4.91 (SD) mmHg on 0.28 ± 0.86 medications in the Phaco/Hydrus cohort (n = 69) and 15.92 ± 4.34 mmHg on 0.19 ± 0.70 medications in the Phaco/KDB cohort (n = 62). At 12 months, mean IOP was reduced to 14.98 ± 2.77 mmHg on 0.12 ± 0.60 medications after Phaco/Hydrus and 13.52 ± 4.13 mmHg on 0.04 ± 0.19 medications after Phaco/KDB. GEE models of IOP (P < 0.001) and medication burden (P < 0.05) showed significant patterns of reduction across all timepoints in both cohorts. There were no differences in IOP reduction (P = 0.94), number of medications (P = 0.95) or survival (P = 0.72 by KM1, P = 0.11 by KM2) between procedures. Conclusions. — Both Phaco/Hydrus and Phaco/KDB resulted in significantly reduced IOP and medication burden for over 12 months. Phaco/Hydrus and Phaco/KDB confer similar outcomes in terms of IOP, medication burden, survival, and procedural time in a population with predominantly mild and moderate open-angle glaucoma.
Reitsamer et al 2022	3 years	Persistent hypotony occurred in 1 (0.5%) eye. Clinical hypotony occurred in 4 (1.9%) eyes. Implant exposure or extrusion occurred in 4 (1.9%) eyes. Implant fracture occurred in 2 (0.9%) eyes. Implant migration occurred in 2 (0.9%) eyes. Implant repositioning requiring surgical intervention occurred in 3 (1.4%) eyes.Implant touching the iris occurred in 5 (2.4%) eyes.<br>Other complications: Macular oedema was reported in 1 (0.5%) eye. Macular hole was reported in 1 (0.5%) eye. The study also recorded other postoperative AESIs, including shallow anterior chamber with iridocorneal touch (3 eyes), bleb leak (1 eye), corneal decompensation/oedema (<30 days) (6 eyes), dacryocystitis (1 eye), endophthalmitis (1 eye), hyphema (4 eyes), periorbital cellulitis (1 eye), ptosis (1 eye), retinal vein occlusion (1 eye), scleritis (1 eye), ulcerative keratitis (1 eye) and vitreous haemorrhage (2 eyes). These complications were identified from a retrospective review, and the study notes that the anonymized nature of the data collection limited the ability to ascertain the specific details and causality of these events.	Purpose: To assess the 3-year effectiveness and safety of the XEN gel stent implanted ab interno in open-angle glaucoma (OAG). Methods: This study was a multicentre, retrospective chart review of consecutive patients with OAG who underwent ab-interno gel stent placement alone or combined with phacoemulsification between 1 January 2014 and 1 October 2015. Outcome measures included mean changes in intraocular pressure (IOP) and IOP-lowering medication count from medicated baseline at 1, 2, 3 (primary outcome) and 4 years (if available) postimplantation. Intraoperative complications, adverse events of special interest (AESIs) and secondary surgical interventions (SSIs) were recorded.<br>Results: The safety and effectiveness populations included 212 eyes (primary and secondary) and 174 eyes (primary), respectively. Mean IOP and medication decreased from 20.7 mmHg and 2.5 at baseline (n = 163 primary/first implanted eyes) to 13.9 mmHg and 1.1 medications (n = 76) at 3 years postimplantation, respectively. Mean changes from baseline in IOP (−5.6, −6.2 and −6.6 mmHg) and IOP-lowering medication count (−1.8, −1.6 and −1.4) were statistically significant at 1, 2 and 3 years postimplantation, respectively. Results appeared comparable when implantation was performed with (n = 76) or without (n = 98) phacoemulsification. In primary eyes with 4-year IOP and medication count data (n = 27), mean IOP was 14.0 mmHg on 1.3 medications at 4 years postimplantation. Fifteen (7.1%) eyes had intraoperative complications, 31 (14.6%) experienced 46 postoperative AESIs, and 26 (12.3%) required SSI. Conclusion: The gel stent effectively lowered IOP and IOP-lowering medication count over 3 years, with a predictable and acceptable safety profile, when implanted via the traditional ab-interno technique.
Salimi et al 2021	3 years	The study mentions several post-operative complications, most of which were rare and transient:<br>IOP spikes (within the first 2 postoperative weeks); Corneal edema (mostly on the first post-operative day); Transient microhyphema (within the first 24 hours); Rebound iritis (1 month post-operatively); Epiretinal membrane; Stent covered by a fibrotic membrane; Cystoid macular edema; Acute anterior uveitis (1 week post-operatively). The study notes that there were no reports of hypotony, choroidal detachment, stent extrusion, peripheral anterior synechiae, myopic shift or endophthalmitis.	Precis: This longitudinal case series provides valuable long-term data on the safety and efficacy of iStent inject with concomitant cataract surgery. Patients experienced significant and durable reductions in both intraocular pressure (IOP) and medication burden through 3 years postoperative.<br>Purpose: The purpose of this study was to examine the 3-year effectiveness and safety of iStent inject second-generation trabecular micro-bypass stent implantation with concomitant phacoemulsification cataract surgery in eyes with various glaucoma subtypes and severities.<br>Methods: This was a single-center consecutive case series. Three-year outcomes included mean IOP and medications, the proportions of eyes with IOP ≤18, ≤15, and ≤12 mm Hg, and success (absence of secondary glaucoma interventions). Safety included best-corrected visual acuity (BCVA), cup-to-disc ratio, visual field (VF) mean deviation, retinal nerve fiber layer (RNFL), and ganglion cell-inner plexiform layer (GCIPL) thickness, and adverse events.<br>Results: A total of 124 eyes with different glaucoma subtypes and severities were included. At 3 years postoperative, mean IOP reduced from 16.90±3.85 mm Hg preoperatively to 13.17±2.83 mm Hg (22% reduction, P<0.001) and mean medication burden decreased from 2.38±1.29 medications preoperatively to 1.16±1.22 medications (51% reduction, P<0.001). At 3 years, 96% of eyes achieved IOP ≤18 mm Hg (vs. 69% preoperatively), 80% of eyes achieved IOP ≤15 mm Hg (vs. 40% preoperatively), and 42% of eyes achieved IOP ≤12 mm Hg (vs. 7% preoperatively) with 76% of eyes eliminating ≥1 medication and 37% of eyes eliminating ≥2 medications versus preoperative medication burden. The 3-year cumulative survival rate was 74%. Postphacoemulsification BCVA improvement was preserved, and cup-to-disc ratio, VF mean deviation, and RNFL and GCIPL thickness remained stable. A favorable safety profile included no intraoperative complications and few, transient, postoperative adverse events.<br>Conclusion: Significant and sustained IOP and medication reductions were achieved through 3 years after iStent inject implantation with cataract surgery in a real-world clinical population with mild-to-severe glaucoma, along with favorable safety including stable BCVA, VF, and RNFL and GCIPL thickness.

Samuelson et al 2019	2 years	The study notes that there were no cases of hypotony at 1 month postoperatively or later, and no reports of stent migration or stent dislocation. The study also notes that most complications were comparable between the two groups and consistent with complications expected from cataract surgery in a similar population.Ocular surface disease was seen in 16.1% of iStent inject group and 16.8% of the control group.Stent obstruction was seen in 6.2% of iStent inject group.Intraocular inflammation was seen in 5.7% of the treatment group and 4.2% of the control group.Secondary surgical intervention was required in 5.4% of the treatment group and 5.0% of the control group.Other complications such as ocular allergies, loss of BSCVA, posterior vitreous detachment, blurred vision, epiretinal membrane, IOP increase, corneal abrasion, and corneal opacity were observed, but generally at similar rates in both groups, or at rates below 5%.The most substantial decline in endothelial cell density occurred within the first 3 months postoperatively, but this was consistent with cataract surgery alone, with minimal decline thereafter.Focal goniosynechiae (PAS) was reported in 1.8% of the treatment eyes. No cases of endophthalmitis, hypotony maculopathy, or other serious complications were reported.	Purpose: Evaluate the safety and effectiveness of an ab interno implanted (iStent inject) Trabecular MicroBypass System (Glaukos Corporation, San Clemente, CA) in combination with cataract surgery in subjects with mild to moderate primary open-angle glaucoma (POAG). Design: Prospective, randomized, single-masked, concurrently controlled, multicenter clinical trial. Participants: Eyes with mild to moderate POAG and preoperative intraocular pressure (IOP) 24 mmHg on 1 to 3 medications, unmedicated diurnal IOP (DIOP) 21 to 36 mmHg, and cataract requiring surgery. Methods: After uncomplicated cataract surgery, eyes were randomized 3:1 intraoperatively to ab interno implantation of iStent inject (Model G2-M-IS; treatment group, n ¼ 387) or no stent implantation (control group, n ¼ 118). Subjects were followed through 2 years postoperatively. Annual washout of ocular hypotensive medication was performed. Main Outcome Measures: Effectiveness end points were 20% reduction from baseline in month 24 unmedicated DIOP and change in unmedicated month 24 DIOP from baseline. Safety measures included best spectacle-corrected visual acuity (BSCVA), slit-lamp and fundus examinations, gonioscopy, pachymetry, specular microscopy, visual fields, complications, and adverse events. Results: The groups were well balanced preoperatively, including medicated IOP (17.5 mmHg in both groups) and unmedicated DIOP (24.8 3.3 mmHg vs. 24.5 3.1 mmHg in the treatment and control groups, respectively, P ¼ 0.33). At 24 months, 75.8% of treatment eyes versus 61.9% of control eyes experienced 20% reduction from baseline in unmedicated DIOP (P ¼ 0.005), and mean reduction in unmedicated DIOP from baseline was greater in treatment eyes (7.0 4.0 mmHg) than in control eyes (5.4 3.7 mmHg; P < 0.001). Of the responders, 84% of treatment eyes and 67% of control eyes were not receiving ocular hypotensive medication at 23 months. Furthermore, 63.2% of treatment eyes versus 50.0% of control eyes had month 24 medication-free DIOP 18 mmHg (difference 13.2%; 95% confidence interval, 2.9e23.4). The overall safety profile of the treatment group was favorable and similar to that in the control group throughout the 2-year follow-up. Conclusions: Clinically and statistically greater reductions in IOP without medication were achieved after iStent inject implantation with cataract surgery versus cataract surgery alone, with excellent safety through 2 years.
Samuelson et al 2021	2 years	The study does not specifically discuss long-term complications such as hypotony, bleb leaks or endophthalmitis, or other complications that may be associated with glaucoma surgery in general.	Objective: To assess quality of life (QOL) as mea- sured by patient-reported outcomes (PRO) within the iS- tent inject ® pivotal trial. Design: Randomized controlled trial analysis of sec- ondary outcomes. Methods: The Vision Function Questionnaire (VFQ- 25) and Ocular Surface Disease Index (OSDI) question- naire were administered at baseline and at months 1, 6, 12, and 24. PRO responders were defined as patients reaching improvement based on minimally important dif- ferences. • Results: A total of 505 patients were randomized (386 iStent inject ® [Glaukos], 119 surgery alone). The iStent inject ® group exhibited a greater percentage of PRO re- sponders across all follow-up visits over 24 months, aver- aging 58.0% vs 45.8%; P < .05 for VFQ-25 composite scores and 56.7% vs 48.9%; P < .05 for OSDI compos- ite scores. Odds of being a responder in the iStent inject ® group was 60% (P < .05) higher for the VFQ-25 and 32% (P > .05) higher for the ODSI. Driving (49.0% vs 28.8%; P < .05), ocular pain (59.3% vs 47.2%; P < .05), and general vision (71.8% vs 60.0%; P < .05) were the VFQ-25 subscales responsible for differences between treatment groups. At month 24, 76.5% of VFQ- 25 responders and 62.5% of nonresponders were medica- tion free regardless of treatment group (P < .05). Conclusions: Exploratory analysis suggests that by reducing medication dependence, implantation with the micro-scale iStent inject ® device with cataract surgery may improve QOL vs cataract surgery alone over 24 months, with improvements influenced by ocular symptoms and vision-related activities.
Schlenker et al 2017	4 years	Y, There were 22 and 30 distinct complications in the microstent and trabeculectomy group, respectively.Eighteen eyes (9.7%) and 27 eyes (16.0%) experienced a largely treatable complication after the first month, most commonly a leak or dehiscence that resolved with conservative measures. Complications from hypotony after the first month were low for both groups. Four microstent eyes and 2 trabeculectomy eyes demonstrated malignant glaucoma. One trabeculectomy eye demonstrated blebitis that resolved with conservative treatment.	Purpose: To compare the efficacy, safety, and risk factors for failure of standalone ab interno gelatin microstent implantation with mitomycin C (MMC) versus trabeculectomy with MMC. Design: International, multicenter, retrospective interventional cohort study. Participants: Three hundred fifty-four eyes of 293 patients (185 microstent and 169 trabeculectomy) with no prior incisional surgery.Methods: Consecutive eyes with uncontrolled glaucoma underwent microstent or trabeculectomy surgery from January 1, 2011 through July 31, 2015 at 4 academic ophthalmology centers: Toronto, Canada; Frankfurt, Germany; Salzburg, Austria; and Leuven, Belgium. Main outcome measures: Primary outcome measure was hazard ratio (HR) of failure, with failure defined as 2 consecutive intraocular pressure (IOP) readings of <6 mmHg with vision loss or >17 mmHg without glaucoma medications (complete success) at least 1 month after surgery despite in-clinic interventions (including needling). Secondary outcome measures included IOP thresholds of 6 to 14 mmHg and 6 to 21 mmHg and same thresholds allowing for medications (qualified success), interventions, complications, and reoperations. Results: Baseline characteristics were similar, except more men (56% vs. 43%), younger patients (average, by 3 years), better preoperative visual acuity (22% vs. 32% with 0.4 logarithm of the minimum angle of resolution vision or worse), and more trabeculoplasty (52% vs. 30%) among microstent eyes. The adjusted HR of failure of the microstent relative to trabeculectomy was 1.2 (95% confidence interval [CI], 0.7-2.0) for complete success and 1.3 (95% CI, 0.6-2.8) for qualified success, and similar for other outcomes. Time to 25% failure was 11.2 months (95% CI, 6.9-16.1 months) and 10.6 months (95% CI, 6.8-16.2 months) for complete success and 30.3 months (95% CI, 19.0-∞ months) and 33.3 months (95% CI, 25.7-46.2 months) for qualified success. Overall, white ethnicity was associated with decreased risk of failure (adjusted HR, 0.49; 95% CI, 0.25-0.96), and diabetes was associated with increased risk of failure (adjusted HR, 4.21; 95% CI, 2.10-8.45). There were 117 and 165 distinct interventions: 43% and 31% underwent needling, respectively, and 50% of trabeculectomy eyes underwent laser suture lysis. There were 22 and 30 distinct complications, although most were transient. Ten percent and 5% underwent reoperation (P = 0.11). Conclusions: There was no detectable difference in risk of failure and safety profiles between standalone ab interno microstent with MMC and trabeculectomy with MMC.
Strzalkowska et al 2021	2 years	9% of AIT patients required additional surgery. The most common secondary intervention was the need for a tube shunt in 15 patients. While both procedures have potential long-term complications, TRAB is associated with a higher risk of secondary interventions and other specific complications compared to AIT. AIT is a lower risk procedure but has a higher rate of not meeting the criteria for success, and when it fails, it often needs a more invasive procedure such as a tube shunt.	Purpose: We used exact matching for a highly balanced comparison of ab interno trabeculectomy (AIT) with the trabectome to trabeculectomy with mitomycin C (TRAB).Methods: A total of 5485 patients who underwent AIT were exact-matched to 196 TRAB patients by baseline intraocular pressure (IOP), number of glaucoma medications, and glaucoma type. Nearest-neighbor–matching was applied to age. Success was defined as a final IOP of less than 21 mmHg, IOP reduction of at least 20% reduction from baseline, and no secondary surgical interventions. Outcomes were measured at 1, 3, 6, 12, 18, and 24 months. Results: A total of 165 AIT could be matched to 165 TRAB. The mean baseline IOP was 22.3 ± 5.6 mmHg, and the baseline number of glaucoma medications was 2.7 ± 1.1 in both groups. At 24 months, IOP was reduced to 15.8 ± 5.2 mmHg in AIT and 12.4 ± 4.7 mmHg in TRAB. IOP was lower than baseline at all visits (p < 0.01) and lower in TRAB than AIT (p < 0.01). Glaucoma medications were reduced to 2.1 ± 1.3 in AIT and 0.2 ± 0.8 in TRAB. Compared to baseline, patients used fewer drops

			postoperatively (p < 0.01) and more infrequently in TRAB than in AIT (p > 0.01). Secondary surgical interventions had the highest impact on success and became necessary in 15 AIT and 59 TRAB patients. Thirty-two challenging events occurred in TRAB and none in AIT. Conclusion: Both AIT and TRAB reduced IOP and medications. This reduction was more significant in TRAB but at the expense of four times as many secondary interventions.
Tojo et al 2020	2 years	An anterior chamber hemorrhage is the most common complication of trabectome surgery. All patients were observed intraoperative hyphema that is due to reverse flow from episcleral venous. There was no patient who needed irrigation of the anterior chamber additionally. No serious vision-threatening complications (eg, hypotonic maculopathy, rhegmatogenous retinal detachment, or endophthalmitis) occurred.	Background: We compared the efficiency of trabectome surgery for patients with differing preoperative intraocular pressure (IOP) values in a single-facility retrospective study. We evaluated surgical outcomes based on three grades of preoperative IOP: high (hi-IOP, >26 mmHg), middle (mid-IOP, 18-26 mmHg), and low (lo-IOP, <18 mmHg).Patients and methods: We analyzed the cases of 204 eyes that underwent trabectome surgery and were followed >2 years. We defined failure as a reduction of IOP <20% or requiring additional glaucoma surgery. We used 4 cutoff values >21, >18, >15, and >12 mmHg. Other factors that may affect surgical outcomes were also investigated: age, central corneal thickness (CCT), history of selective laser trabeculotrabculoplasty (SLT), preoperative visual field, and simultaneous cataract surgery. Results: Trabectome surgeries significantly decreased the IOP values from 23.0 ± 7.2 mmHg to 13.6 ± 3.6 mmHg at 2 years post-surgery. The mid-IOP group achieved significantly better surgical outcomes than the lo-IOP and hi-IOP groups with cutoff values 21, 18, and 15 mmHg. A thin CCT and simultaneous cataract surgery were significantly related to better surgical outcomes with cutoff value 21.18 and 15 mmHg. Conclusion: For the patients with preoperative IOP <18 mmHg, it was difficult to decrease their IOP >20% with trabectome surgery. Patients with preoperative IOP values >26 mmHg often required additional glaucoma surgery.
Vukmirovic et al 2023	2 years	Hyphaema: This occurred in 15.6% of eyes overall. Dellen: The overall rate of dellen was 3.1%. Hypotony with shallow anterior chamber: This complication occurred in 6.1% of eyes. Hypotony requiring viscoelastic fill: This occurred in 0.4% of eyes. Choroidal effusions: These were observed in 3.8% of eyes overall. The study notes that these complication rates for the gelatin stent surgery appear to be lower compared to those reported in a UK national survey of trabeculectomy complications. The study also suggests that the large size of the introducer needle (300 micron) compared to the XEN stent outer diameter (90 micron) may contribute to peri-stent leak in the post-operative period. The study does not describe timeline of complications.	Background: The main objectives of this study were to determine whether known risk factors for trabeculectomy failure similarly influence gelatin stent outcomes and to identify surgical factors which may optimise success. Methods: A retrospective, observational study was conducted at a single centre in Perth, Western Australia over 24 months. Two-hundred and sixty-two eyes of 207 patients underwent XEN-45 stent surgery with various forms of glaucoma. Surgical and postoperative data on subjects undergoing XEN-45 stent surgery was collated. Intraocular pressure (IOP) reduction success was determined using three criteria: 1; IOP <18mm Hg, 2: IOP <15mm Hg and 3: >25% IOP reduction from baseline. Kaplan–Meier, mixed effects Cox Proportional hazard model and Chi-Square test were used to measure survival of functioning stents. Results: The success rates at a maximum of 2years after surgery by criteria 1, 2 and 3 were 61.3%, 26.2% and 28.9% in primary open angle glaucoma (n = 243), 18.8%, 16.9%, 21.4% in angle closure glaucoma (n = 11), 0%, 0%, 66.7% in congenital glaucoma (n = 5) and 0% in uveitic glaucoma (n = 3). No significant reduction in success was found in those eyes that had prior ocular surgery (all p>0.07). Conclusions: Prior cataract or trabeculectomy surgery does not appear to adversely affect gelatin stent outcomes over 2years follow up. Gelatin stent surgery appears to have less IOP reduction effect compared to trabeculectomy at 2years.
Yang et al 2023	3 years	MIGS procedures were associated with low total documented complication rates: 1% for ECP, 1% for Xen implantation, and 2% for goniotomy or canaloplasty. Specific complications looked at included Hyphaema, corneal oedema, iridocyclitis, vitreous haemmorhage, etc but only within 90 days of having procedure.	Objective: To evaluate the effectiveness of micro-invasive glaucoma surgery (MIGS) with and without concurrent phacoemulsification. Design: Multicenter retrospective cohort study. Participants: Patients in the IRIS® Registry (Intelligent Research in Sight) who underwent Xen gel stent (ab interno), endoscopic cyclophotocoagulation (ECP), goniotomy, or canaloplasty from 2013 through 2019. Methods: Kaplan-Meier survival analysis was used to assess reoperation rates. We defined reoperation as any subsequent glaucoma surgery occurring 1 month to 3 years after the initial procedure. Multivariable cox proportional hazard models were used to determine factors predictive of reoperation. Main outcome measures: reoperation rate, mean intraocular pressure (IOP) and visual acuity (VA), postoperative complications, predictors of reoperation, and reoperation procedure type. Results: A total of 79,363 eyes from 57, 561 patients were included with 15,118 eyes (19 %) receiving stand-alone MIGS and 64,245 eyes (81%) receiving MIGS concurrent with phacoemulsification. Overall, patients who underwent MIGS concurrently with phacoemulsification had lower reoperation rates compared to those undergoing stand-alone MIGS. This was most pronounced for the ECP and goniotomy/canaloplasty groups. At two years postoperatively, the cumulative reoperation rate when a procedure was performed alone (without cataract surgery) was 15% for ECP, 24% for Xen, and 24% for goniotomy/canaloplasty compared to 3% for ECP, 19% for Xen, and 6% for goniotomy/canaloplasty performed concurrently with phacoemulsification (p<0.001 for each stand-alone MIGS vs MIGS with phacoemulsification). Black race, older age, coding diagnosis of moderate and severe glaucoma, higher baseline IOP, and glaucoma subtype were associated with higher reoperation risk. While IOP decreased in all groups, those who underwent stand-alone MIGS had a more substantial decrease in mean IOP. MIGS complication rates based on diagnosis codes were low overall: 1% for ECP, 1% for Xen, and 2% for goniotomy/canaloplasty. Conclusions: In current US clinical practice, MIGS has substantially lower reoperation rates when performed with phacoemulsification, especially for ECP and goniotomy/canaloplasty. Approximately one-sixth of patients undergoing stand-alone ECP and one quarter of patients undergoing stand-alone Xen or goniotomy/canaloplasty require reoperation by 2 years. Black patients, diagnosis coding of moderate to severe glaucoma, and higher baseline IOP were associated with higher risk of reoperation after MIGS procedures.
