# Supplementary material for: Evaluating Long-Term Effectiveness and Safety of Glaucoma Surgical Innovations: A Systematic Review
Source: J Glaucoma. 2026 Oct 16;35(11):838–45. doi: 10.1097/IJG.0000000000002770 (PMC13722426; doi:10.1097/IJG.0000000000002770)
Supplement: Supplementary file 9 [file ijg-35-838-s009.pdf]

Supplementary Table 9. Newcastle Cohort Risk of Bias for cohort studies

First Author (Year)	Oberfeld et al (2023)	Schlenker et al 2017	Strzalkowska et al 2020	Yang et al 2023
**Representativeness of the exposed cohort**<br>a) truly representative of the average _____________ (describe) in the community *<br>b) somewhat representative of the average ____________ in the community *<br>c) selected group of users eg nurses, volunteers<br>d) no description of the derivation of the cohort	b) somewhat representative of the average patient receiving procedures in the community *	b) somewhat representative of the average patient with glaucoma *	b) somewhat representative of the average AIT procedures in the community as dataset taken from a database *	*a) Participants truly representative of patients with glaucoma in the USA. IRIS registry includes nearly 24% of the US population and data is collected from electronic health records.
**Selection of the non exposed cohort**<br>a) drawn from the same community as the exposed cohort *<br>b) drawn from a different source<br>c) no description of the derivation of the non exposed cohort	a) drawn from the same community as the exposed cohort *	a) drawn from the same community as the exposed cohort*	b) drawn from a different source	c) No non exposed cohort
**Ascertainment of exposure**<br>a) secure record (eg surgical records) *<br>b) structured interview *<br>c) written self report<br>d) no description	a) secure record (eg surgical records) *	a) secore records*	d) no description	*a) secure record - HIPAA complaint EHR data
**Demonstration that outcome of interest was not present at start of study**<br>a) yes *<br>b) no	a) yes *	a) yes *	a) yes	*a) Yes, patients with two or more MIGs excluded
**Comparability of cohorts on the basis of the design or analysis**<br>a) study controls for ___________ (select the most important factor) *<br>b) study controls for any additional factor * (This criteria could be modified to indicate specific control for a second important factor.)	a) study controls for missing data*	a) study controls for prior incisional surgery*	Study does not control for any factors	*a) Study controls for baseline demographic comparability by stratifying distribution of demographic and baseline ocular characteristics
**Assessment of outcome**<br>a) independent blind assessment (ie. Professionals unaware which cohort patients belong to) *<br>b) record linkage (using existing administrative or health records to obtain outcome data) *<br>c) self report (patients report outcomes themselves or researchers directly assess outcomes without blinding)<br>d) no description	b) record linkage (using existing administrative or health records to obtain outcome data) *	b) record linkage*	d) no description	*b) Record linkage
**Was follow-up long enough for outcomes to occur**<br>a) yes (>2 year follow up period) *<br>b) no	a) yes (>2 year follow up period) *	a) yes*	a) yes*	*a) Yes (2 years follow up)
**Adequacy of follow up of cohorts**<br>a) complete follow up - all subjects accounted for *<br>b) subjects lost to follow up unlikely to introduce bias - small number lost - > ____ % (select an adequate %) follow up, or description provided of those lost) *<br>c) follow up rate < ____% (select an adequate %) and no description of those lost<br>d) no statement	c) follow up rate < 30% (select an adequate %) and no description of those lost	d) no statement	d) no statement	b) All patients with missing data excluded as part of exclusion criteria. This was 121, 523 eyes - a fairly large number
**Quality score**	7 stars = Good quality	7 stars = Good quality	2 stars - poor quality	6 stars - Good Quality
