# Supplementary material for: Evaluating Long-Term Effectiveness and Safety of Glaucoma Surgical Innovations: A Systematic Review
Source: J Glaucoma. 2026 Oct 16;35(11):838–45. doi: 10.1097/IJG.0000000000002770 (PMC13722426; doi:10.1097/IJG.0000000000002770)
Supplement: Supplementary file 10 [file ijg-35-838-s010.pdf]

Supplementary Table 10. JBI Case Series Risk of Bias for case series

First Author (Year)	Arnould et al 2024	Bambo et al 2024	Clement et al 2020	Dang et al 2018	Dietz et al 2023	Ferguson et al 2020	Gabbay et al 2021	Hengerer et al 2022	Holmes et al (2022)	Inatani et al 2022	Lee et al (2019)	Mitchell et al 2024	Oberfeld, Helwe et al (2023)	Reitsamer et al 2022	Salimi et al 2021	Tojo et al 2020	Vukmirovic et al 2022
Were there clear criteria for inclusion in the case series?	Unclear	Y	Y	Y	Y	Y	Y	Y	Y	Y	Y	Unclear	Y	Y	Y	N	Y
Was the condition measured in a standard, reliable way for all participants included in the case series?	Y	Y	Y	Y	Y	Y	Y	Y	Y	Y	N	Y	Y	N	Y	Y	Y
Were valid methods used for identification of the condition for all participants included in the case series?	Y	Y	Y	N	Y	Y	Y	Y	Y	Y	Y	Y	Y	Unclear	Y	Y	Unclear
Did the case series have consecutive inclusion of participants?	Y	Y	Unclear	Unclear	Y	Y	Unclear	Y	Unclear	Unclear	Y	Unclear	N	Y	Y	Y	Unclear
Was there clear reporting of the demographics of the participants in the study?	Y	Y	Y	Yes	Y	Y	Y	Y	Y	Y	Y	Y	Y	Y	Y	Y	Y
Was there clear reporting of clinical information of the participants?	Y	Y	Y	Y	Y	Y	Y	Y	Y	Y	Y	Y	Y	Y	Y	Y	Y
Were the outcomes or follow up results of cases clearly reported?	Y	Y	Y	Y	Y	Y	Y	Y	Y	Y	Y	Y	Y	Y	Y	Y	Y
Was there clear reporting of the presenting site(s)/clinic(s) demographic information?	Y	N	N	N	Y	N	Y	Y	N	Y	Y	Y	Y	Y	Y	Y	Y
Was statistical analysis appropriate?	Y	Y	Y	Y	Y	Y	Y	Y	Y	Y	Y	Y	Y	Y	Y	Y	Y
