# Supplementary material for: Evaluating Long-Term Effectiveness and Safety of Glaucoma Surgical Innovations: A Systematic Review
Source: J Glaucoma. 2026 Oct 16;35(11):838–45. doi: 10.1097/IJG.0000000000002770 (PMC13722426; doi:10.1097/IJG.0000000000002770)
Supplement: Supplementary file 11 [file ijg-35-838-s011.pdf]

Supplementary Table 11. Cochrane Risk of Bias 2 for Randomised Controlled Trials

	**First Author (Year)**	**Samuelson et al, 2021**	**Montesano et al 2023 (same trial as Ahmed et al 2022)**	**Ahmed et al 2022**	**Samuelson et al 2019**	**Katz et al 2018**	**Ahmed et al 2023**
Domain 1. Risk of bias arising from the randomisation process	Risk of bias judgement for Domain 1	Low risk	Low risk	Low risk	Low risk	High risk	High risk
Domain 2. Risk of bias due to deviations from the intended interventions (effect of assignment to intervention)	Risk of bias judgement for Domain 2	Low risk	Low risk	Low risk	Low risk	Some concerns	Low risk
Domain 3: Risk of bias due to missing outcome data	Risk of bias judgement for Domain 3	Low risk	Some concerns	Some concerns	Some concerns	Low risk	Low risk
Domain 4: Risk of bias in measurement of the outcome	Risk of bias judgement for Domain 4	Some concerns	High risk	High risk	Low risk	Low risk	Low risk
Domain 5: Risk of bias in selection of the reported result	Risk of bias judgement for Domain 5	Low risk	Low risk	Low risk	Low risk	Low risk	Low risk
